# Accelerated Conversion of Polysulfides for Ultra Long‐Cycle of Li‐S Battery at High‐Rate over Cooperative Cathode Electrocatalyst of Ni_0.261_Co_0.739_S_2_/N‐Doped CNTs

**DOI:** 10.1002/advs.202402389

**Published:** 2024-06-12

**Authors:** Junhyuk Ji, Minseon Park, Minho Kim, Song Kyu Kang, Gwan Hyeon Park, Junbeom Maeng, Jungseub Ha, Min Ho Seo, Won Bae Kim

**Affiliations:** ^1^ Department of Chemical Engineering Pohang University of Science and Technology (POSTECH) 77 Cheongam‐ro, Nam‐gu Pohang‐si Gyeongsangbuk‐do 37673 Republic of Korea; ^2^ Department of Nanotechnology Engineering Pukyong National University (PKNU) 45 Yongso‐ro, Nam‐gu Busan‐si 48513 Republic of Korea; ^3^ Graduate Institute of Ferrous & Eco Materials Technology Pohang University of Science and Technology (POSTECH) 77 Cheongam‐ro, Nam‐gu Pohang‐si Gyeongsangbuk‐do 37673 Republic of Korea

**Keywords:** cooperative cathode catalysts, Li‐S battery, lithium polysulfides, N‐doped porous carbon, nickel cobalt sulfide

## Abstract

Despite the very high theoretical energy density, Li‐S batteries still need to fundamentally overcome the sluggish redox kinetics of lithium polysulfides (LiPSs) and low sulfur utilization that limit the practical applications. Here, highly active and stable cathode, nitrogen‐doped porous carbon nanotubes (NPCTs) decorated with Ni_x_Co_1‐x_S_2_ nanocrystals are systematically synthesized as multi‐functional electrocatalytic materials. The nitrogen‐doped carbon matrix can contribute to the adsorption of LiPSs on heteroatom active sites with buffering space. Also, both experimental and computation‐based theoretical analyses validate the electrocatalytic principles of co‐operational facilitated redox reaction dominated by covalent‐site‐dependent mechanism; the favorable adsorption‐interaction and electrocatalytic conversion of LiPSs take place subsequently by weakening sulfur‐bond strength on the catalytic Ni_Oh_^2+^−S−Co_Oh_^2+^ backbones via octahedral TM‐S (TM = Ni, Co) covalency‐relationship, demonstrating that fine tuning of Co_Oh_^2+^ sites by Ni_Oh_^2+^ substitution effectively modulates the binding energies of LiPSs on the Ni_x_Co_1‐x_S_2_@NPCTs surface. Noteworthy, the Ni_0.261_Co_0.739_S_2_@NPCTs catalyst shows great cyclic stability with a capacity of up to 511 mAh g^−1^ and only 0.055% decay per cycle at 5.0 C during 1000 cycles together with a high areal capacity of 2.20 mAh cm^−2^ under 4.61 mg cm^−2^ sulfur loading even after 200 cycles at 0.2 C. This strategy highlights a new perspective for achieving high‐energy‐density Li‐S batteries.

## Graphical abstract

Herein, highly active and stable cathode system of nitrogen‐doped porous carbon nanotubes/Ni_x_Co_1‐x_S_2_ electrocatalysts is designed. The nitrogen‐doped carbon matrix can alleviate the volume changes and promote the adsorption of LiPSs on chemical binding sites for redox reaction. Moreover, cation‐site‐optimized Ni_Oh_^2+^−S−Co_Oh_^2+^ bonding states in the Ni_x_Co_1‐x_S_2_ can interact moderately with LiPSs that induces weaker sulfur bond strength for sustainable cooperative catalysis.

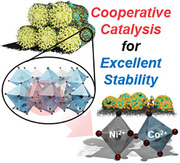

## 1 Introduction

Li‐S batteries have drawn the great interests due to their very high energy storage density (ca. 2600 Wh kg^−1^) with naturally‐abundant and inexpensive sulfur cathode,^[^ ^1^, ^2^, ^3^, ^4^ ^]^ but their practical applications are severely hindered by several technological issues such as low electrical conductivities of sulfur and its discharge products (e.g., Li_2_S_2_, Li_2_S), shuttle effect of polysulfides in the electrolyte, and volume expansion of electrode.^[^ ^5^, ^6^, ^7^ ^]^ Recently, various strategies have been suggested and tried to enhance redox kinetics of cathode reaction and maximize sulfur utilization, which are mostly focused on novel design and application of host cathodes,^[^ ^8^, ^9^ ^]^ functional separators/interlayers,^[^ ^10^, ^11^ ^]^ electrolyte formulations,^[^ ^12^, ^13^ ^]^ binder chemistry,^[^ ^14^, ^15^ ^]^ and selenium/tellurium‐sulfur composites,^[^ ^16^ ^]^ etc. In particular, cathode modification with N‐doping into nonpolar carbonaceous hosts can promote interaction between sulfur species and functional groups retarding the shuttle effect.^[^ ^10^, ^17^, ^18^ ^]^ Meanwhile, common transition metal (TM) compounds in forms of nitrides,^[^ ^19^, ^20^ ^]^ oxides,^[^ ^21^, ^22^ ^]^ chalcogenides,^[^ ^23^, ^24^ ^]^ carbides,^[^ ^25^, ^26^ ^]^ and phosphides^[^ ^27^, ^28^ ^]^ have been introduced to develop sulfur electrochemistry efficiently via adsorption of such metal‐based catalysts on carbon‐based host materials, which can provide abundant pores for sulfur encapsulation, strengthen anchoring property of polysulfides on the surface, and enhance corrosion resistance of catalytic materials.^[^ ^29^ ^]^

Recently, transition metal (TM) sulfides have attracted extensive attention since they have relatively high electrical conductivities and effective binding sites for the lithium polysulfides (LiPSs) on catalyst surface due to high density of valence electrons derived from soft basic S^2−^/S_2_^2−^ anions than those from hard basic O^2−^ ions.^[^ ^30^ ^]^ Among these, a few TM sulfides of pyrite‐type cubic crystals, such as NiS_2_ and CoS_2_, are employed as the effective electrocatalysts because they can accelerate polysulfide redox with much higher conductivity than TM oxides.^[^ ^31^, ^32^ ^]^ Moreover, integrating multi‐cations that reveal individually comparable catalytic role into one composite electrocatalyst could offer an additional means for optimizing the electronic structure and empowering cooperative catalytic effect of TM sulfides.^[^ ^24^ ^]^ Nevertheless, understanding respective roles and contributions of cations in the bimetallic TM sulfides is still barely proceeded to elucidate underlying reasons for enhanced catalytic synergy effect, as compared to the studied catalytic mechanisms of TM oxides.^[^ ^21^, ^33^, ^34^ ^]^

Herein, we present a highly active, long‐term stable, multi‐functional sulfur catalyst system fashioned of Ni_x_Co_1‐x_S_2_ nanocrystals dispersed on the surface of N‐doped porous carbon nanotubes (Ni_x_Co_1‐x_S_2_@NPCTs) as the effective cathode electrocatalyst for rechargeable Li‐S battery. The carbonized porous structure provides a large surface area with buffering space for cyclic redox reactions and a myriad of chemical binding sites with the CNT backbone. Furthermore, designed dual‐active TM sulfides of Ni_Oh_^2+^−S−Co_Oh_^2+^ bonding states in the octahedral TMS_6_ structure are able to effectively catalyze the sulfur cathode reactions with multi‐functional roles; Co_Oh_^2+^ species with vacant S sites in Co−S covalency strongly interact with the adsorbed LiPSs to inhibit the shuttle effect, while occupied Ni_Oh_^2+^ species with optimized doping level properly control the chemical affinity with LiPSs to induce stepwise redox conversion with sustainable cooperative catalysis, leading to alleviation of Li_2_S passivation on the catalyst surface. The Ni_0.261_Co_0.739_S_2_ catalyst on NPCT carbon host outperforms by delivering the highest discharge capacity for the rate capability performance with excellent long‐term cycling stability in the consecutive electrochemical test. This cathode catalyst system is also able to achieve a high performance with favorable cycling results and stability even when the Li‐S battery cell is operated under a harsh condition with low electrolyte to sulfur ratio (E/S = 10.0 µL mg^−1^). Notably, the Ni_0.261_Co_0.739_S_2_@NPCTs catalyst system induces the excellent cyclic durability with a capacity of up to 511 mAh g^−1^ and only 0.055% decay per cycle at fast cycling condition of 5.0 C rate during 1000 cycles together with a high areal capacity of 2.20 mAh cm^−2^ under 4.61 mg cm^−2^ sulfur loading condition even after 200 cycles at 0.2 C. A theoretical DFT calculation also supports the experimental data and strengthens the understandings of the proposed catalyst systems that the sulfur bondings with bond strength and bond length play critical roles in the catalytic performance of Ni_x_Co_1‐x_S_2_@NPCTs with interactions of TM sulfides and lithium polysulfides. Our work could highlight the crucial perspective not only of exploiting the synergy of advanced carbon host with optimized bimetallic sulfide electrocatalysts, but also of shedding light on the obscure atomic configuration relationship for enhanced redox activity of practical Li‐S batteries.

## 2 Results and Discussion

### 2.1 Physicochemical Characterizations of Prepared Catalysts

**Figure 1:**
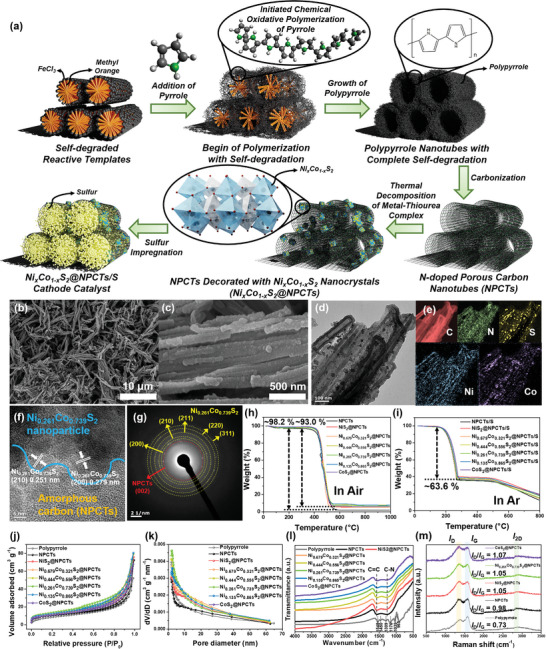
Synthesis and structural characteristics. a) Schematic showing the overall synthesis process using self‐degraded template and thermal decomposition of metal‐thiourea complex methods for the Ni_x_Co_1‐x_S_2_@NPCTs composites. b,c) SEM images with magnified version, d) TEM image, e) EELS mappings, f) HRTEM image with sulfide catalyst highlighted in white lines, and g) SAED pattern of the Ni_0.261_Co_0.739_S_2_@NPCTs cathode catalyst. h,i) TGA analysis curves of the samples at the air and argon conditions, respectively. j,k) N_2_ adsorption/desorption curves and pore size distributions of the samples, respectively. l) FT‐IR spectra and m) Raman spectra of the samples.

**Figure** **1**a illustrates the production steps of Ni_x_Co_1‐x_S_2_@NPCTs composites, where detonations and numbers are based on the actual atomic Ni and Co concentrations determined by inductively coupled plasma atomic emission microscopy (ICP‐AES) and the Ni_x_Co_1‐x_S_2_ samples represent respectively to the catalyst composition of NiS_2_, Ni_0.679_Co_0.321_S_2_, Ni_0.444_Co_0.556_S_2_, Ni_0.261_Co_0.739_S_2_, Ni_0.135_Co_0.865_S_2_, and CoS_2_ (Table S1, Supporting Information). Scanning electron microscope (SEM) images commonly demonstrated that well‐organized 1D carbon nanotube structure was densely grown with length scales ranging up to 8 µm and diameter values up to 200 nm with negligible morphological difference among the samples (Figure 1b,c; Figure S1a‐d, Supporting Information). Energy dispersive spectroscope (EDS) mapping represented homogeneously distributed elements of C, N, S, Ni, and Co, implying the successful formation of Ni_x_Co_1‐x_S_2_ on NPCTs (Figure S1e‐g, Supporting Information). Transmission electron microscope (TEM) images with electron energy loss spectroscope (EELS) mappings also showed that morphology of Ni_x_Co_1‐x_S_2_@NPCTs catalyst was hardly affected as they were uniformly dispersed over entire carbon structures (Figure 1d,e; Figure S2a,b, Supporting Information). High‐resolution TEM (HRTEM) images taken on the NiS_2_@NPCTs, Ni_0.261_Co_0.739_S_2_@NPCTs, and CoS_2_@NPCTs revealed that interplanar distances between adjacent lattice planes are 0.284, 0.279, and 0.278 nm, respectively, corresponding to (200) plane of the TM sulfide catalysts, and are 0.255, 0.251, and 0.249 nm, respectively, being indexed to (210) plane indicated by white lines (Figure 1f; Figure S2a,b, Supporting Information). In addition, selected area electron diffraction (SAED) patterns also confirmed the existence of polycrystalline Ni_x_Co_1‐x_S_2_ electrocatalysts (Figure 1g; Figure S2a,b, Supporting Information). From the thermal gravimetric analysis (TGA) results, the cathode electrode systems of Ni_x_Co_1‐x_S_2_@NPCTs/S appeared to consist of about 1.9 wt.% of Ni_x_Co_1‐x_S_2_ materials, 34.5 wt.% of NPCTs, and 63.6 wt.% of active sulfur, respectively (Figure 1h,i), and the active sulfur was dominantly derived from the melt‐diffusion of elemental sulfur, as supported in the TGA (Figure S3, Supporting Information). As revealed by N_2_ adsorption‐desorption isotherms (Figure 1j,k), the Brunauer–Emmett–Teller (BET) textural values were slightly changed by controlling Ni/Co ratio with the highest surface area of 48.5 m^2^ g^−1^ for the Ni_0.261_Co_0.739_S_2_@NPCTs, and the other composites have surface areas in the range of 39.1 – 44.4 m^2^ g^−1^, pore volumes of 0.096 – 0.115 cm^3^ g^−1^, and pore diameters of 13.2 – 15.1 nm (Table S2, Supporting Information). These values were dramatically decreased after melt‐diffusion of sulfur to prepare active materials of Ni_x_Co_1‐x_S_2_@NPCTs/S, suggesting successful infiltration of sulfur into host materials (Figure S4a,b, Supporting Information). Moreover, carbonization process of polypyrrole increased BET values with newly formed infrared characteristic bands due to stretching vibration of C = C bonds at 1580 cm^−1^ and C‐N bonds at 1260 cm^−1^ in the Fourier transform infrared (FT‐IR) spectra (Figure 1j‐l). Nickel‐ and cobalt‐thiourea complex precursors also showed distinct stretching and rocking frequency absorption bands (Figure S5, Supporting Information).^[^ ^35^ ^]^ After thermal decomposition of those precursors, the NiS_2_@NPCTs, Ni_0.261_Co_0.739_S_2_@NPCTs, and CoS_2_@NPCTs exhibited the higher D band to G band intensity ratios (*I*_D_/*I*_G_) (1.05 – 1.07) than those of polypyrrole (0.73) and NPCTs (0.98) (Figure 1m), revealing formation of defect sites with the relatively larger amounts from the introduction of carbonized host surface and amorphous Ni_x_Co_1‐x_S_2_ which could better contribute to the sulfur redox reactions.^[^ ^36^ ^]^

**Figure 2:**
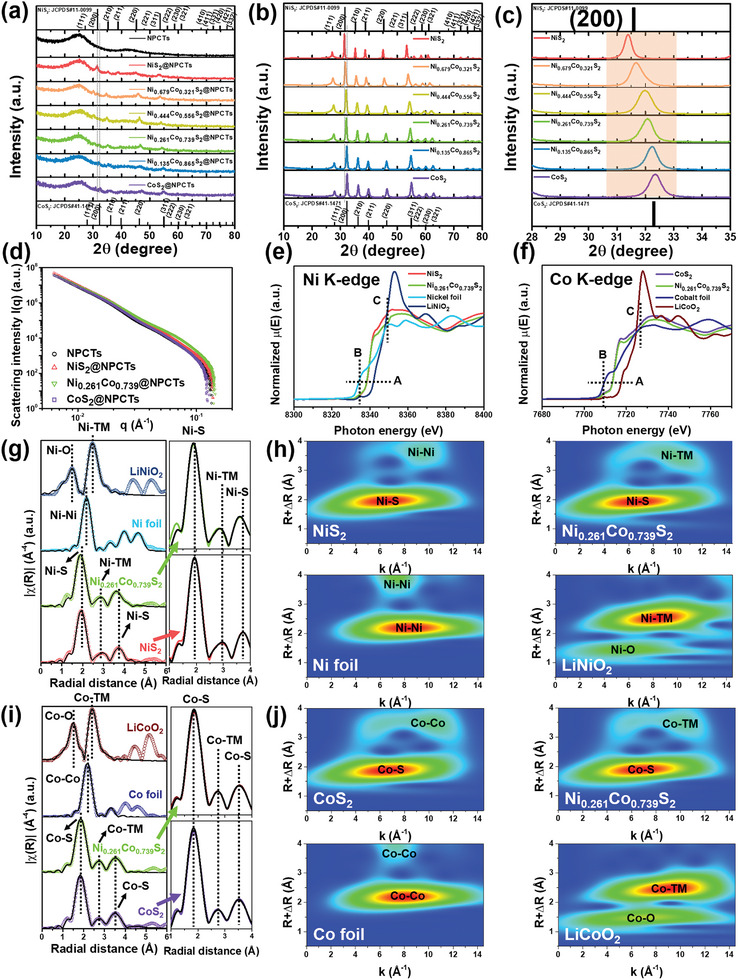
XRD patterns of a) the cathode catalyst composites and b,c) the Ni_x_Co_1‐x_S_2_ electrocatalyst alone without N‐doped carbon supports and their magnified spectra of main peaks indexed to (200) plane. d) SAXS curves of the NPCTs, NiS_2_@NPCTs, Ni_0.261_Co_0.739_S_2_@NPCTs, and CoS_2_@NPCTs. XANES characterizations of powder samples with e) Ni K‐edge XANES spectra of NiS_2_, Ni_0.261_Co_0.739_S_2_, Ni foil, and LiNiO_2_, and f) Co K‐edge XANES spectra of CoS_2_, Ni_0.261_Co_0.739_S_2_, Co foil, and LiCoO_2_. And corresponding *k* ^3^‐weighted FT‐ and WT‐EXAFS spectra with their fitting curves of the g,h) NiS_2_, Ni_0.261_Co_0.739_S_2_, Ni foil, and LiNiO_2_ at Ni K‐edge and i,j) CoS_2_, Ni_0.261_Co_0.739_S_2_, Co foil, and LiCoO_2_ at Co K‐edge.

The X‐ray diffraction (XRD) spectrum of each Ni_x_Co_1‐x_S_2_ catalysts showed diffraction peaks consistent with JCPDS references of NiS_2_ and CoS_2_ with/without carbon supports (**Figure** **2**a,b). Notably, main peaks at ≈2θ of 31 – 32° indexed to (200) plane of Ni_x_Co_1‐x_S_2_ were gradually shifted to high diffraction angle with increased cobalt ratio (Figure 2c), and estimated d‐spacing values via Bragg equation of space group *Pa* $\bar{3}$ for Ni_x_Co_1‐x_S_2_ catalysts are 2.85, 2.82, 2.80, 2.79, 2.78, and 2.76 Å, respectively (Table S3, Supporting Information), which are well‐matched with the HRTEM information (Figure 1f; Figure S2a,b, Supporting Information). Rietveld refinement results calculated from high resolution powder diffraction (HRPD) patterns (Figure S6a‐d, Supporting Information) also produce similar d‐spacing values about 2.82, 2.80, 2.79, and 2.77 Å for Ni_0.679_Co_0.321_S_2_, Ni_0.444_Co_0.556_S_2_, Ni_0.261_Co_0.739_S_2_, and Ni_0.135_Co_0.865_S_2_, respectively (Table S4, Supporting Information). These results are in line with the values obtained by measuring the unit cell size and determining the lattice parameters of NiS_2_, Ni_0.25_Co_0.75_S_2_, and CoS_2_ through computational methods, where a 2 × 2 × 2 cell was used to achieve the Ni:Co ratio of 0.25:0.75 for Ni_0.261_Co_0.739_S_2_. The lattice parameters of relaxed bulk models were 5.66 Å for NiS_2_, 5.59 Å for Ni_0.25_Co_0.75_S_2_, and 5.55 Å for CoS_2_ (Figure S7a and Table S5, Supporting Information), and also revealed a peak shift in the same direction with experimental XRD (Figure S7b, Supporting Information). The X‐ray photoelectron spectroscopy (XPS) survey spectra showed five characteristic peaks of C 1s, N 1s, S 2p, Ni 2p, and Co 2p (Figures S8a and S9a,e,j, Supporting Information). The observed main C–C/C = C and C–N bonds of C 1s spectra of all composites reveal the formation of N‐doped carbon matrix with no substantial surface variations (Figures S8b and S9b,f,k, Supporting Information).^[^ ^37^ ^]^ The N 1s spectra represent oxidized‐N, graphitic‐N, pyrrolic‐N, and pyridinic‐N with total nitrogen contents of ca. 10.34, 10.22, 10.46, and 10.84 at.% for the NPCTs, NiS_2_@NPCTs, Ni_0.261_Co_0.739_S_2_@NPCTs, and CoS_2_@NPCTs, respectively; such similar level of N contents with respect to the samples may imply that thermal decomposition of metal‐thiourea complexes would be reliable process for introduction of nitrogen species to the electrocatalysts (Figures S8c and S9c,g,l, Supporting Information). Specifically, the dominantly formed graphitic‐N could enhance electrical conductivity of the electrocatalyst, and the pyrrolic‐N and pyridinic‐N species are known to Lewis base that can effectively interact with lithium sulfides via lone‐pair electrons to immobilize and convert LiPSs.^[^ ^38^, ^39^ ^]^ TM 2p and S 2p XPS spectra (Figures S8d‐f and S9h,i,m.n, Supporting Information) showed distinct TM^2+^ peaks of TM 2p_1/2_/2p_3/2_ and 2p_1/2_/2p_3/2_ orbitals of sulfide, respectively, suggesting that both Ni and Co elements represent the cubic crystals of Ni_x_Co_1‐x_S_2_ phase.^[^ ^40^ ^]^ Ex‐situ small‐angle X‐ray scattering (SAXS) analysis was conducted (Figure 2d), and core‐shell cylinder model fitting was applied to identify the structural characterization of NPCTs, NiS_2_@NPCTs, Ni_0.261_Co_0.739_S_2_@NPCTs, and CoS_2_@NPCTs (Figure S10, Supporting Information). The fitted parameters obtained from model fitting showed radius, thickness, and length values of ca. 968.27 Å, 357.13 Å, and 76 570 Å, respectively, without significant variations (Table S6, Supporting Information), which is well‐consistent with the observed length scales of Ni_x_Co_1‐x_S_2_@NPCTs catalysts from SEM and TEM images (see Figure 1b‐d; Figures S1a‐d and S2a,b, Supporting Information). In addition, we have verified the local structural properties of Ni_x_Co_1‐x_S_2_ using synchrotron‐based X‐ray absorption near edge structure spectroscopy (XANES) and extended X‐ray absorption fine structure spectroscopy (EXAFS). The normalized Ni K‐edge XANES spectra of NiS_2_ and Ni_0.261_Co_0.739_S_2_ are displayed in Figure 2e. The near‐edge absorption positions (line A) were located between nickel foil (Ni^0^) and LiNiO_2_ (Ni^3+^), indicating that Ni has an oxidation state between 0 and +3 which is well matched with the previous Ni 2p XPS results (Figure S8d, Supporting Information), and no obvious change of Ni oxidation state was observed. Due to the dipole‐forbidden transition of electrons from 1s to 3d of coordinated Ni^2+^, both of NiS_2_ and Ni_0.261_Co_0.739_S_2_ spectra showed slightly notable pre‐edge features at the dashed line B, which specifies distorted octahedral coordination of Ni‐S bonds.^[^ ^41^ ^]^ Notably, the absorption spectra (line C), assigned to charge transitions from 1s to Ni 4p mixed with S 3p states, indicate that the Ni_0.261_Co_0.739_S_2_ has lower intensity than that of NiS_2_, revealing a relatively weak interaction of Ni‐S covalency of Ni_0.261_Co_0.739_S_2_ with more active sites which might result in low overpotential of LiPSs conversion.^[^ ^42^, ^43^ ^]^ Likewise, Co K‐edge XANES spectra (Figure 2f) showed that Co oxidation state also has values between 0 and +3. The *k* ^3^‐weighted Fourier‐transformed (FT) spectra were presented (Figure 2g,i) with fitted coordination number, Debye‐Waller factor (σ^2^), bond length, and R‐factor on each chemical bonds (Tables S7 and S8, Supporting Information). For Ni K‐edge (Figure 2g), the spectra of NiS_2_ and Ni_0.261_Co_0.739_S_2_ showed distinct Ni‐S and Ni‐TM (TM = Ni, Co) bond peaks, which have totally different locations from Ni‐Ni bond of nickel foil and Ni‐O/Ni‐TM bonds of LiNiO_2_, referring well‐synthesized Ni_x_Co_1‐x_S_2_ catalysts without any oxygen bond impurities. The calculated bond lengths for Ni_0.261_Co_0.739_S_2_ are ca. 2.358, 3.445, and 3.918 Å for Ni‐S (1st), Ni‐S (2nd), and Ni‐TM, respectively, which are relatively shorter than those of NiS_2_ with ca. 2.391, 3.502, and 4.037 Å for Ni‐S (1st), Ni‐S (2nd), and Ni‐Ni in Figure 2g, in a good agreement with HRTEM patterns and calculated lattice information (see Figure 1f; Figure S2a,b and Tables S3–S5, Supporting Information). The higher values of overall Debye‐Waller factors of Ni_0.261_Co_0.739_S_2_ indicate more disordered lattice environment than that of NiS_2_.^[^ ^24^, ^44^ ^]^ Wavelet‐transformed (WT) EXAFS spectra of NiS_2_ and Ni_0.261_Co_0.739_S_2_ presented distinctive maximum contour peaks that are not overlapped with any peaks in nickel foil and LiNiO_2_ (Figure 2h), revalidating clear formation of Ni_x_Co_1‐x_S_2_ phase with Ni‐S covalency. Specific spectra of CoS_2_ and Ni_0.261_Co_0.739_S_2_ at Co K‐edge also manifest the existence of distinct Co‐S and Co‐TM bonds, and the increased values of Debye‐Waller factor and bond lengths of Ni_0.261_Co_0.739_S_2_ compared to those of CoS_2_ demonstrate that the distorted structures with long covalent bonds are occurred (Figure 2i). The distinctive maximum WT contour peaks elucidate the distinguishable Co‐S covalency in Ni_x_Co_1‐x_S_2_ crystal structures (Figure 2j).

### 2.2 Electrochemical Li‐S Cell Performances of the Prepared Electrocatalysts

The adsorption feature of LiPSs on the electrocatalyst layer should be considered as a critical factor for enhancing redox kinetics of Li‐S batteries. Therefore, we have conducted two‐type Li_2_S_6_ adsorption tests in order for examination of affinity properties of (i) catalyst‐dispersed carbon hosts and (ii) Ni_x_Co_1‐x_S_2_ catalysts without carbon supports. The slightly decolorized electrolyte containing NPCTs visually demonstrates some immobilization effect driven by the polar N‐doped matrix (Figure S11a, Supporting Information). Furthermore, all of electrolytes appeared to be transparent after introducing the Ni_x_Co_1‐x_S_2_ catalysts, revealing an efficient and good performance of Ni_x_Co_1‐x_S_2_ for Li_2_S_6_ adsorption capacity. From the UV‐Vis absorption spectra of the Ni_x_Co_1‐x_S_2_@NPCTs catalyst revealed a decreased peak intensity at 365 nm that is associated with S_6_^2−^ ions (Figure S11b, Supporting Information). Moreover, a higher degree of transparency was achieved as the cobalt content of Ni_x_Co_1‐x_S_2_ is larger; the most diminished UV‐Vis peak intensity was observed for the CoS_2_ sample (Figure S11c,d, Supporting Information). Consequently, it is demonstrated that the precise regulation of the LiPSs adsorption property on the electrocatalysts is possible via partial replacement of cationic sites, which should be taken into consideration for the Li‐S battery cathode reactions.

**Figure 3:**
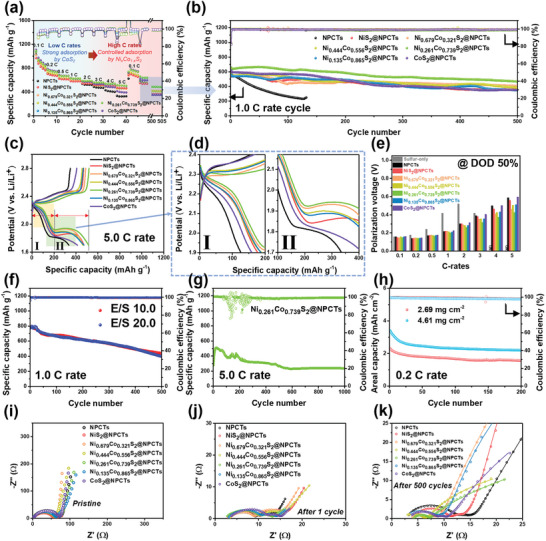
Electrochemical performances of NPCTs, and Ni_x_Co_1‐x_S_2_@NPCTs cathode catalysts for Li‐S batteries. a) Rate capability performance, b) cycling performance at 1.0 C, and c,d) galvanostatic charge‐discharge profiles at 5.0 C with enlarged areas of the first discharge plateau and beginning of the charging process (marked as I), and the second discharge plateau (marked as II). e) Polarization voltage at 50% DOD for different C‐rates. (*N/D: Polarization values are not determined for a comparison). Various cycling performances of Ni_0.261_Co_0.739_S_2_@NPCTs with f) low E/S ratio of 10.0 (and 20.0), g) 5.0 C rate, and h) high sulfur loadings of 4.61 mg cm^−2^ (and moderate loading of 2.69 mg cm^−2^) conditions. Ex‐situ EIS analysis of the samples recorded at i) pristine state, j) 1‐cycled state, and k) 500‐cycled state. Experimental cycling condition before the EIS analysis was maintained at a 1.0 C rate.

To investigate the electrocatalytic effects derived from different LiPSs interaction on the adsorption layer of the cathode catalysts, the rate capability and cycling performance of the electrocatalysts are presented (**Figure** **3**a,b; Figure S12, Supporting Information). Sulfur‐only cathode demonstrated the lowest discharge capacity, emphasizing the role and effect of carbon support matrix for stable sulfur impregnation, and the NPCTs host delivered a highly improved but still low discharge capacity. The catalyst of CoS_2_@NPCTs exhibited a higher discharge capacity than the NiS_2_@NPCTs at low C rates (0.1–3.0 C), which is possibly attributed to suppressed shuttling effect via a stronger interaction with LiPSs and thereby longer holding time for stepwise conversion reaction (Figure 3a). However, too much strong interaction via excessive LiPSs adsorption could rather inhibit complete sulfur redox cycles for a typical Li‐S battery, therefore its binding energy should be modulated to enable fast electron transfer and overcome limit in catalyzing functions.^[^ ^22^, ^45^ ^]^ Through tuning the cationic configuration in the cubic TM sulfides of Ni_x_Co_1‐x_S_2_, the Ni_0.261_Co_0.739_S_2_@NPCTs showed the highest specific capacity attributed to optimal adsorption of LiPSs on the catalytic layer, and the electrochemical tests were performed and compared with Ni_x_Co_1‐x_S_2_ without carbon hosts and active sulfur (Figure S13a,b, Supporting Information). Subsequently, we conducted a synchrotron‐based operando XRD test to directly monitor the polysulfide conversion during cycling (Figure S14, Supporting Information). At the first cycle obtained at 1.0 C rate as marked as (a1)‐(f1), the TM sulfide of Ni_0.261_Co_0.739_S_2_ reveals the formation of Li_2_S_x_ (ca. 25.5°) and Li_2_S (ca. 27.1°), and transition from *α*‐S_8_ (222) to *β*‐S_8_ (130) as the lithiation occurs. Once the delithiation has started, Li_2_S oxidation can be observed with gradually detected *α*‐S_8_ peak.^[^ ^46^, ^47^ ^]^ For the second discharge at 0.05 C marked as (a2)‐(f2), Ni_0.261_Co_0.739_S_2_ also shows diminished *α*‐S_8_ peak, and generated polysulfide and *β*‐S_8_ signals apparently. Thus, all of these phase transformations are in a good agreement with electrochemical behaviors with improved conversion efficiency, while the control group seems to suffer from full polysulfide utilization. Furthermore, cycling performances at 1.0 C represented typical volcano plots with the best performance for the Ni_0.261_Co_0.739_S_2_@NPCTs, demonstrating the highest cycle stability of 72.8% retention with 0.054% capacity decay per cycle (Figure 3b; Figure S15, Supporting Information). NiS_2_@NPCTs showed 66.5% retention and capacity loss ratio of 0.067% per cycle, and the Co_Oh_^2+^‐engineered cathodes of Ni_0.679_Co_0.321_S_2_@NPCTs and Ni_0.444_Co_0.556_S_2_@NPCTs exhibited 69.0% and 69.5% with 0.062% and 0.061% decay rate, respectively, suggesting that the catalytic Ni_x_Co_1‐x_S_2_ can function as the effective LiPSs immobilizer to minimize the shuttling effect. Subsequently, the enhanced kinetics over the cathode catalyst of Ni_0.261_Co_0.739_S_2_@NPCTs could be verified by the galvanostatic charge‐discharge profiles obtained at different current rates (Figure 3c,d; Figure S16, Supporting Information). During cycling, series conversion reactions can be clearly observed as two typical discharge plateaus which are reflected in yellow and green rectangular marks: (I) the first plateau of S_8_ (solid) → Li_2_S_4_ (liquid), and (II) the second plateau of Li_2_S_4_ (liquid) → Li_2_S (solid). At low current rates of discharging, the CoS_2_@NPCTs showed low overpotentials in the Li_2_S_2_/Li_2_S deposition with high charge resistances during solid‐liquid reaction. Meanwhile, the NiS_2_@NPCTs showed low overpotentials and resistances during the fast phase transformations, indicating that the alleviated binding energy strength could be advantageous for rapid conversion. Therefore, the dual component system of nickel and cobalt sulfides of Ni_0.261_Co_0.739_S_2_@NPCTs cathode catalyst is able to provide overall low overpotential values, demonstrating that the facilitated sulfur redox is achieved by an optimally tuned adsorption features leading to the efficient conversion process. As a result, although the proportions of second plateau are gradually reduced with increasing current rate due to the insufficient reduction of Li_2_S_4_, that of Ni_0.261_Co_0.739_S_2_@NPCTs in the region II occupies 60.4% at a significantly high current rate of 5.0 C with an improved sulfur utilization (Figures S17 and S18, Supporting Information). Complementarily, the galvanostatic discharge profiles during 1.0 C cycling performances of Ni_0.261_Co_0.739_S_2_@NPCTs demonstrated the lowest overall overpotentials with greater sulfur utilization in the respective reactions, and dramatically increased voltage differences between the Ni_0.261_Co_0.739_S_2_@NPCTs and other samples as the long battery operation proceeded (Figure S19, Supporting Information). Additionally, the values of voltage hysteresis differences at 50% depth of discharge (DOD) were investigated, which also represented the volcano plots indicating that the Li‐S cell made of Ni_0.261_Co_0.739_S_2_@NPCTs can exhibit the lowest polarization voltage with a minimal energy loss regardless of different C rates (Figure 3e). Besides, we investigated long‐term cycling performances at 1.0 C rate under the conditions of E/S = 10.0 and 20.0 for the Ni_0.261_Co_0.739_S_2_@NPCTs (Figure 3f). Regardless of the decreased contents of electrolyte, the Ni_0.261_Co_0.739_S_2_@NPCTs cathode exhibited similar cycling retentions, especially to the initial discharge capacity of 791 mAh g^−1^ with 0.088% decay per cycle for E/S = 10.0, indicating the potential for further exploitation in industrial field with appropriate cell design of low E/S ratio.^[^ ^48^ ^]^ In addition, the Ni_0.261_Co_0.739_S_2_@NPCTs cell showed a capacity up to 511 mAh g^−1^ and the final capacity of ca. 236 mAh g^−1^ even after 1000 cycles under the fast cycling at 5.0 C rate with only 0.055% decay per cycle (Figure 3g). This achievement is comparable with long‐term cycling performances of metal sulfide host cathodes proposed in the previous literatures (Figure S20 and Table S9, Supporting Information). For the Ni_0.261_Co_0.739_S_2_@NPCTs cell with a high sulfur loading of 4.61 mg cm^−2^, to satisfy a demand from industrially accepted level above 4 mg cm^−2^,^[^ ^49^ ^]^ a high areal capacity of 2.20 mAh cm^−2^ was achieved even after 200 cycles at 0.2 C rate, which exhibits relatively stable cycling performance compared to that of moderate sulfur loading of 2.69 mg cm^−2^ showing 1.56 mAh cm^−2^ with same cycle conditions (Figure 3h). The same cell showed expected gravimetric energy density values from 449.2 to 673.5 mWh g^−1^ during subsequent 200 cycles which were calculated from galvanostatic charge‐discharge profiles, and these values could fulfill commercially needed conditions (above 400 mWh g^−1^) (Figure S21 and Table S10, Supporting Information).^[^ ^50^ ^]^ We have investigated ex‐situ Nyquist plots of electrochemical impedance spectroscopy (EIS) after 1.0 C cycling processes for the cathodes in pristine, 1 cycled, and 500 cycled states (Figure 3i‐k), and resultant fitting parameters are summarized in Table S11 (Supporting Information) with fitted equivalent circuit diagrams (Figure S22, Supporting Information). The obtained *R*_0_ values of 2.45 Ω (± 0.45 Ω) were almost similar owing to the same electrolyte environment. All the pristine cells exhibited only one semicircle attributed to the charge transfer resistance *R*_1_, showing no significant change after introducing electrocatalysts because charge migration at the electrolyte/electrode interface was not obstructed. After the first discharge‐charge cycle, *R*_1_ values were considerably decreased, which could confirm the formation of effective solid electrolyte interphase (SEI) inducing better charge transportation between electrolyte and electrodes. Furthermore, the Li‐S cells showed additional semicircles corresponding to interfacial contact resistances of *R*_2_ and *R*_3_, representing SEI formation and Li^+^ diffusion into the active mass, respectively.^[^ ^51^, ^52^, ^53^ ^]^ Specifically, the Ni_0.261_Co_0.739_S_2_@NPCTs cathode catalyst showed low values of *R*_2_ (2.2 Ω) and *R*_3_ (19.2 Ω) in a charged state, which might be ascribed to catalytic conversion of dissolved polysulfide inducing thin SEI layer and restraining undesirable deposition of Li_2_S_2_/Li_2_S on the anode surface for favorable Li^+^ transport. Interestingly, the CoS_2_@NPCTs showed much lower *R*_3_ value than that of NiS_2_@NPCTs, which may signify that mechanism of Li^+^ flux and polysulfide deposition toward Li anode could undergo improvements with the strong LiPSs immobilizer. When the Li‐S battery cells were performed for 500 cycles, charge transfer resistance *R*_1_ of Ni_0.261_Co_0.739_S_2_@NPCTs notably decreased, representing a good electrolyte wettability for the cathode substrate even after such long cycle operation (Figure 3k). In particular, it remarkably showed the lowest values of *R*_2_ (2.3 Ω) and *R*_3_ (42.3 Ω), further revealing reduced stacking of LiPSs on the electrode surface. Again, it is also ascertaining the importance of introducing dual‐active sites that can facilitate LiPSs redox to suppress surface passivation from diffused/soluble polysulfide.

### 2.3 Electrochemical Sulfur Redox Reactions over the Prepared Electrocatalysts

**Figure 4:**
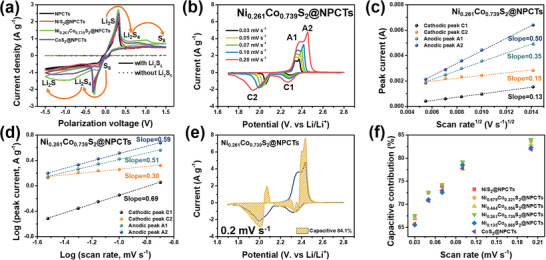
CV curves of a) the symmetric cells at 0.10 mV s^−1^ for NPCTs, NiS_2_@NPCTs, Ni_0.261_Co_0.739_S_2_@NPCTs, and CoS_2_@NPCTs cells, and b) the asymmetric cell fabricated with Ni_0.261_Co_0.739_S_2_@NPCTs at various rates of 0.03 – 0.20 mV s^−1^ with cathodic/anodic redox peaks specified as C1, C2, A1, and A2, respectively. The fitted lines with linear relationship c) between cathodic/anodic redox peak currents versus the square root of the scan rate and d) between log scale of peak currents versus that of scan rate plots at reduction and oxidation processes for Ni_0.261_Co_0.739_S_2_@NPCTs cathode. e) CV curve with a fraction of capacitive charge storage denoted by yellow region at 0.2 mV s^−1^ and f) different capacitive contributions at various scan rates for Ni_x_Co_1‐x_S_2_@NPCTs cathode catalysts.

To further research the contribution of Ni_x_Co_1‐x_S_2_ electrocatalysts to the polysulfide conversion, we have applied cyclic voltammetry (CV) techniques over symmetrical cells with/without Li_2_S_6_ (**Figure** **4**a). The polarization profiles can verify minor contribution of capacitive current as indicated by the Li_2_S_6_‐free cells (dotted lines). After mixing Li_2_S_6_ with the electrolyte, all the cells showed increased redox current response, implying that the polysulfide reactions are the major fraction instead of double‐layer capacitance.^[^ ^54^ ^]^ In detail, the smallest distinct peak shifts located at −0.315 V and 0.315 V, and the highest currents for the Ni_0.261_Co_0.739_S_2_@NPCTs demonstrate its improved conversion kinetics. Subsequently, CV analysis over the asymmetrical cells at various sweep rates from 0.03 to 0.20 mV s^−1^ was also conducted (Figure 4b; Figure S23, Supporting Information). The voltammogram profile provides the distinct peaks: cathodic peaks as C1 [S_8_ (solid) → Li_2_S_x_ (liquid) (4 ≤ x ≤ 8)], C2 [Li_2_S_x_ (liquid) (2 ≤ x ≤ 4) → Li_2_S (solid)] and anodic peaks as A1 [Li_2_S (solid) → Li_2_S_x_ (liquid) (2 ≤ x ≤ 4)], A2 [Li_2_S_x_ (liquid) (4 ≤ x ≤ 8) → S_8_ (solid)].^[^ ^55^ ^]^ Using Randles‐Sevcik equation (Equation S7, Supporting Information), we fitted the peak currents of each points to show a linear tendency for Li^+^ mobility (Figure 4c; Figure S24a–e, Supporting Information). To be specific, the Ni_0.261_Co_0.739_S_2_@NPCTs showed remarkable ion mobility values at C1, C2, A1, and A2 showing ca. 9.26 x 10^−9^ cm^2^ s^−1^, 1.28 x 10^−8^ cm^2^ s^−1^, 6.77 x 10^−8^ cm^2^ s^−1^, and 1.36 x 10^−7^ cm^2^ s^−1^, respectively, indicating cooperation of catalytic sites has a great influence on mass transfer of Li^+^ toward the electrochemical interface (Figure S24f,g, Supporting Information). In addition, plots of differential capacity (dQ/dV) versus potential (V) reproduced from the first discharge‐charge profiles displayed similar tendency compared to the previous CV profiles providing overall low overpotential of the Ni_0.261_Co_0.739_S_2_@NPCTs in every redox region (Figure S25, Supporting Information). The capacitive‐contributed current can be obtained via Equations S8 and S9 (Supporting Information) (Figure 4d; Figure S26, Supporting Information), and corresponding shaded area is expressed within CV curve (Figure 4e; Figures S27–S32, Supporting Information).^[^ ^56^, ^57^ ^]^ The fractions in their current values of all Ni_x_Co_1‐x_S_2_@NPCTs catalysts are gradually dominated by capacitive‐controlled contribution as the scan rate increases, because the sluggish Li^+^ diffusion could not exert adequate influence on charge‐storage process at high current density,^[^ ^56^, ^57^ ^]^ and the capacitive ratios of the Ni_0.261_Co_0.739_S_2_@NPCTs were larger than those of other cathodes at all scan rates (Figure 4f). This observation clearly indicates that one of the reasons for a high electrocatalytic activation of the Ni_0.261_Co_0.739_S_2_@NPCTs is due to large number of active sites to encourage Li^+^ storage with the highest specific surface area and regulated affinity to derive 3D Li_2_S formation with facilitated ion transfer. For further clarity on the functional roles of electrocatalysts, we compared ex‐situ SEM observations of Li and cathode surfaces tested after 500 cycles at 1.0 C rate as displayed in Figure S33 (Supporting Information) (for the NPCTs, it was tested only for 150 cycles due to its stability failure). The NPCTs exhibited a very unstable Li plating behavior with polysulfide residues, which means the use of the carbon matrix alone is insufficient to suppress accumulation of dendritic Li and polysulfide diffusion toward anode (Figure S33a,e, Supporting Information). After introducing the TM sulfide electrocatalysts, however, for examples, the NiS_2_@NPCTs showed relatively even distribution of Li with sharply bumpy surface (Figure S33b,f, Supporting Information), and the CoS_2_@NPCTs also demonstrated uniform Li deposition in island‐like morphologies with relatively flat surfaces (Figure S33d,h, Supporting Information). Especially, the Ni_0.261_Co_0.739_S_2_@NPCTs provided rather flat and dense Li deposition without any dendrite formation visualized by the cleanest Li image without identifiable cracks (Figure S33c,g, Supporting Information), which is well consistent with the previous EIS measurements (see Figure 3i‐k). Moreover, the support of NPCTs showed agglomerated active materials in an irregular morphology after cycling process (Figure S33i,m, Supporting Information). Notably, the Ni_0.261_Co_0.739_S_2_@NPCTs cathode catalyst represented 3D deposition type which should be favorable for overcoming the sluggish conversion kinetics (Figure S33k,o, Supporting Information). These assessments imply that significantly enhanced reversible redox and cycling stability are achieved by repressing inhomogeneous Li^+^ flux and maintaining the 3D nucleation mechanism even with long cycling performances. Ex‐situ SAXS results on the Ni_0.261_Co_0.739_S_2_ catalyst samples collected from electrodes also showed stable maintenance of carbon backbone structures after long‐term cycling tests including the normal‐loading conditions (1.5 mg cm^−2^) of 500 cycles at 1.0 C, 1000 cycles at 5.0 C, and the moderate‐ and high‐loading conditions (2.69 and 4.61 mg cm^−2^) of 200 cycles at 0.2 C compared to the pristine states (Figure S34, Supporting Information). Fitting parameters from Guinier‐Porod model showed the increased radius of gyration values, which might be ascribed to adsorbed residues on overall components of active material, conductive agent, and binder during long‐term battery operation (Table S12, Supporting Information). Moreover, ex‐situ XRD analysis, which was conducted after long‐term 500 cycles at 1.0 C rate for the NiS_2_@NPCTs/S, Ni_0.261_Co_0.739_S_2_@NPCTs/S, CoS_2_@NPCTs/S, NiS_2_/S, Ni_0.261_Co_0.739_S_2_/S, and CoS_2_/S electrodes, showed that the crystallinity features of the Ni_x_Co_1‐x_S_2_ catalysts were sustained without critical degradation (Figure S35, Supporting Information). In other words, all of results of the ex‐situ experiments indicated that the Li_2_S nucleation mechanism could be preserved for long cycling processes due to the maintained carbon matrix structure and the crystallinity of the electrocatalyst.

### 2.4 Discussion on Underlying Mechanisms for Enhanced Electrocatalytic Performance

We presented the highly active and stable Ni_x_Co_1‐x_S_2_@NPCTs electrocatalysts which were systematically fabricated and applied as the cathode system of Li‐S batteries for the favorable adsorption and conversion of LiPSs, as described in the Results section. Most of all, the Li‐S cell with the Ni_0.261_Co_0.739_S_2_@NPCTs catalyst showed an excellent cyclic stability with a very small capacity decay of only 0.055% or less per cycle even at a high rate of 5.0 C during 1000 cycles. The rationale for such high electrocatalytic performance of the proposed material can be understood by several important factors as will be discussed below.

**Figure 5:**
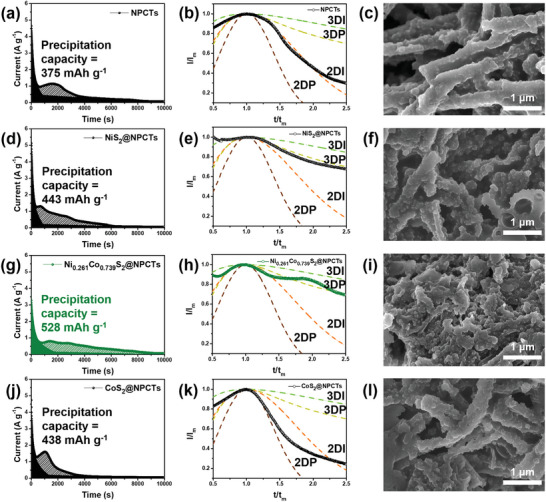
Potentiostatic discharge profiles of Li_2_S_6_ solution at 2.03 V (vs Li/Li^+^) showing Li_2_S precipitation capacity, dimensionless i‐t curves for Li_2_S nucleation mechanism, and corresponding ex‐situ SEM images of the Li_2_S‐grown cathode catalysts for the a–c) NPCTs, d–f) NiS_2_@NPCTs, g–i) Ni_0.261_Co_0.739_S_2_@NPCTs, and j–l) CoS_2_@NPCTs.

The 3D nucleation and growth of Li_2_S with LiPSs over the Ni_0.261_Co_0.739_S_2_@NPCTs could contribute to the highly enhanced electrochemical performance of Li‐S batteries. To validate the effects of different binding energies of the Ni_x_Co_1‐x_S_2_@NPCTs catalysts, we have investigated the Li_2_S nucleation types formed during the Li_2_S_6_ reduction process through chronoamperometric results along with corresponding ex‐situ SEM images of the cathode catalysts (**Figure** **5**a‐l; Figure S36a‐i, Supporting Information) and the four electrochemical deposition models of (1) 2D instantaneous growth (2DI), (2) 2D progressive growth (2DP), (3) 3D instantaneous growth (3DI), and (4) 3D progressive growth (3DP).^[^ ^22^ ^]^ The 2D models imply the merging of adjacent atoms into the lattice interface which induces a planar deposition layer, and the 3D models indicate the 3D volumetric diffusion controlled process (see details in Supporting Information).^[^ ^12^, ^22^ ^]^ As well known, the reaction of Li_2_S_2_ (solid) → Li_2_S (solid) is the rate‐limiting process with the highest energy barrier,^[^ ^58^ ^]^ thus, exploring Li_2_S_2_/Li_2_S precipitation step is important to confirm the catalytic effects along with various diffusion mechanisms depending on type of Ni_x_Co_1‐x_S_2_. The NPCTs showed the lowest Li_2_S precipitation capacity (375 mAh g^−1^) with the growth of relatively planar Li_2_S without any deposited particulates, consistent with dimensionless current‐time curves of 2DI growth (Figure 5a‐c; Table S13, Supporting Information). It is noteworthy that the catalyst system of CoS_2_@NPCTs showed 2DP‐2DI nucleation with the low precipitation capacity of 438 mAh g^−1^ (Figure 5j‐k) which could also encourage full 2D deposition (Figure 5l). Meanwhile, as the NiS_2_@NPCTs with limited active sites and low binding strength could lead to the 3DP type but also low electrodeposition capacity (443 mAh g^−1^) with much shorter deposition time (*t*_m_) than that of other samples, it is reasonable to consider that cationic configuration should be accompanied for diffusion‐favorable Li_2_S nucleation (Figure 5d‐f). Thus, the Ni_0.261_Co_0.739_S_2_@NPCTs catalyst showed the highest nucleation capacity of 528 mAh g^−1^ with favorable 3DI‐3DP growth type and relatively long discharge time (Figure 5g‐i), in a good agreement with the ex‐situ SEM analysis of the cathode catalyst after cycling test showing 3D deposition type (see Figure S33k,o, Supporting Information), strongly suggesting that an optimized cationic ratio of Co_Oh_^2+^ and Ni_Oh_^2+^ should be taken into account for overcoming the sluggish kinetics through balance between immobilization and diffusion‐controlled conversion of LiPSs.

**Figure 6:**
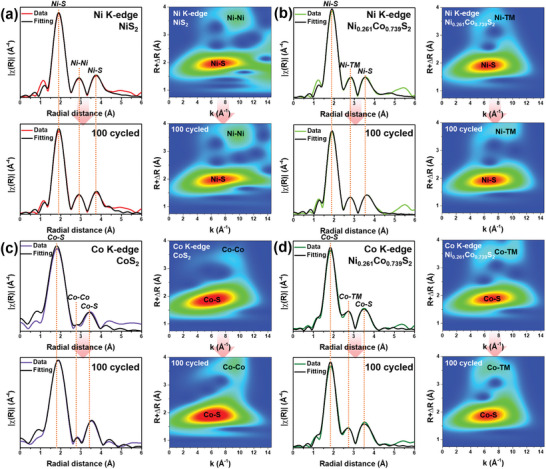
Ex‐situ *k* ^3^‐weighted FT‐ and WT‐EXAFS analysis results with fitting curves for electrode samples in prsitine and 100‐cycled at 1.0 C rate states of a) NiS_2_, b) Ni_0.261_Co_0.739_S_2_ at Ni K‐edge, and c) CoS_2_, d) Ni_0.261_Co_0.739_S_2_ at Co K‐edge.

Dual‐active Ni_Oh_^2+^−S−Co_Oh_^2+^ bonding states should be another reason for the outstanding catalytic activity as they can control the chemical binding energies and induce cooperative redox conversion of LiPSs. To further understand fundamental effects of Ni and Co species on the electrochemical properties, we have analyzed ex‐situ FT and WT *k* ^3^‐weighted EXAFS of the NiS_2_, Ni_0.261_Co_0.739_S_2_, and CoS_2_ cathodes under pristine and 100 cycled conditions by tracking changes in resultant parameters (**Figure** **6**; Tables S14 and S15, Supporting Information). The pristine states of NiS_2_, Ni_0.261_Co_0.739_S_2_ in Ni K‐edge and CoS_2_, Ni_0.261_Co_0.739_S_2_ in Co K‐edge showed similar fitting results compared to those of powder samples, and little differences are probably attributed to slight distortion of electronic structure as in slurry‐casted state consisting of sulfur, CNT, and binder on Al substrate.^[^ ^41^ ^]^ For the Ni K‐edge results, the coordination number values of Ni‐S bond in NiS_6_ octahedron have overall diminishing trend after 100 cycles, as the first coordination shell of Ni_Oh_^2+^ showed change from ca. 5.922 to ca. 5.897 for NiS_2_, and difference became significant for the Ni_0.261_Co_0.739_S_2_ case showing from ca. 5.937 to ca. 5.856. This indicates that Ni‐S bond suffers from degradation due to a participation of Ni_Oh_^2+^ sites in the catalytic conversion, and it becomes severe after partial substituting Ni_Oh_^2+^ by sulfiphilic Co_Oh_^2+^ due to enhanced LiPSs interaction on catalytic surface. Surprisingly, for the Co K‐edge, the coordination number (CN) of Co‐S bond after 100 cycles was increased; for the nearest Co‐S bond of Co_Oh_^2+^ of CoS_2_ displayed a change of CN from ca. 5.874 to ca. 5.972, and a mild change of CN for the Ni_0.261_Co_0.739_S_2_ from ca. 5.817 to ca. 5.897. In other works, although Co_Oh_^2+^ with empty S sites in Co‐S covalency of octahedral CoS_6_ structure of CoS_2_ compound has the catalytic advantage for adsorption and conversion of the LiPSs,^[^ ^59^, ^60^, ^61^ ^]^ it could trap sulfur very strongly during subsequent charge/discharge process that might gradually block catalytic sites, and this phenomenon could be alleviated via partial replacement of Co_Oh_^2+^ by Ni_Oh_^2+^ with moderate affinity and cooperative catalysis of dual‐active Ni_Oh_^2+^−S−Co_Oh_^2+^ backbones of the Ni_0.261_Co_0.739_S_2_ catalyst. The corresponding *k* ^3^‐weighted WT‐EXAFS spectra further provide that the CoS_2_ and Ni_0.261_Co_0.739_S_2_ samples in the Co K‐edge show distinguishable increases of Co‐S bond contributions after cycling test.

Furthermore, a weaker but modulated sulfur bonding strength of Li_2_S_2_ molecule adsorbed on the electrocatalytic Ni_x_Co_1‐x_S_2_@NPCTs surface is another key factor that could also significantly boost the redox kinetics and improve the electrochemical performance of the Li‐S cell. Density functional theory (DFT) calculations were performed on the model surface structures to further reveal the reaction chemistry of the consecutive polysulfides adsorption‐conversion on the Ni_x_Co_1‐x_S_2_ electrocatalyst for subsequent sulfur redox during long term operation. The representative models of NiS_2_, Ni_0.25_Co_0.75_S_2_, and CoS_2_ were designed based on results from the ICP analysis conducted in the experiments and technical reason of QM simulation (Figures S37–S39, Supporting Information). Enlighted by that the conversion from Li_2_S_2_ to Li_2_S is commonly considered as the most crucial step that determines the whole performance,^[^ ^62^, ^63^, ^64^, ^65^ ^]^ the adsorption energy of Li_2_S_2_ molecule on the catalyst surface can play a crucial role in preventing Li_2_S_2_ dissolution from the sulfide cathode, thereby enabling the desired conversion to Li_2_S on the surface. This can be calculated using the following equation:

(1) $$E_{ads}=E_{slab+Li_{2}S_{2}}-\left(E_{slab}+E_{Li_{2}S_{2}}\right)$$

**Figure 7:**
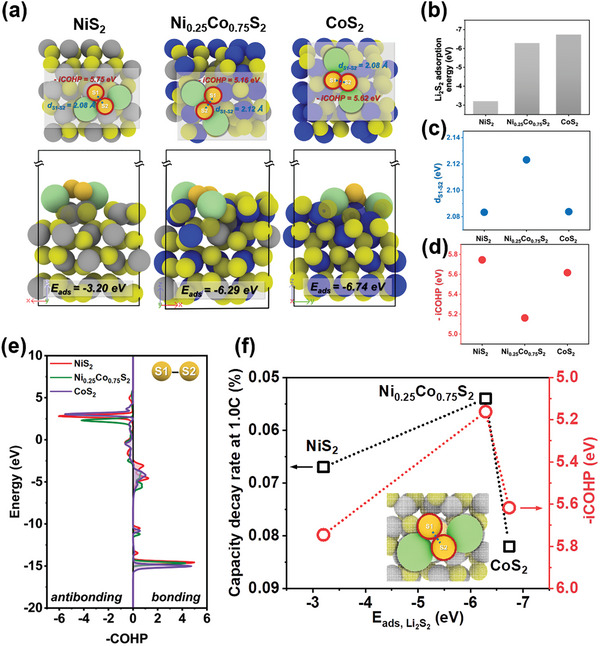
DFT calculation results. a) The Li_2_S_2_ adsorption models of NiS_2_, Ni_0.25_Co_0.75_S_2_, and CoS_2_, the pictures in the first row are of top views, the second row is of side views (Ni: gray, Co: blue, Li: green, S in slab: yellow, S in Li_2_S_2_: dark yellow). b) Adsorption energy of Li_2_S_2_ on each surface. c) Distance of bonding and d) –iCOHP value between sulfur atoms in Li_2_S_2_ molecules adsorbed on each surface. e) COHP analysis of bonding between sulfur atoms in Li_2_S_2_ molecules adsorbed on each surface of the catalysts. f) Volcanot plot for comparison of experimental capacity decay rate at 1.0 C of the electrocatalysts among NiS_2_, Ni_0.25_Co_0.75_S_2_ (Ni_0.261_Co_0.739_S_2_ in the experiments), and CoS_2_ in terms of Li_2_S_2_ adsorption energy and –iCOHP value of S‐S bond in the Li_2_S_2_.

**Figure** **7**a illustrates how the Li_2_S_2_ molecules are adsorbed on the NiS_2_, Ni_0.25_Co_0.75_S_2_, and CoS_2_. The adsorption energies of Li_2_S_2_ on the NiS_2_, Ni_0.25_Co_0.75_S_2_, and CoS_2_ are −3.20 eV, −6.29 eV, and −6.74 eV, respectively (Figure 7b). Therefore, it is reasonable to assert that the better electrochemical performance at low C‐rates of CoS_2_ is induced from the strongest Li_2_S_2_ adsorption on the CoS_2_ surface due to enough time for LiPSs conversion regardless of diffusion mechanism, but it brings diffusion limitation and impedes fast conversion by 2D LiPSs deposition on the catalyst surface. By introducing a small amount of Ni to form Ni_0.25_Co_0.75_S_2_, the adsorption weakens, resulting in 3D‐form deposition of LiPSs and finally enhancing the electrochemical properties during high‐speed charging/discharging. For Li_2_S_2_ to easily convert to Li_2_S, the bond between the sulfur atoms of Li_2_S_2_ on catalytic surfaces could be easily cleaved. Thus, the strength of the S1‐S2 bond was also investigated in adsorbed Li_2_S_2_. The most straightforward indicator is the bond length.^[^ ^55^ ^]^ The S1‐S2 bond length on the NiS_2_ and CoS_2_ is ≈2.08 Å, while on the Ni_0.25_Co_0.75_S_2_, the bond length is 2.12 Å, indicating a weaker bond between sulfur atoms induces enhanced catalytic conversion to Li_2_S (Figure 7c). A crystal orbital Hamilton population (COHP) analysis was employed to figure out more accurate bond strength, which has been established to get the chemical bonding information from electronic structural results, as identifying pairs of atoms from bonding and antibonding interaction.^[^ ^66^ ^]^ In this paper, a minus sign is applied to the COHP values to help understand, as a positive ‐COHP for a bonding orbital, while a negative ‐COHP for an antibonding orbital by setting the Fermi level as an energy level of zero. The integrated value of ‐COHP is referred to as ‐iCOHP, where higher ‐iCOHP value indicates a stronger bond, and lower value a weaker bond. The ‐iCOHP values of S1‐S2 bonds in Li_2_S_2_ are 5.75 eV on the NiS_2_, 5.16 eV on the Ni_0.25_Co_0.75_S_2_, and 5.62 eV on the CoS_2_ (Figure 7d). Additionally, from the ‐COHP graph, it is observed that bonding orbitals in the Ni_0.25_Co_0.75_S_2_ are more prominent compared to pure NiS_2_ or CoS_2_ (Figure 7e). This implies that when Li_2_S_2_ is adsorbed on the Ni_0.25_Co_0.75_S_2_, the bonding between sulfur atoms in Li_2_S_2_ is most significantly weakened. The volcano plot clearly shows that the appropriate adsorption of Li_2_S_2_ brings out the weakest sulfur bond strength in Li_2_S_2_, finally resulting in the highest capacity decay rate at 1.0 C (Figure 7f). Moreover, the relative free energy for the conversion from Li_2_S_2_ to Li_2_S was calculated using following equation:

(2) $$E=E_{Li_{2}S+substrate}+\frac{1}{8}E_{S_{8}}-E_{Li_{2}S_{2}+substrate}$$

Figure S40 illustrates how the Li_2_S_2_ and Li_2_S molecules adsorb on the NiS_2_, Ni_0.25_Co_0.75_S_2_, and CoS_2_ surfaces, and the corresponding relative free energy diagram was calculated as shown in Figure S41 (Supporting Information). The required energies for the conversion reaction were 0.685 eV, 0.273 eV, and 0.528 eV, emphasizing the importance of optimal configuration of Ni/Co in Ni_x_Co_1‐x_S_2_ catalyst. Therefore, the moderate adsorption strength through cooperative catalyst brings outstanding battery performance.

**Figure 8:**
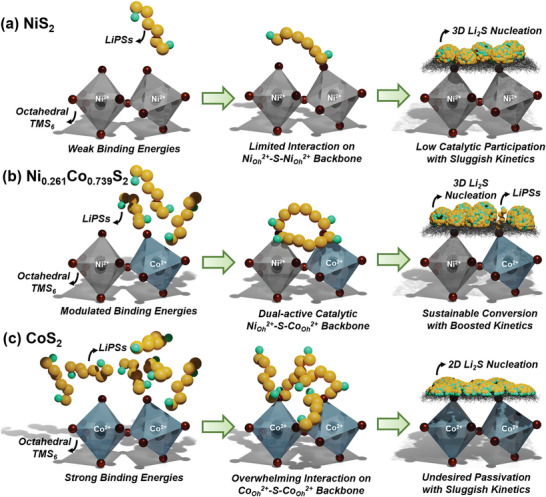
Schematic illustration of the underlying LiPSs adsorption and conversion mechanism of the a) NiS_2_, b) Ni_0.261_Co_0.739_S_2_, and c) CoS_2_ cathode catalysts.

The underlying stepwise conversion mechanism of nucleation and geometry‐dependent catalysis can be schematically summarized in **Figure** **8**a‐c based on the experimental and calculated results. Revaluated binding energy triggered from co‐existence of Ni_Oh_^2+^ and Co_Oh_^2+^ sites on Ni_Oh_^2+^−S−Co_Oh_^2+^ backbones can cooperatively convert from LiPSs to Li_2_S_2_, and subsequently activate diffusion‐controlled 3D Li_2_S nucleation via loosened S‐S bonding of Li_2_S_2_. Then, the catalytic sites of Ni_0.261_Co_0.739_S_2_ could maintain the activation state for sustainable sulfur redox process, which is rather hindered for the two cases of single metal sulfide; too weak LiPSs interactions on Ni_Oh_^2+^−S−Ni_Oh_^2+^ backbones for the NiS_2_ should induce a low Li_2_S deposition capacity and too high affinity for LiPSs on Co_Oh_^2+^ sites with vacant S sites in Co_Oh_^2+^−S−Co_Oh_^2+^ backbones for the CoS_2_ could lead to an undesired Li_2_S passivation with a low capacity.

## 3 Conclusion

In summary, we introduced the Ni_x_Co_1‐x_S_2_@NPCTs composite electrocatalyst as a highly efficient sulfur electrode material of Li‐S battery. The physical sulfur‐confinement in voids of composite catalyst structure and chemical interaction of LiPSs with doped‐nitrogen species and Ni_x_Co_1‐x_S_2_ nanocrystals were effectively boosted with a maximized sulfur utilization. Through the various physical, chemical, and electrochemical analyses, the concrete roles of TM‐S bonds in the octahedral TMS_6_ structures of Ni_x_Co_1‐x_S_2_ electrocatalysts were unveiled by the comprehensive experimental results in support of the theoretical DFT calculations. To be specific, the facilitated and efficient reduction processes of lithium polysulfides were realized on the proposed dual‐functional sites of Ni_x_Co_1‐x_S_2_@NPCTs cathode catalyst; sulfiphilic Co_Oh_^2+^ species with vacant S sites in Co‐S bonds for octahedral CoS_6_ structure can strongly immobilize polysulfides persistently to suppress shuttle effect, and subsequent redox conversion is conducted on the sites of Ni_Oh_^2+^−S−Co_Oh_^2+^ backbones, which could be ascribed to partial introduction of Ni_Oh_^2+^ in the octahedrally‐coordinated sites with reduced binding energies for sustainable adsorption‐catalysis process. This mechanistic scheme appeared to work best for an optimal configuration of Ni_0.261_Co_0.739_S_2_@NPCTs leading to 3D Li_2_S growth. This cathode catalyst system was able to achieve long‐term cycling stability showing 72.8% retention with a very low decay rate of 0.054% or less per cycle for 500 cycles at 1.0 C, and 0.055% decay per cycle even after 1000 cycles at 5.0 C owing to overall low overpotentials and interfacial resistances. Furthermore, the proposed cathode catalyst also demonstrated that it is possible to deliver excellent performances even under practically harsh conditions of low electrolyte concentrations and high sulfur loading cases. Especially, a high areal capacity of 2.20 mAh cm^−2^ was achieved even after 200 cycles at 0.2 C under 4.61 mg cm^−2^ sulfur loading condition. Theoretically, DFT calculations also supported the same results that the simulated Ni_0.25_Co_0.75_S_2_ catalyst can provide an optimal adsorption strength showing weaker S1‐S2 bond strength and longer bond length of Li_2_S_2_ molecules. This characteristic should allow the conversion of Li_2_S_2_ to Li_2_S more facile on the proposed catalytic surface, resulting in the higher performance at a higher C‐rate process. Overall, the present study provided novel guidelines of the rational design of hybrids with multi‐functional carbon backbone and elemental‐manipulated electrocatalysts for efficient redox reaction via stepwise catalytic mechanism. We believe that our strategies could pave a promising way of universal exploration for designing high‐performance Li‐S battery cathode catalysts with a new perspective.

## 4 Experimental Section

Detailed experimental procedures can be found in the Supporting Information.

## Conflict of Interest

The authors declare no conflict of interest.

## Supporting information

Supporting Information

## Data Availability

The data that support the findings of this study are available from the corresponding author upon reasonable request.
