# Supplementary material for: Accelerated Conversion of Polysulfides for Ultra Long‐Cycle of Li‐S Battery at High‐Rate over Cooperative Cathode Electrocatalyst of Ni0.261Co0.739S2/N‐Doped CNTs
Source: Adv Sci (Weinh). 2024 Jun 12;11(32):2402389. doi: 10.1002/advs.202402389 (PMC11348136; doi:10.1002/advs.202402389)
Supplement: Supplementary file 1 — Supporting Information [file ADVS-11-2402389-s001.docx]

Supporting Information

**Accelerated Conversion of Polysulfides for Ultra Long-Cycle of Li-S Battery at High-Rate over Cooperative Cathode Electrocatalyst of Ni_0.261_Co_0.739_S_2_/N-doped CNTs**

*Junhyuk Ji, Minseon Park, Minho Kim, Song Kyu Kang, Gwan Hyeon Park, Junbeom Maeng, Jungseub Ha, Min Ho Seo^*^,Won Bae Kim^*^*

J. Ji, M. Park, M. Kim, S. K. Kang, G. H. Park, J. Maeng, J. Ha, Prof. W. B. Kim

Department of Chemical Engineering, Pohang University of Science and Technology (POSTECH), 77 Cheongam-ro, Nam-gu, Pohang-si, Gyeongsangbuk-do 37673, Republic of Korea

Prof. M. H. Seo

Department of Nanotechnology Engineering, Pukyong National University (PKNU), 45 Yongso-ro, Nam-gu, Busan-si 48513, Republic of Korea

Prof. W. B. Kim

Graduate Institute of Ferrous & Eco Materials Technology, Pohang University of Science and Technology (POSTECH), 77 Cheongam-ro, Nam-gu, Pohang-si, Gyeongsangbuk-do 37673, Republic of Korea

E-mail: foifrit@pknu.ac.kr (M. H. Seo), kimwb@postech.ac.kr (W. B. Kim)

Keywords: Li-S battery, Nickel cobalt sulfide, Cathode catalysts, Lithium polysulfides, N-doped porous carbon, Cooperative catalysis

**Experimental section**

*Materials and chemicals*

Cobalt (II) nitrate hexahydrate (Co(NO_3_)_2_·6H_2_O, ≥ 98%), poly(vinylidene fluoride-co-hexafluoro-propylene) (PVDF-HFP, average *M*_w_ ca. 400,000, and average *M*_n_ ca. 130,000), sodium dodecylbenzenesulfonate (SDBS, CH_3_(CH_2_)_11_C_6_H_4_SO_3_Na, technical grade), and sulfur were purchased from Sigma-Aldrich. 1-Methyl-2-pyrrolidinone (C_5_H_9_NO, anhydrous, 99.5%), lithium sulfide (Li_2_S, metal basis, 99.9%), 1,3-dioxlane (C_3_H_6_O_2_, 99.5%) and 1,2-dimethoxyethane (C_4_H_10_O_2_, 99+%) were purchased from Alfa Aesar. Pyrrole (C_4_H_5_N, 99%) was purchased from Acros Organics. Methyl orange (C_14_H_14_N_3_NaO_3_S), nickel (II) nitrate hexahydrate (Ni(NO_3_)_2_·6H_2_O, ≥ 98.0%), thiourea (CH_4_N_2_S, 98.0%), and 1-butanol (C_4_H_10_O, 99.0%) were purchased from Samchun chemicals. Iron (III) chloride (FeCl_3_, anhydrous, 98%), absolute ethanol (C_2_H_5_OH), and acetone (C_3_H_6_O) were purchased from Thermo Fisher Scientific. Multiwalled carbon nanotube (10 - 20 nm diameter, 10 - 30 μm length) was purchased from Nanostructured & Amorphous Materials Inc. All reagents were used without additional purification. Deionized (DI) water (resistivity > 18 MΩ) was used for all experimental procedures.

*Preparation of Ni_x_Co_1-x_S_2_ embedded hybrid host materials*

First of all, we dissolved 0.0818 g methyl orange in 50 mL distilled water. Afterwards, FeCl_3_ (0.406 g) and SDBS (0.0176 g) were added to the pre-mixture sequentially under magnetic stirring. After 2 h, 170 μl of pyrrole was dropped slowly with continuous stirring for overnight. Finally, obtained black powder was washed and dried. To fabricate the desired morphology of nitrogen-doped porous carbon nanotubes (NPCTs), powders were annealed at 650 ^o^C under argon atmosphere for 5 h with ramping rate of 3 ^o^C min^-1^. Then, we dissolved 1.163 g nickel nitrate hexahydrate in 20 mL 1-butanol under vigorous stirring at 80 ^o^C and added 1.216 g thiourea, then nickel-thiourea product (Ni(TU)_4_(NO_3_)_2_) was obtained. Second, we synthesized cobalt-thiourea complexes (Co(TU)_4_(NO_3_)_2_) through the same method except the use of 1.164 g cobalt nitrate hexahydrate instead of nickel precursor. The acquired solids were washed and dried. Subsequently, 0.10 g NPCTs were well-dispersed in 15 mL acetone under ultra-sonication, to which the nickel-thiourea complex (20 mg) and cobalt-thiourea complex (30 mg) were included. The pre-obtained mixture were evaporated at 35 ^o^C with magnetic stirring, and calcined at 400 ^o^C for 2 h under argon atmosphere with ramping rate of 5 ^o^C min^-1^. Finally, we attained the nickel-cobalt sulfide nanocrystals adsorbed NPCTs (labeled as Ni_0.261_Co_0.739_S_2_@NPCTs). We also fabricated the other composites by a similar procedure.

*Synthesis of hybridized NiS_2_@NPCTs host materials*

The synthetic procedure of NiS_2_@NPCTs was similar to that of Ni_0.261_Co_0.739_S_2_@NPCTs except that 50 mg of nickel-thiourea complex was added to NPCTs-dispersed mixture without cobalt-thiourea complex during the fabrication process.

*Synthesis of hybridized Ni_0.679_Co_0.321_S_2_@NPCTs host materials*

The synthetic procedure of Ni_0.679_Co_0.321_S_2_@NPCTs was similar to that of Ni_0.261_Co_0.739_S_2_@NPCTs except that 40 mg of nickel-thiourea complex and 10 mg of cobalt-thiourea complex were added to NPCTs-dispersed mixture during the fabrication process.

*Synthesis of hybridized Ni_0.444_Co_0.556_S_2_@NPCTs host materials*

The synthetic procedure of Ni_0.444_Co_0556_S_2_@NPCTs was similar to that of Ni_0.261_Co_0.739_S_2_@NPCTs except that 30 mg of nickel-thiourea complex and 20 mg of cobalt-thiourea complex were added to NPCTs-dispersed mixture during the fabrication process.

*Synthesis of hybridized Ni_0.135_Co_0.865_S_2_@NPCTs host materials*

The synthetic procedure of Ni_0.135_Co_0.865_S_2_@NPCTs was similar to that of Ni_0.261_Co_0.739_S_2_@NPCTs except that 10 mg of nickel-thiourea complex and 40 mg of cobalt-thiourea complex were added to NPCTs-dispersed mixture during the fabrication process.

*Synthesis of hybridized CoS_2_@NPCTs host materials*

The synthetic procedure of CoS_2_@NPCTs was similar to that of Ni_0.261_Co_0.739_S_2_@NPCTs except that 50 mg of cobalt-thiourea complex was added to NPCTs-dispersed mixture without nickel-thiourea complex during the fabrication process.

*Materials characterization*

For details of chemicals and synthesis steps, see Supplementary Material. Morphology images were examined using SEM (JSM-7800F Prime; JEOL) with EDS at 5.0 kV, and TEM (JEM-2200FS; JEOL) with EELS at 200 kV. Crystal structure was investigated by XRD (Ultima IV; Rigaku) using a Cu-Kα radiation (λ = 1.5418 Å) source with a Ni filter at 40 kV and 30 mA, and SAED (JEM-2200FS; JEOL) was also applied for the same purpose. The XPS (K-Alpha^+^; Thermo Fisher Scientific) analysis was carried out with a monochromatic Al-Kα source (E = 1486.6 eV). Thermal decomposition data were obtained using thermal gravimetric analysis (TGA, SDT Q600; TA instruments). The BET method was applied by using N_2_ adsorption–desorption (ASAP 2020 BET/porosimeter; Micromeritics). FT-IR (Nicolet iS50; Thermo Fisher) signals were recorded as transmittance spectra. Raman spectrophotometer (NRS-5100; JASCO) was used equipped with a 532 nm excitation laser of notch. Metal concentrations of catalysts were characterized by ICP-AES (ICAP 6000; Thermo Fisher Scientific). Rietveld refinement was performed by using synchrotron high-resolution XRD patterns which were obtained at the 9B HRPD beamline of the Pohang Light Source (PLS-II) with a monochromatic X-ray wavelength of 1.5309 Å. Ex-situ SAXS experiments were conducted at the 6D C&S UNIST SAXS beamline of PLS-II with a wavelength of 1.07216 Å and a 11.564 keV X-ray energy. Powder and electrode K-edge X-ray absorption fine structure (XAFS) data were collected at the 7D XAFS and 10C Wide-XAFS beamlines of PLS-II in the transmission and fluorescence modes.

*Synchrotron-based small-angle X-ray scattering analysis*

SAXS was measured by PLS-II 6D UNIST-PAL beamline of PAL with a wavelength of 1.07216 Å. The sample-to-detector distance (SDD) was 3.0 m and the diffraction data were detected with a 2D CCD detector (MX225-HS, Rayonix) in a transmission mode. All the parameters from core-shell cylinder model are calculated as follows:

$I\left( q,\alpha\right)= \frac{scale}{V_{s}}F^{2}\left( q,\alpha\right)\sin(\alpha)+background$ (S1)

$F\left( q,\alpha\right)=\frac{\left( \rho_{c}-\rho_{s} \right)V_{c}\sin\left( q\frac{1}{2}L\cos\alpha\right)}{q\frac{1}{2}L\cos\alpha}\frac{2J_{1}\left( qR\sin\alpha\right)}{qR\sin\alpha}+\frac{\left( \rho_{s}-\rho_{solv} \right)V_{s}sin \left( q\left( \frac{1}{2}L+T \right)\cos\alpha\right)}{q\left( \frac{1}{2}L+T \right)\cos\alpha}\frac{2J_{1}\left( q(R+T)\sin\alpha\right)}{q(R+T)\sin\alpha}$ (S2)

$V_{s}=\pi\left( R+T \right)^{2}\left( L+2T \right)$ (S3)

, where *α, V*_s_, *V*_c_, *L*, *R*, *T*, *ρ*_c_, *ρ*_s_, *ρ*_solv_, background, and *J*_1_ represent angle between the axis of the cylinder and *q*, total volume including both the core and the outer shell, volume of the core, length of the core, radius of the core, thickness of the shell, scattering length density of the core, shell, and solvent, background level, and the first order Bessel function, respectively. All the parameters from the Guinier-Porod function are calculated as follows:

$I\left( q \right)=\left\{ \begin{aligned} \frac{G}{Q^{s}}\exp\left[ -\frac{Q^{2}R_{g}^{2}}{3-s} \right] Q{\leq Q}_{1} \\ D/Q^{m} Q{\geq Q}_{1} \end{aligned} \right.$ (S4)

$Q_{1}=\frac{1}{R_{g}}\sqrt{(m-s)(3-s)/2}$ (S5)

$D=G exp\left[ \frac{-Q^{2}R_{g}^{2}}{3-s} \right]Q_{1}^{m-s}=\frac{G}{R_{g}^{m-s}}exp\left[ -\frac{m-s}{2} \right]\left( \frac{(m-s)(3-s)}{2} \right)^{\frac{m-s}{2}}$ (S6)

, where *R*_g_, *s*, and *m* are radius of gyration, dimension variable, and Porod exponent, respectively.

*Analysis of the Li_2_S_6_ adsorption properties*

0.2 mM Li_2_S_6_ electrolyte solution was prepared by mixing lithium sulfide and sulfur at a molar ratio of 8:5 in DOL/DME (1,3-dioxlane/1,2-dimethoxyethane) solvent (v/v% = 1:1) at 90 ^o^C. For the adsorption measurement, 100 mg of carbon-catalyst composites (Ni_x_Co_1-x_S_2_@NPCTs) and catalyst-only samples (Ni_x_Co_1-x_S_2_) were immersed in the solution for 24 h. The resultant solutions were collected in UV-cuvette, then UV-visible spectroscopy (Cary 8454; Agilent Technologies) was used to examine the change in concentration of solution in terms of UV-visible absorption spectra.

*Electrochemical measurements*

Active material of Ni_x_Co_1-x_S_2_@NPCTs/S was produced by mixing the elemental sulfur (63.6 wt.%) and Ni_x_Co_1-x_S_2_@NPCTs (36.4 wt.%) powder and followed by heating at 155 ^o^C for 12 h. The active material (75 wt.%) was further mixed with MWCNT conductive agent (15 wt.%) and PVDF-HFP (poly(vinylidene fluoride-co-hexafluoro-propylene)) binder (10 wt.%) in NMP (1-methyl-2-pyrrolidinone), and the mixture was casted on carbon-coated aluminum foil, then dried at 60 ^o^C in vacuum overnight. Areal sulfur loading was 1.5 mg cm^-2^ with circular diameter of 12 mm. CR2032-type coin cells were assembled in an argon-filled glove box and tested on battery system (WBCS 3000; Won-A Tech) strictly maintained at 25 ^o^C. The electrolyte was 1.0 M solution of lithium bis(trifluoromethanesulfonyl)imide (LiTFSI) in DOL/DME (v/v = 1:1) with 2 wt.% of LiNO_3_ as an additive. The E/S ratio was set to 30 μL mg^-1^. For the galvanostatic charge-discharge, the voltage range was controlled over 1.7 - 2.8 V (vs. Li/Li^+^). CV was conducted with the same voltage range at multiple scan rates from 0.03 to 0.20 mV s^-1^. Cathodes of the symmetric cell were fabricated by mixing the Ni_x_Co_1-x_S_2_@NPCTs (80 wt.%) with PVDF-HFP (20 wt.%), and the test was carried out in a voltage range from - 1.5 V to 1.5 V at 0.10 mV s^-1^ with catholyte containing 0.5 M Li_2_S_6_ in the electrolyte. For Li_2_S nucleation analysis, chronoamperometry was performed at 2.03 V (vs. Li/Li^+^) for 10,000 s with the same catholyte. EIS was conducted with amplitude of 5 mV over the frequency range in 100 kHz - 10 mHz using a sinusoidal voltage by ZIVELAB potentiostat.

*Operando XRD analysis*

Synchrotron-based operando analysis for in-situ XRD variations on the cathode was performed using the 1D XRS KIST-PAL beamline of PLS-II with a wavelength of 1.0000 Å and an energy of 12.3984 keV, and the 9A U-SAXS beamline of PLS-II with an X-ray energy of 19.805 keV. The XRD data were periodically collected with an interval of 1.5 min on a MAR 345-image plate detector in a transmission mode.

*Computational details*

DFT calculations were conducted employing the Vienna Ab-initio Simulation Package (VASP) program.^[1,2]^ Projector Augmented Wave (PAW) pseudopotentials^[3]^ with cutoff energy of 520 eV and revised Perdew-Burke-Ernzerhof functionals (RPBE) were applied for further improvement of surface.^[4]^ The relaxation of all (200) surfaces of NiS_2_, Ni_0.25_Co_0.75_S_2_, and CoS_2_ was constructed from relaxed bulk unit cell,^[5]^ and separated by a vacuum of 20 Å to avoid interactions between the top and bottom surfaces in the unit cell (Figure S42). Nickel-cobalt sulfide models were sampled using gamma point with (3,3,1) k-points and the gaseous Li_2_S_2_ molecule was computed in a (20x20x20) Å cell with gamma point of (1,1,1) k-points. DFT-D3 was chosen to consider the van der Waals interactions.^[6]^ A crystal orbital Hamilton population (COHP) analysis was carried out to show the bonding properties of sulfur atoms in Li_2_S_2_ molecules on nickel-cobalt sulfide surfaces, by separating bonding orbitals and antibonding orbitals.^[7]^


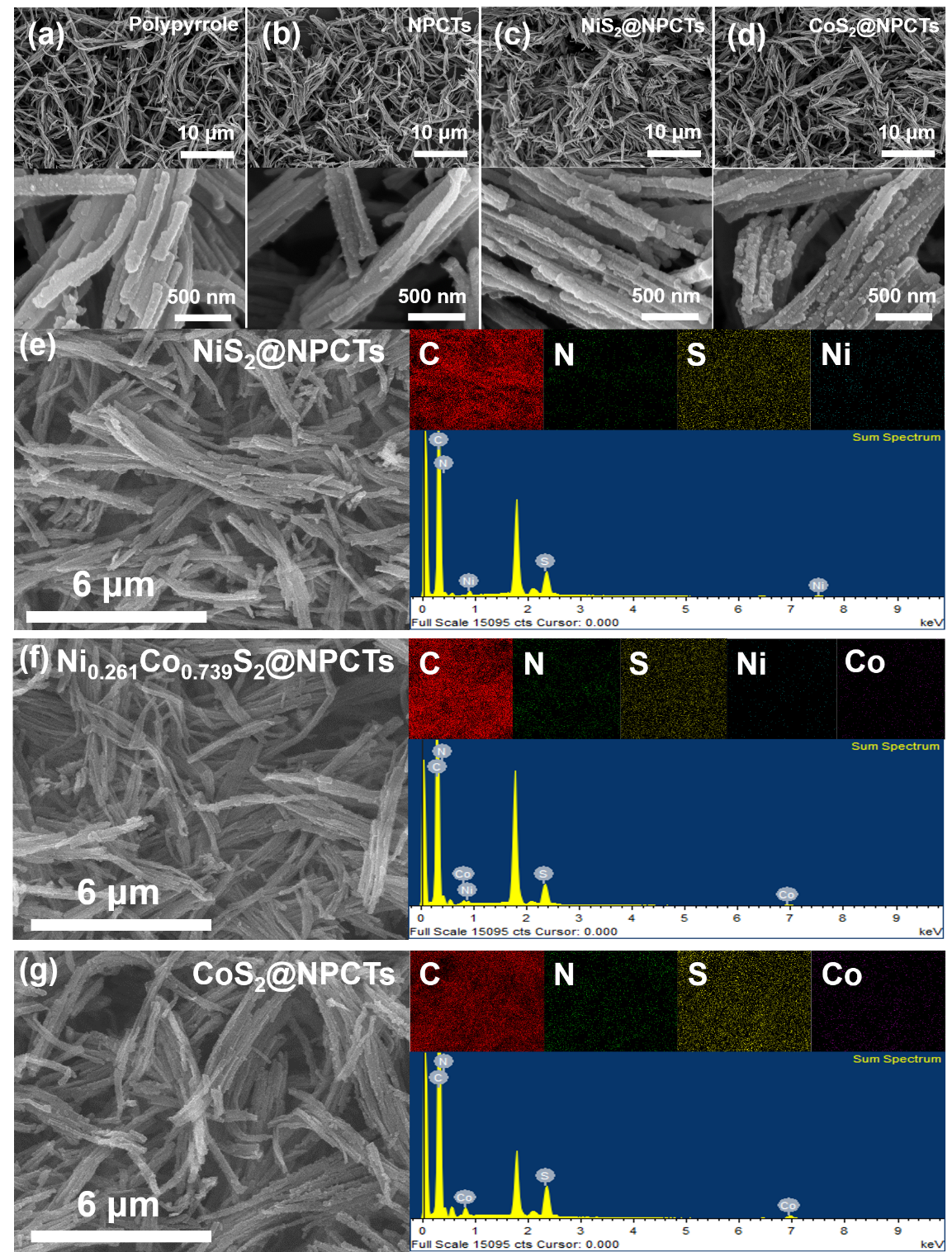


**Figure S1.** (a-d) SEM images with magnified versions of the polypyrrole, NPCTs, NiS_2_@NPCTs, and CoS_2_@NPCTs samples. SEM images for EDS mapping area with resultant EDS spectra of the (e) NiS_2_@NPCTs, (f) Ni_0.261_Co_0.739_S_2_@NPCTs, and (g) CoS_2_@NPCTs composites consisting of C (red), N (green), S (yellow), Ni (bluish green), and Co (purple) elements.


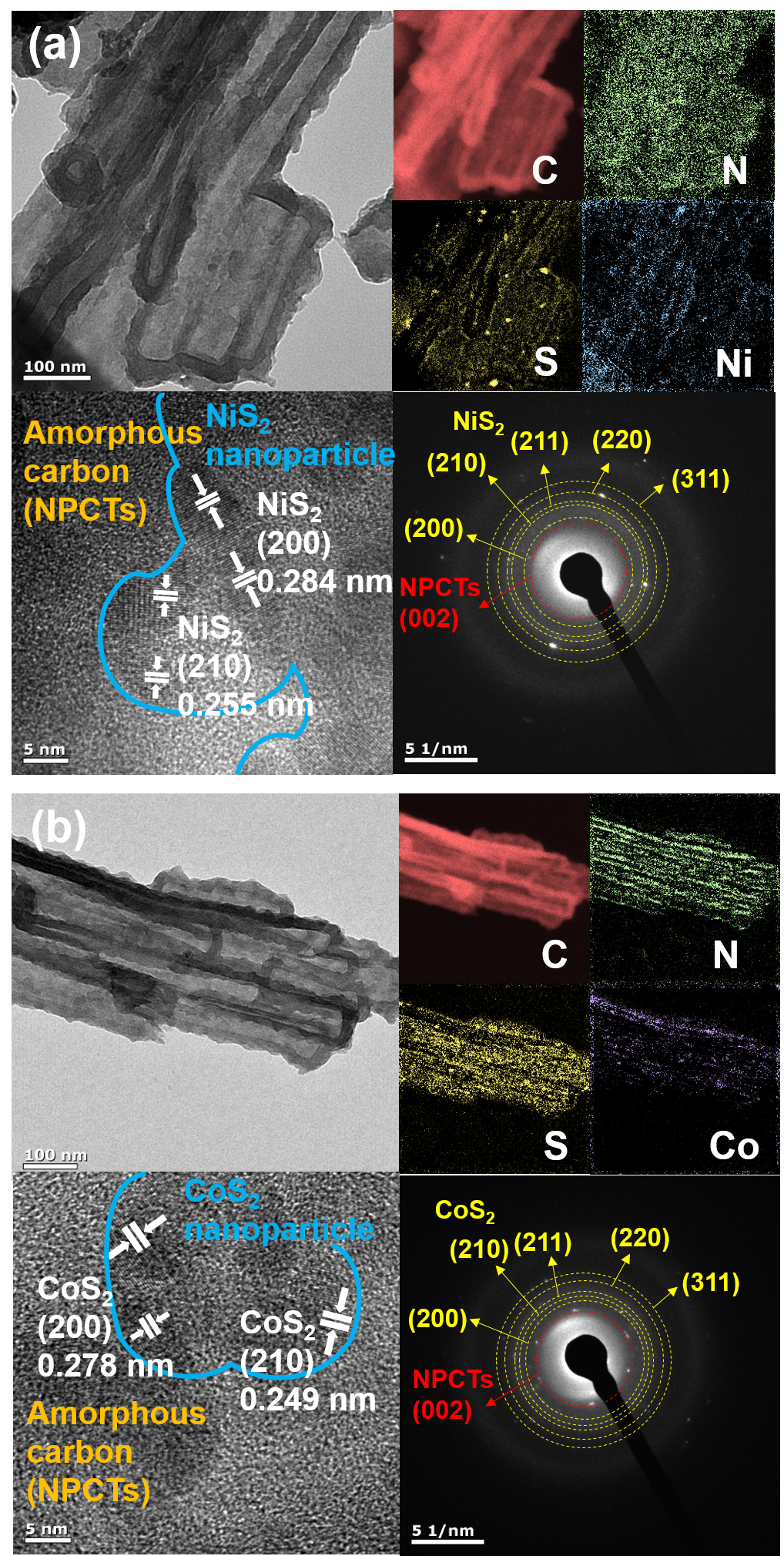


**Figure S2.** TEM images with EELS mappings (C, red; N, green; S, yellow; Ni, blue; Co, purple), HRTEM images (with metal sulfide nanocrystals highlighted in white lines), and SAED patterns of the (a) NiS_2_@NPCTs, and (b) CoS_2_@NPCTs composites.


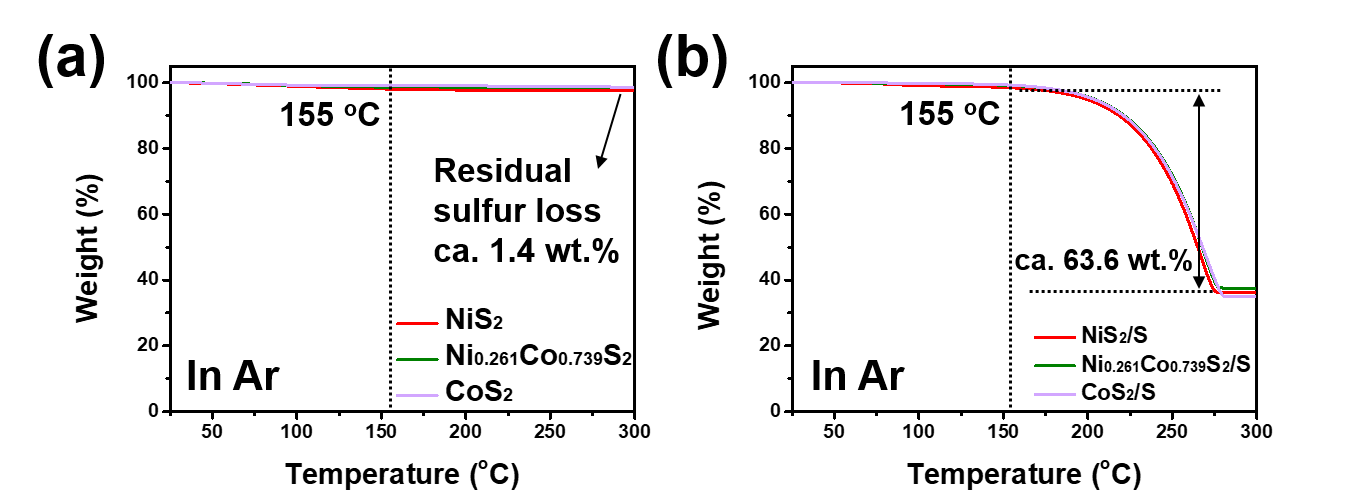


**Figure S3**. TGA analysis curves of (a) the Ni_x_Co_1-x_S_2_ and (b) the Ni_x_Co_1-x_S_2_/S samples under Ar condition.


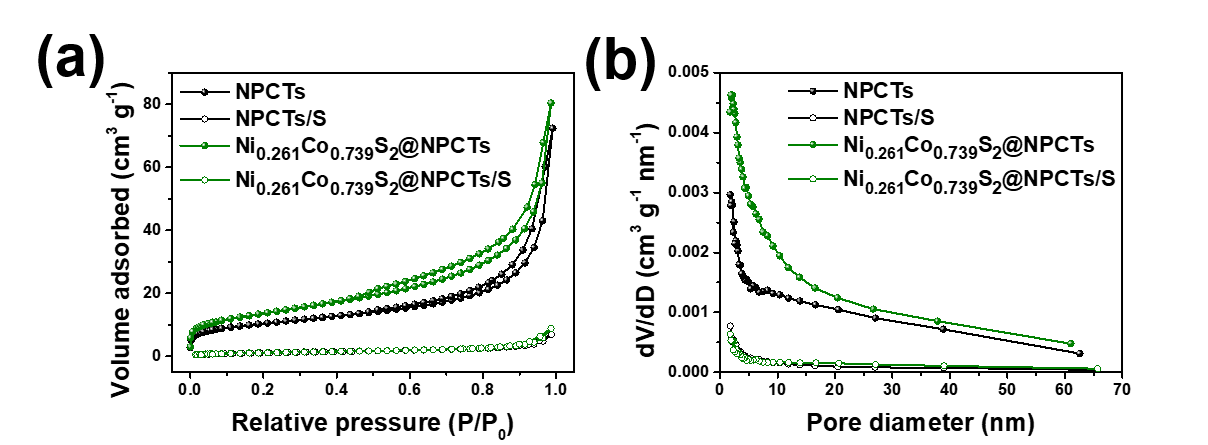


**Figure S4.** (a) N_2_ adsorption/desorption curves and (b) corresponding pore size distributions of the NPCTs, NPCTs/S, Ni_0.261_Co_0.739_S_2_@NPCTs, and Ni_0.261_Co_0.739_S_2_@NPCTs/S composites.


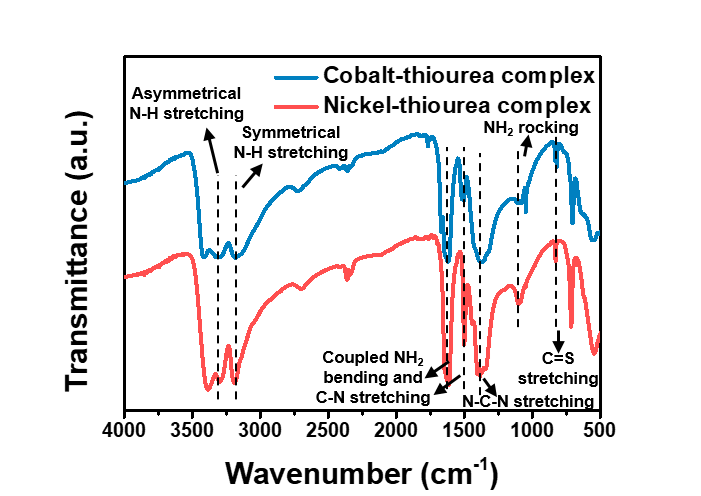


**Figure S5.** FT-IR spectra of nickel-thiourea complex (red) and cobalt-thiourea complex (blue) with assignment of the absorption bands at each specific wavenumber.


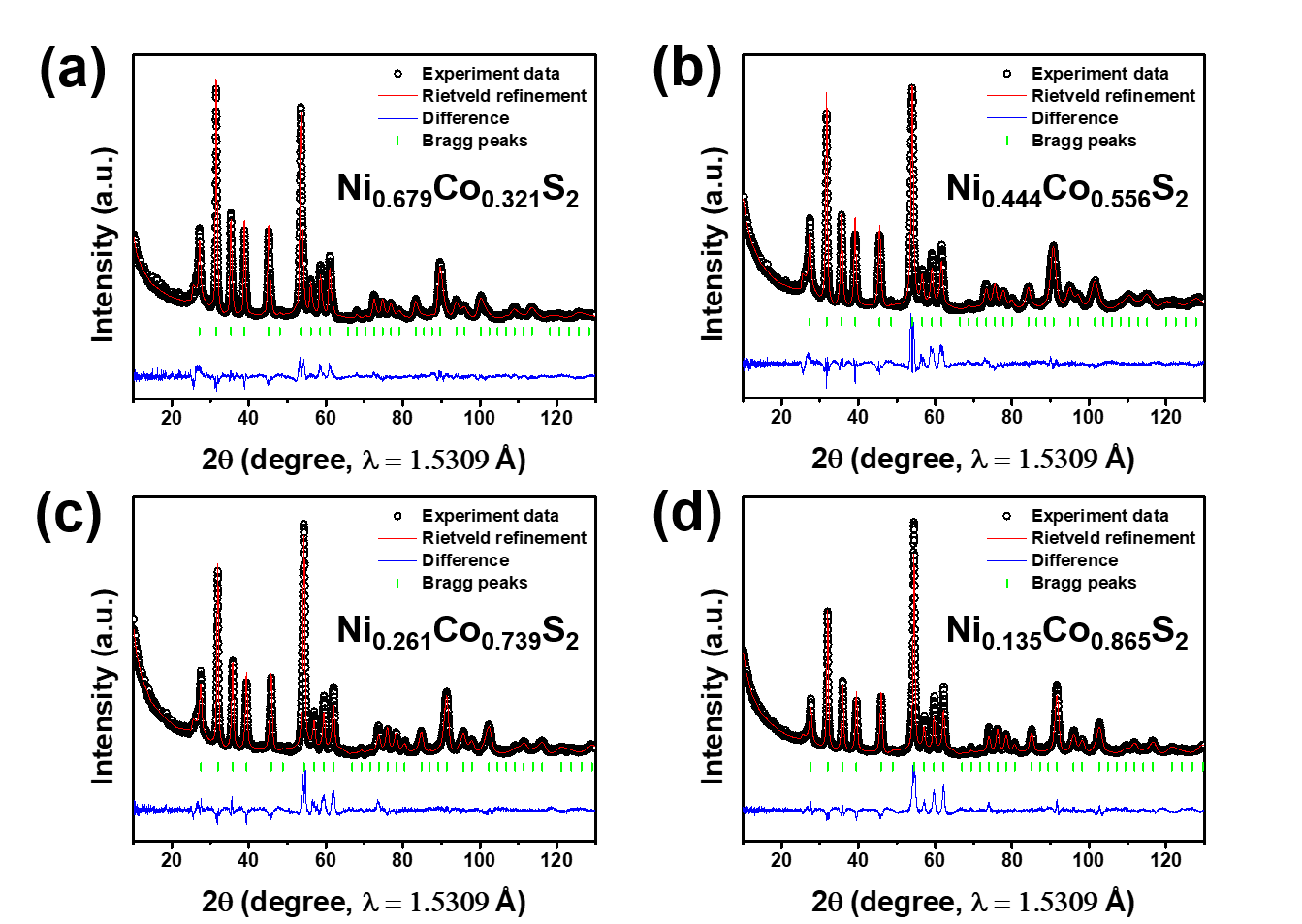


**Figure S6.** Rietveld refinement results against the HRPD patterns of the (a) Ni_0.679_Co_0.321_S_2_, (b) Ni_0.444_Co_0.556_S_2_, (c) Ni_0.261_Co_0.739_S_2_, and (d) Ni_0.135_Co_0.865_S_2_ catalysts.


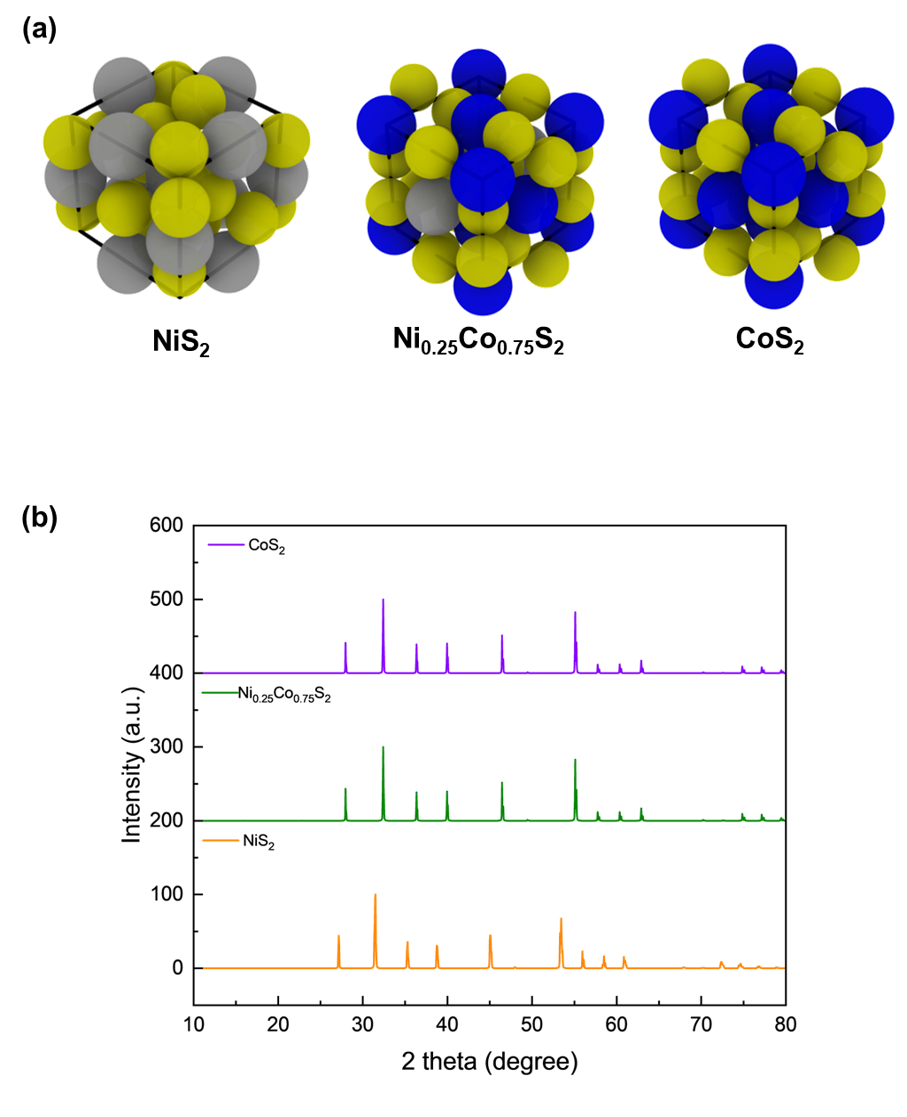


**Figure S7.** (a) Relaxed bulk models of 2x2x2 cell of NiS_2_, Ni_0.25_Co_0.75_S_2_, and CoS_2_ used to determine the lattice parameters via computational methods and (b) calculated XRD patterns.


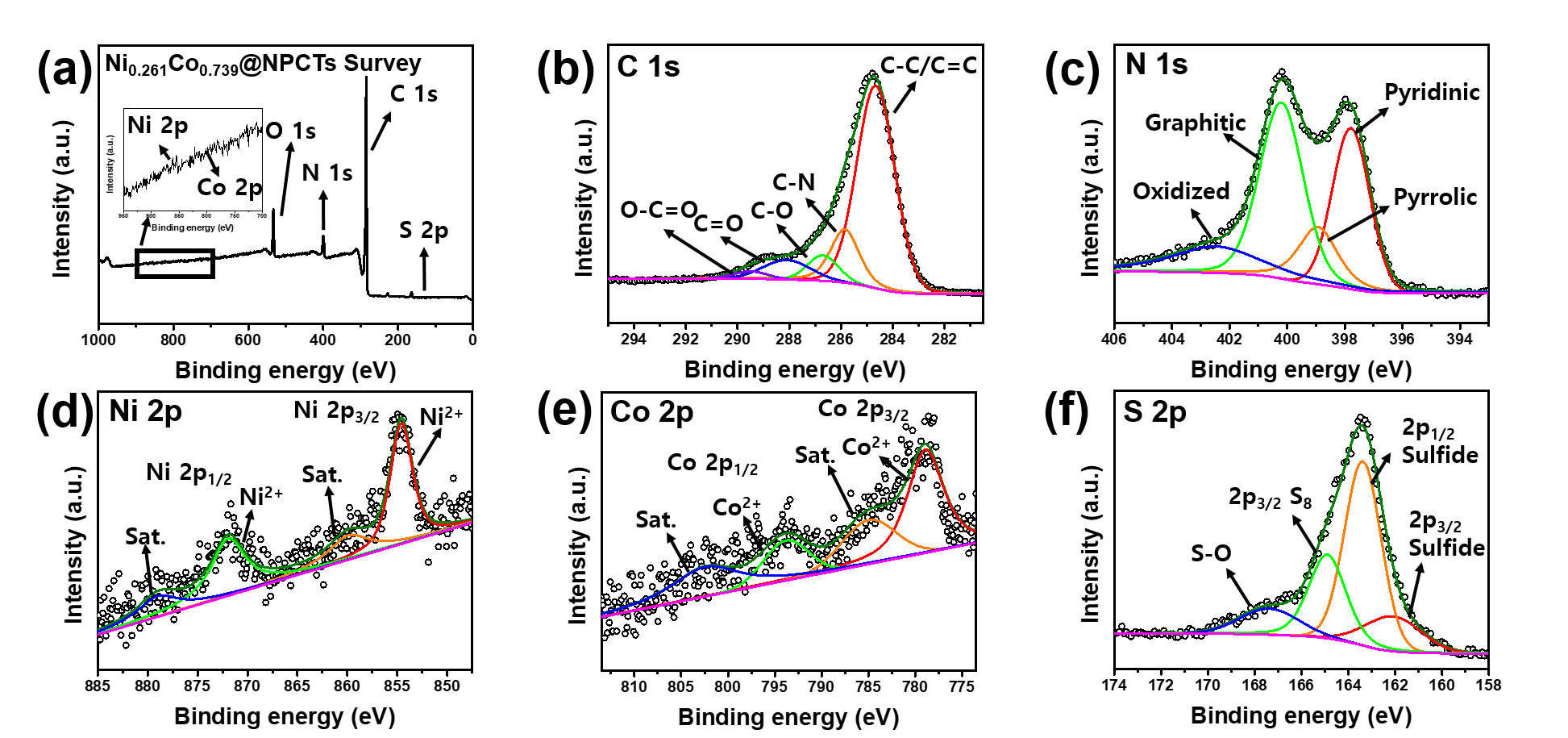


**Figure S8.** (a-f) XPS spectra for the Ni_0.261_Co_0.739_S_2_@NPCTs of survey scan, C 1s, N 1s, Ni 2p, Co 2p, and S 2p, respectively.


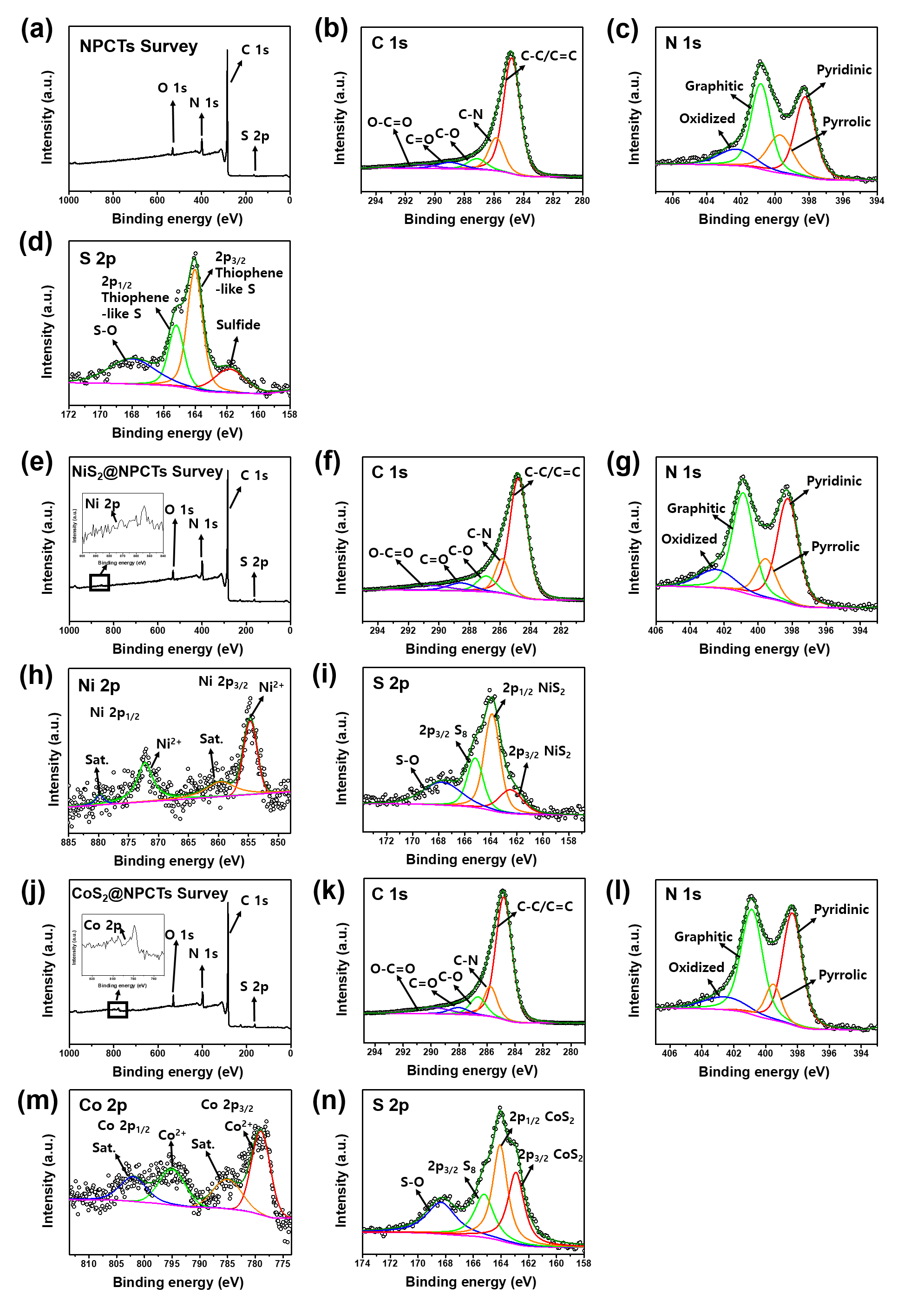


**Figure S9.** XPS spectra of survey scan, C 1s, N 1s, Ni 2p, Co 2p, and S 2p, respectively, for the (a-d) NPCTs, (e-i) NiS_2_@NPCTs, and (j-n) CoS_2_@NPCTs materials.


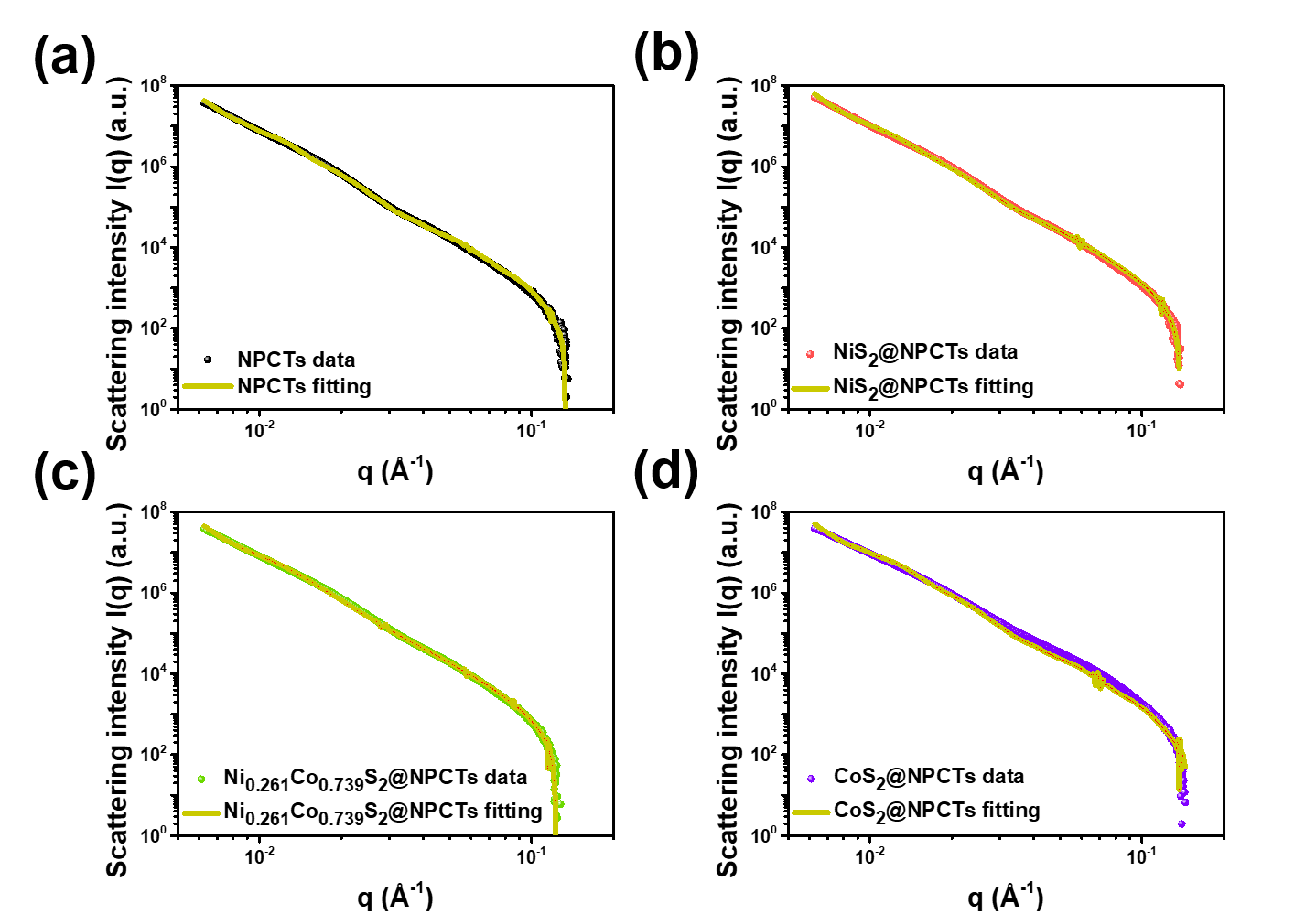


**Figure S10.** One-dimensional SAXS data and core-shell cylinder model fitting results of the (a) NPCTs, (b) NiS_2_@NPCTs, (c) Ni_0.261_Co_0.739_S_2_@NPCTs, and (d) CoS_2_@NPCTs samples.


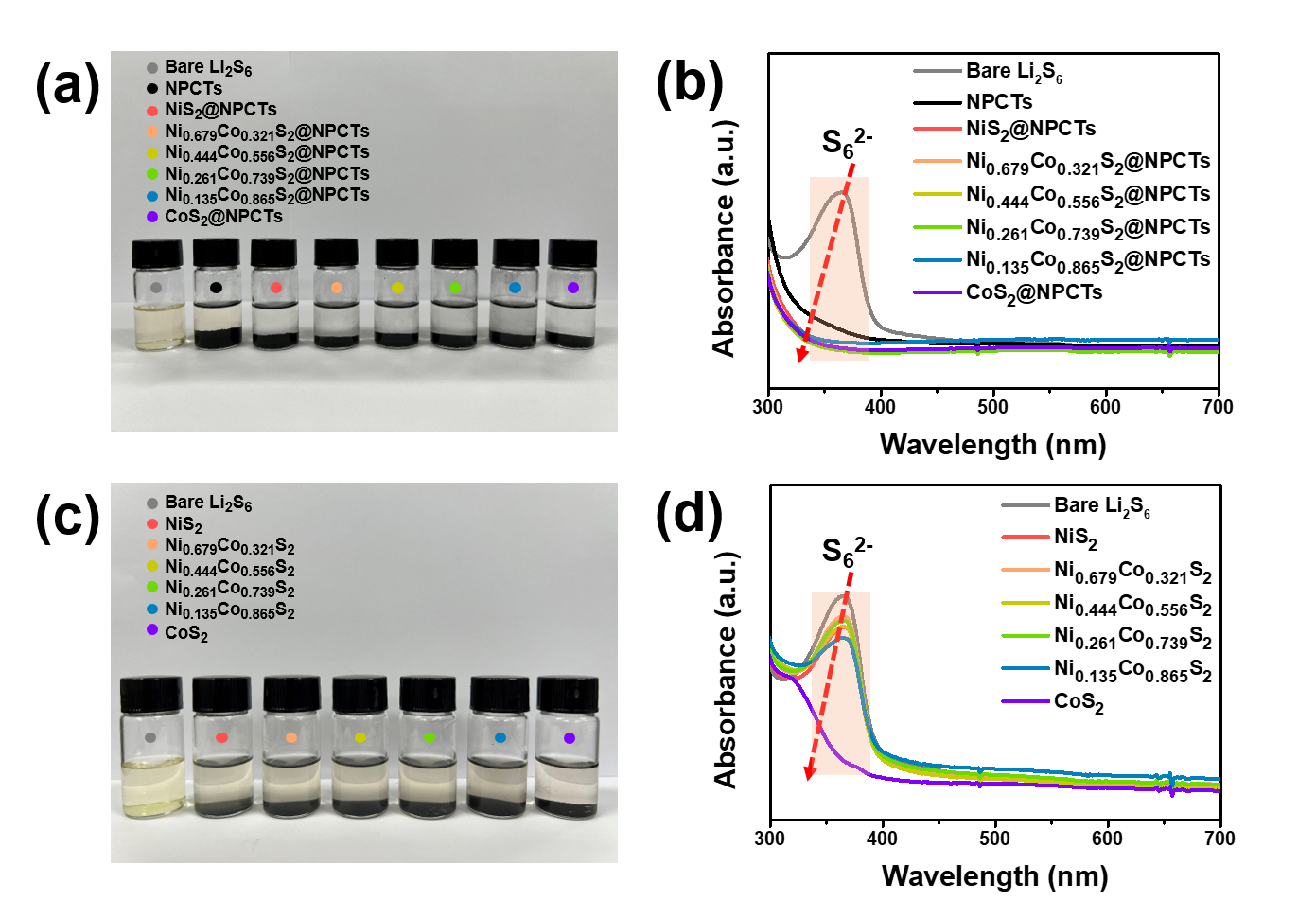


**Figure S11.** Two-type Li_2_S_6_ adsorption analyses with digital photographs of experimental setup and corresponding UV-Vis spectra for the various electrocatalysts: (a, b) catalyst-dispersed carbon hosts and (c, d) Ni_x_Co_1-x_S_2_ catalysts without carbon supports.


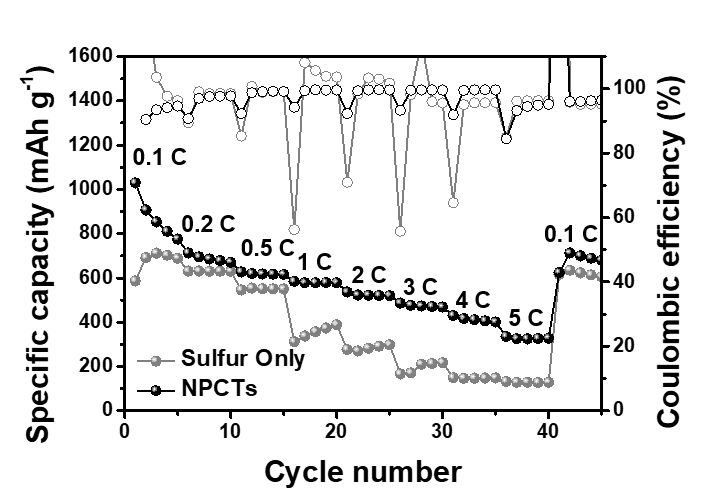


**Figure S12.** Rate capability performance of sulfur-only and NPCTs cathodes for comparison.


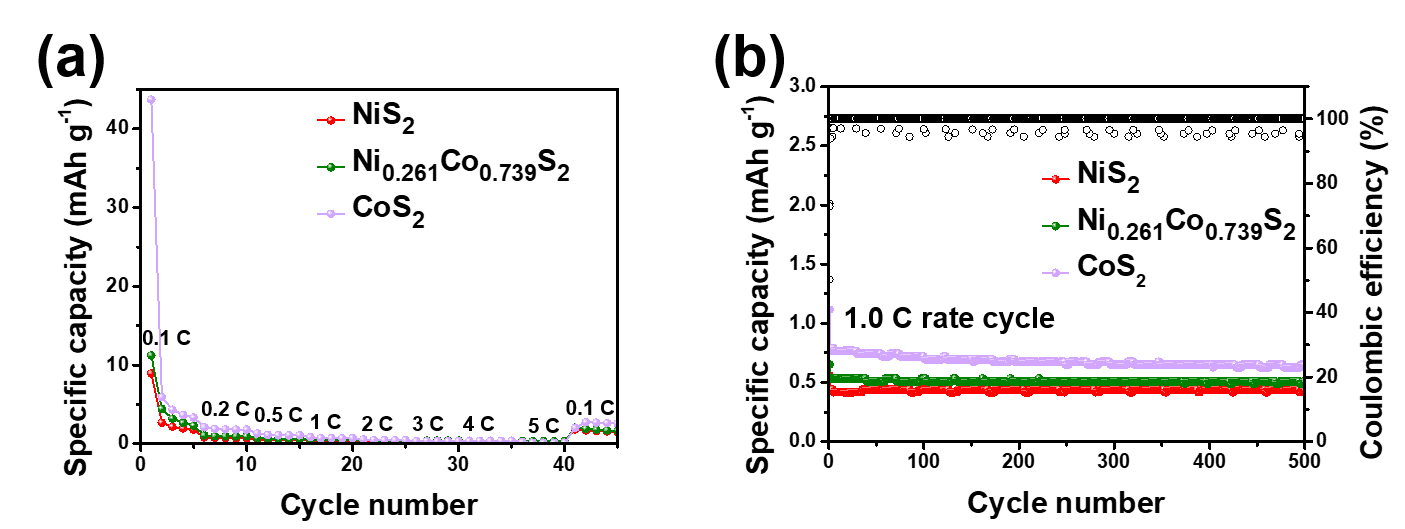


**Figure S13.** Electrochemical performances of the NiS_2_, Ni_0.261_Co_0.739_S_2_, and CoS_2_ used as catalyst materials without adding active sulfur elements. (a) Rate capability test, and (b) cycling performance at 1.0 C.


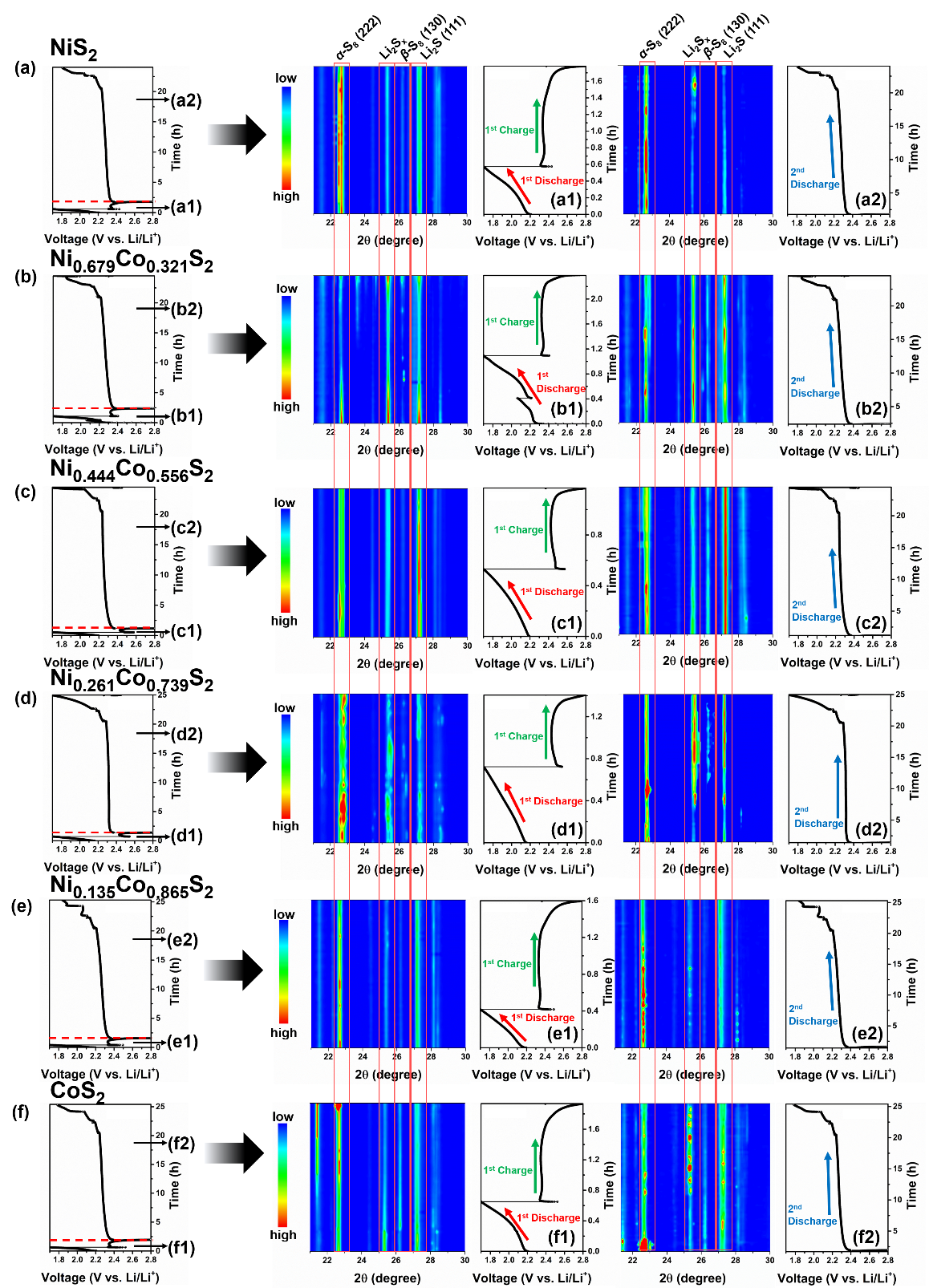


**Figure S14.** In-situ XRD patterns in contour plots as a function of discharge/charge process of the Li-S cells for the (a) NiS_2_, (b) Ni_0.679_Co_0.321_S_2_, (c) Ni_0.444_Co_0.556_S_2_, (d) Ni_0.261_Co_0.739_S_2_, (e) Ni_0.135_Co_0.865_S_2_, and (f) CoS_2_ cathodes.


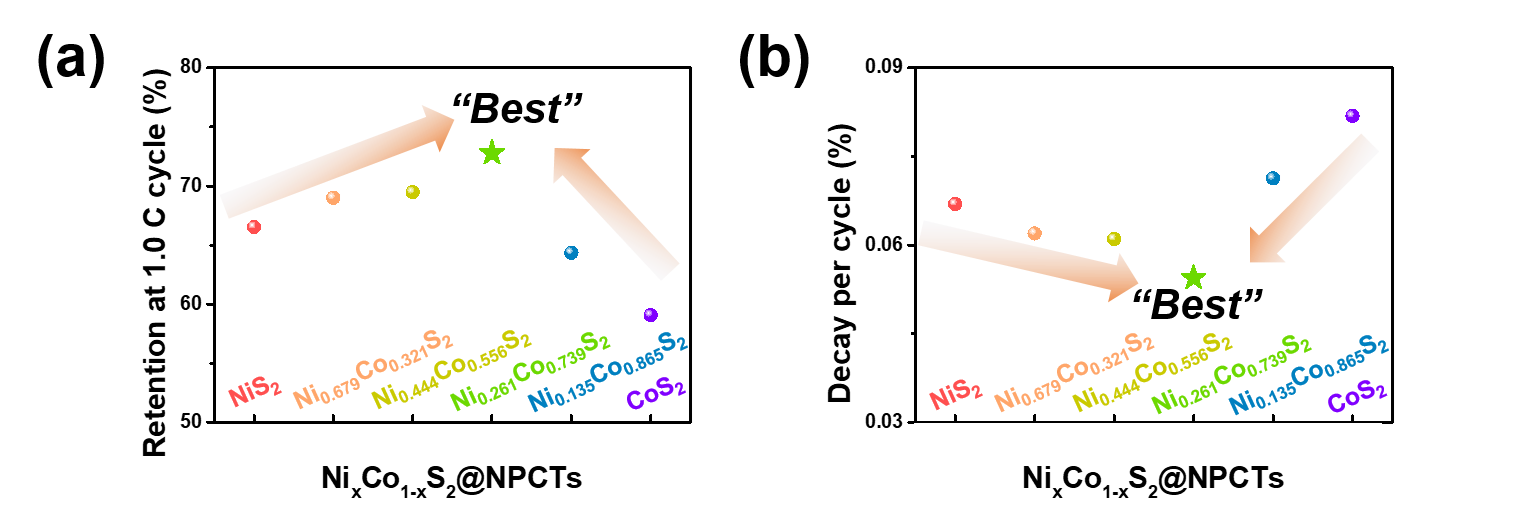


**Figure S15.** Volcano plots of the Ni_x_Co_1-x_S_2_@NPCTs cathode catalysts showing (a) retention at 1.0 C cycle and (b) capacity decay rate during cycling performances at 1.0 C rate.


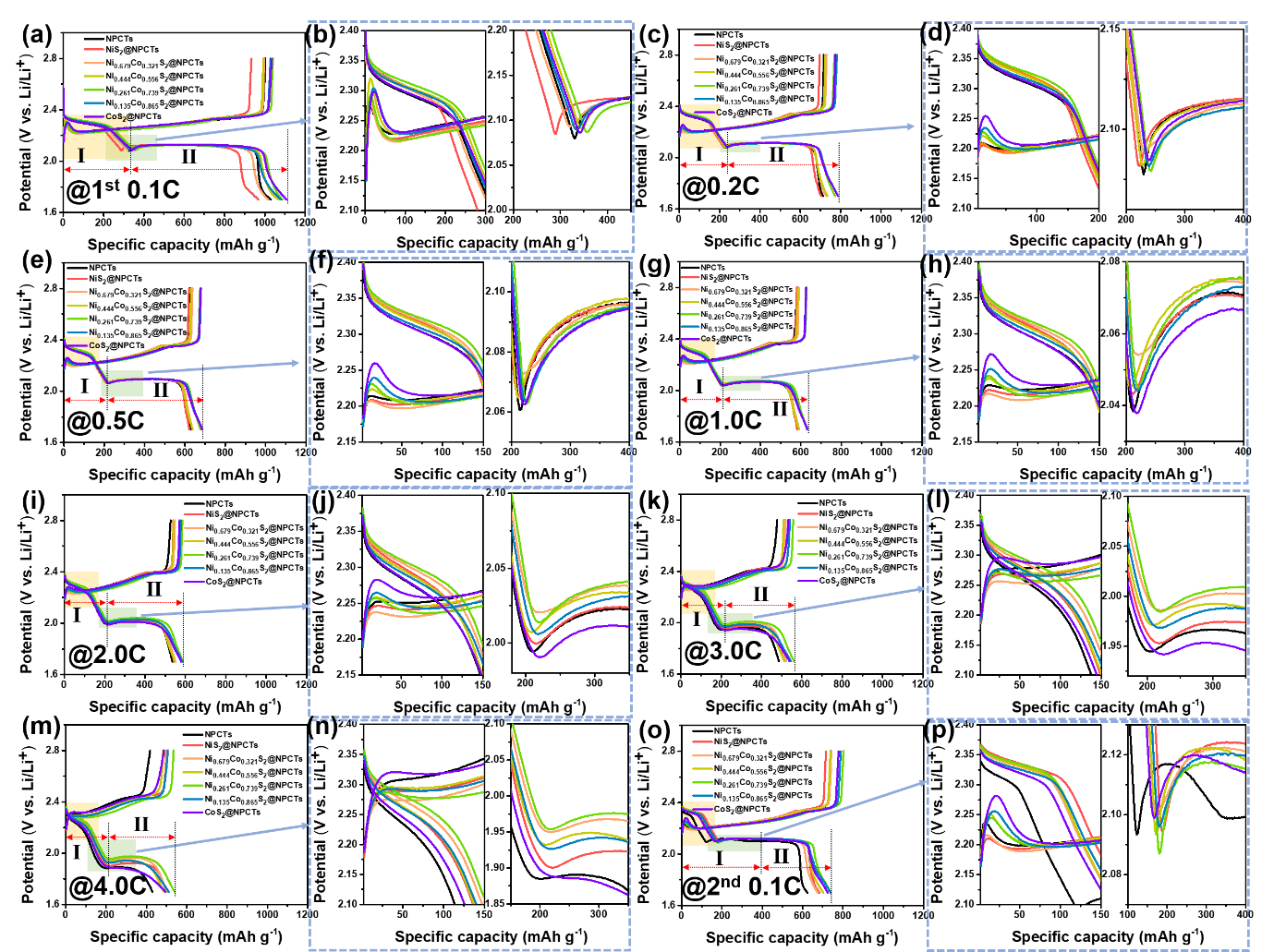


**Figure S16.** Galvanostatic charge-discharge profiles with enlarged areas showing the first discharge plateau and beginning of the charge process (marked as I), and the second discharge plateau (marked as II) at different current rates of (a, b) 1st 0.1 C, (c, d) 0.2 C, (e, f) 0.5 C, (g, h) 1.0 C, (i, j) 2.0 C, (k, l) 3.0 C, (m, n) 4.0 C, and (o, p) 2nd 0.1 C for the NPCTs, and Ni_x_Co_1-x_S_2_@NPCTs cathodes.


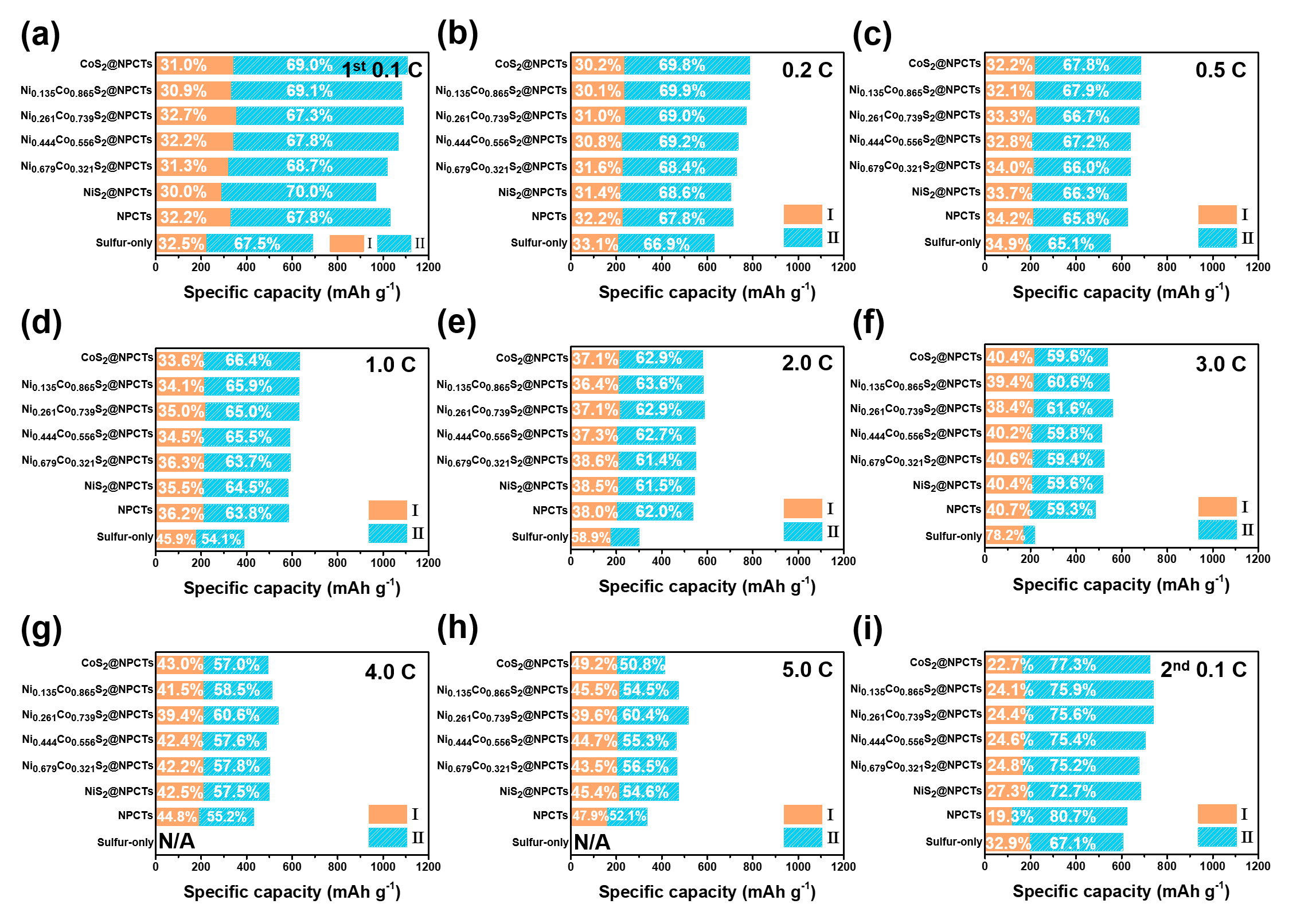


**Figure S17.** Discharge capacity ratios from the first plateau (denoted as I) and second plateau (denoted as II) at different current rates of (a) 1st 0.1 C, (b) 0.2 C, (c) 0.5 C, (d) 1.0 C, (e) 2.0 C, (f) 3.0 C, (g) 4.0 C, (h) 5.0 C, and (i) 2nd 0.1 C for the sulfur-only, NPCTs, and Ni_x_Co_1-x_S_2_@NPCTs cathodes.


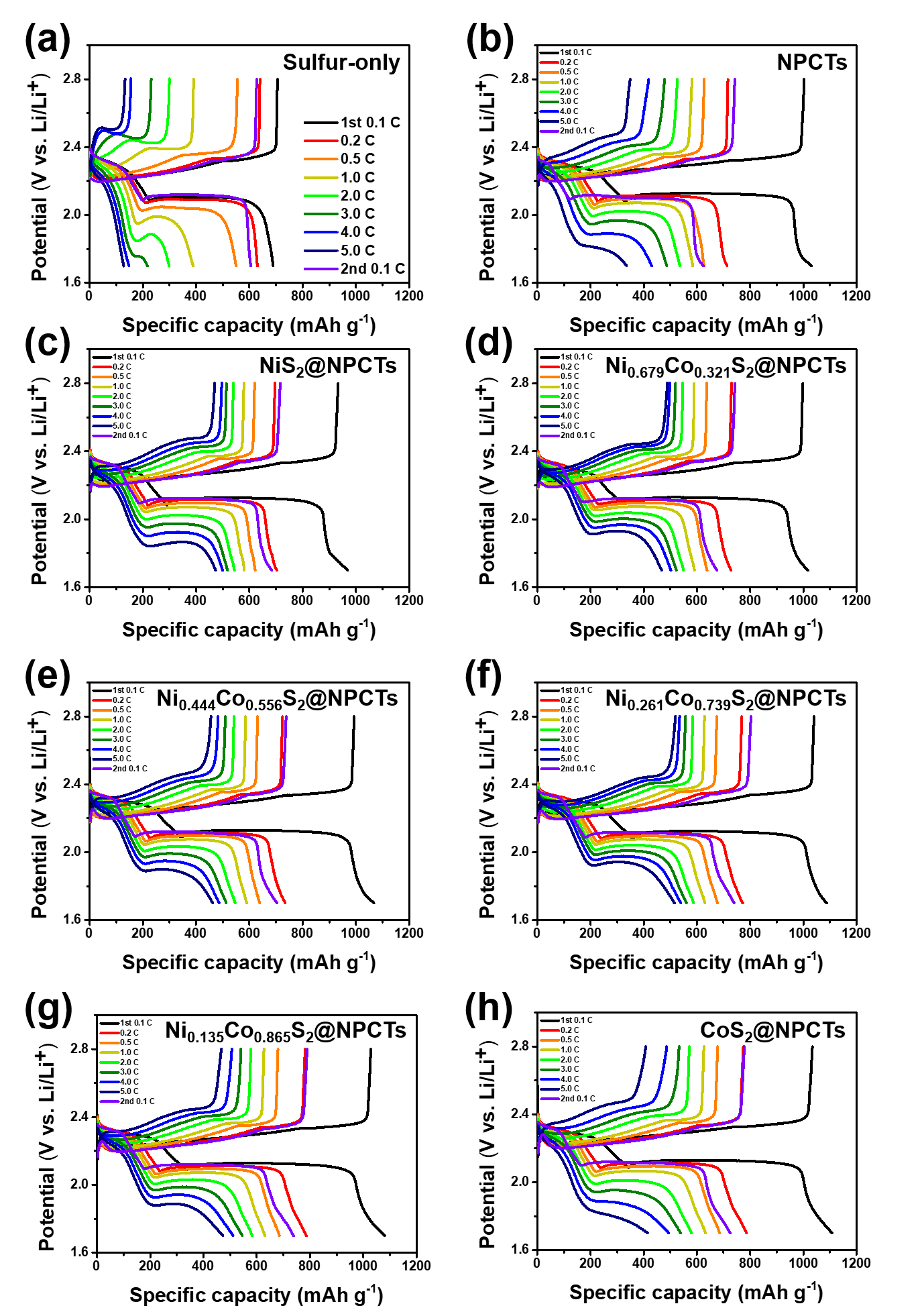


**Figure S18.** Galvanostatic charge-discharge profiles at different current rates for the (a) sulfur-only, (b) NPCTs, (c) NiS_2_@NPCTs, (d) Ni_0.679_Co_0.321_S_2_@NPCTs, (e) Ni_0.444_Co_0.556_S_2_@NPCTs, (f) Ni_0.261_Co_0.739_S_2_@NPCTs, (g) Ni_0.135_Co_0.865_S_2_@NPCTs, and (h) CoS_2_@NPCTs cathodes.


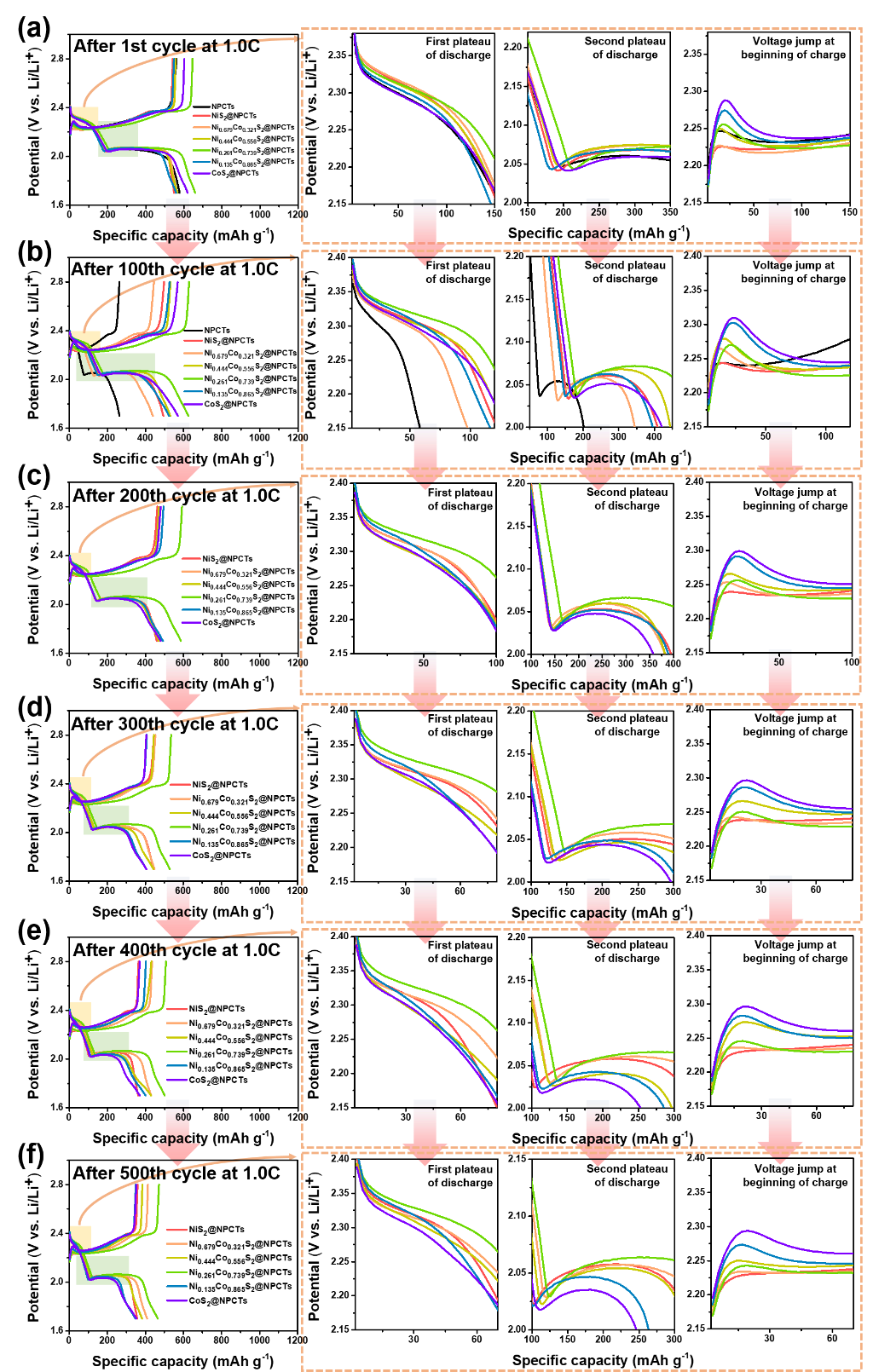


**Figure S19.** Galvanostatic charge-discharge profiles of the Ni_x_Co_1-x_S_2_@NPCTs cathodes after the (a) 1st, (b) 100th, (c) 200th, (d) 300th, (e) 400th, and (f) 500th cycle at 1.0 C rate with enlarged areas of the first and second discharge plateaus, and beginning part of charge.


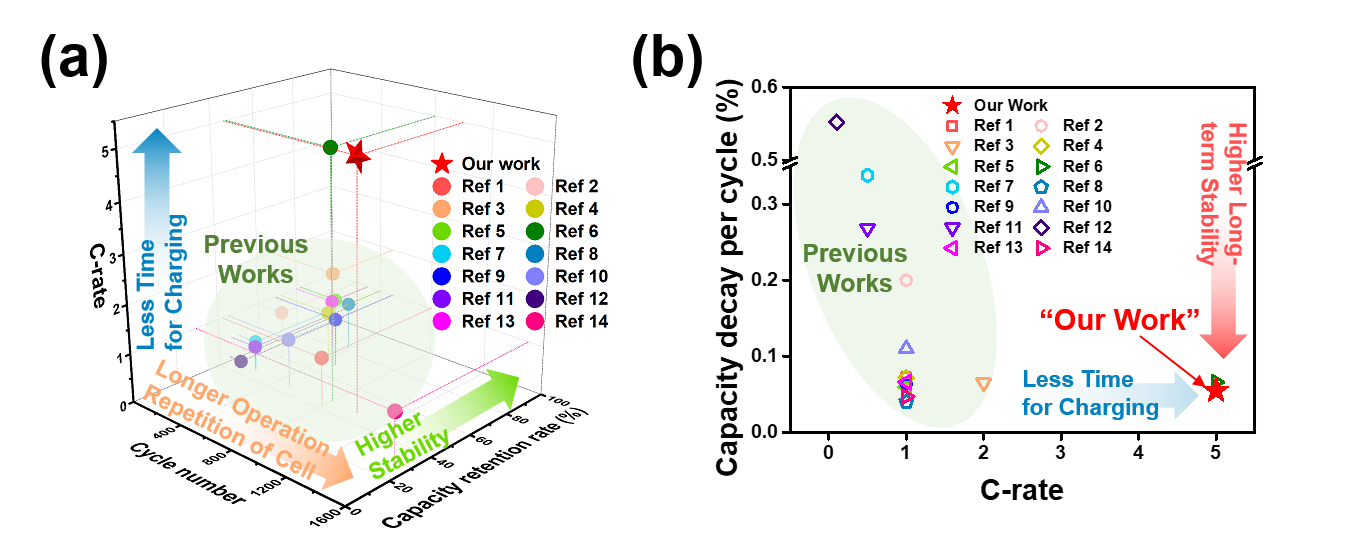


**Figure S20**. Comparison of electrochemical performances measured in this work with those of previously reported host cathode materials of Li-S batteries using similar metal sulfide electrocatalysts, expressed in terms of (a) C-rate versus cycle number and capacity retention rate (%), and (b) C-rate versus capacity decay per cycle (%).

*
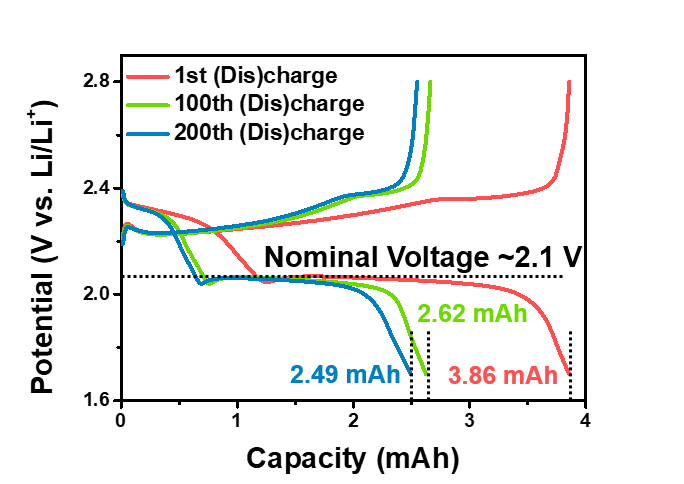
*

**Figure S21.** Galvanostatic charge-discharge profiles showing the 1st, 100th, and 200th cycle processes obtained from the electrochemical performance of the highy sulfur loaded (4.61 mg cm^-2^) Ni_0.261_Co_0.739_S_2_@NPCTs cathode at 0.2 C (Figure 3h).


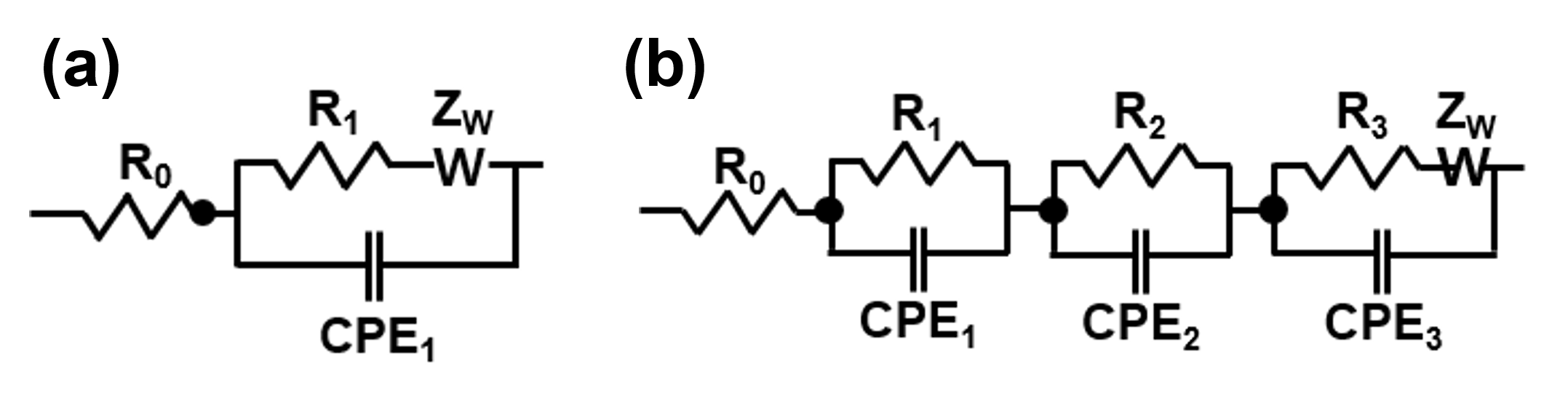


**Figure S22**. Equivalent circuit diagrams for ex-situ EIS analysis of the samples collected at (a) pristine state, (b) 1-cycled state and 500-cycled state. All of experimental cycling performances before the EIS analysis were obtained at 1.0 C rate condition.


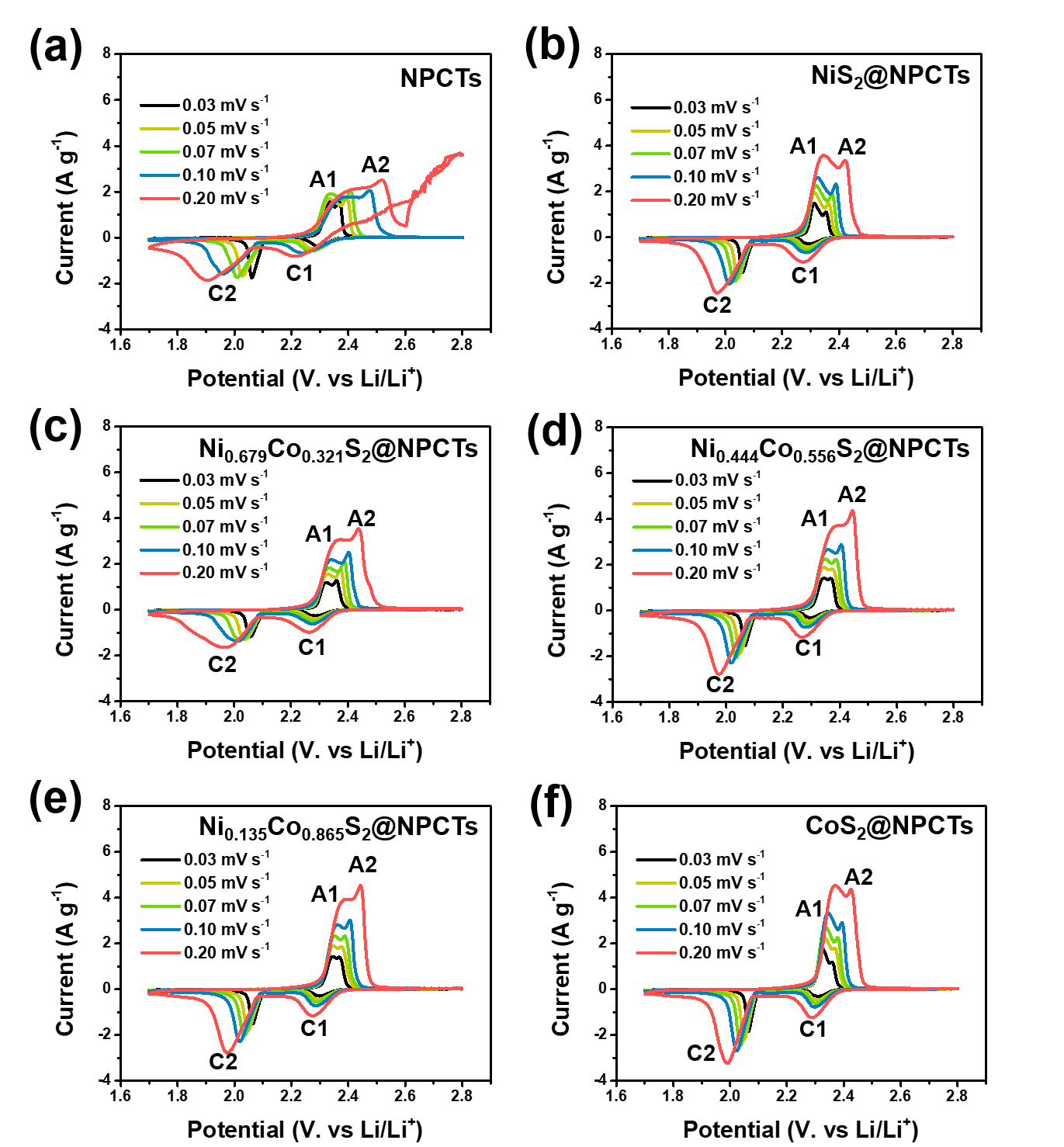


**Figure S23.** CV curves of the asymmetric (a) NPCTs, (b) NiS_2_@NPCTs, (c) Ni_0.679_Co_0.321_S_2_@NPCTs, (d) Ni_0.444_Co_0.556_S_2_@NPCTs, (e) Ni_0.135_Co_0.865_S_2_@NPCTs, and (f) CoS_2_@NPCTs cathodes at various rates of 0.03 - 0.20 mV s^-1^ including the cathodic/anodic redox peaks specified as C1, C2, A1, and A2, respectively.


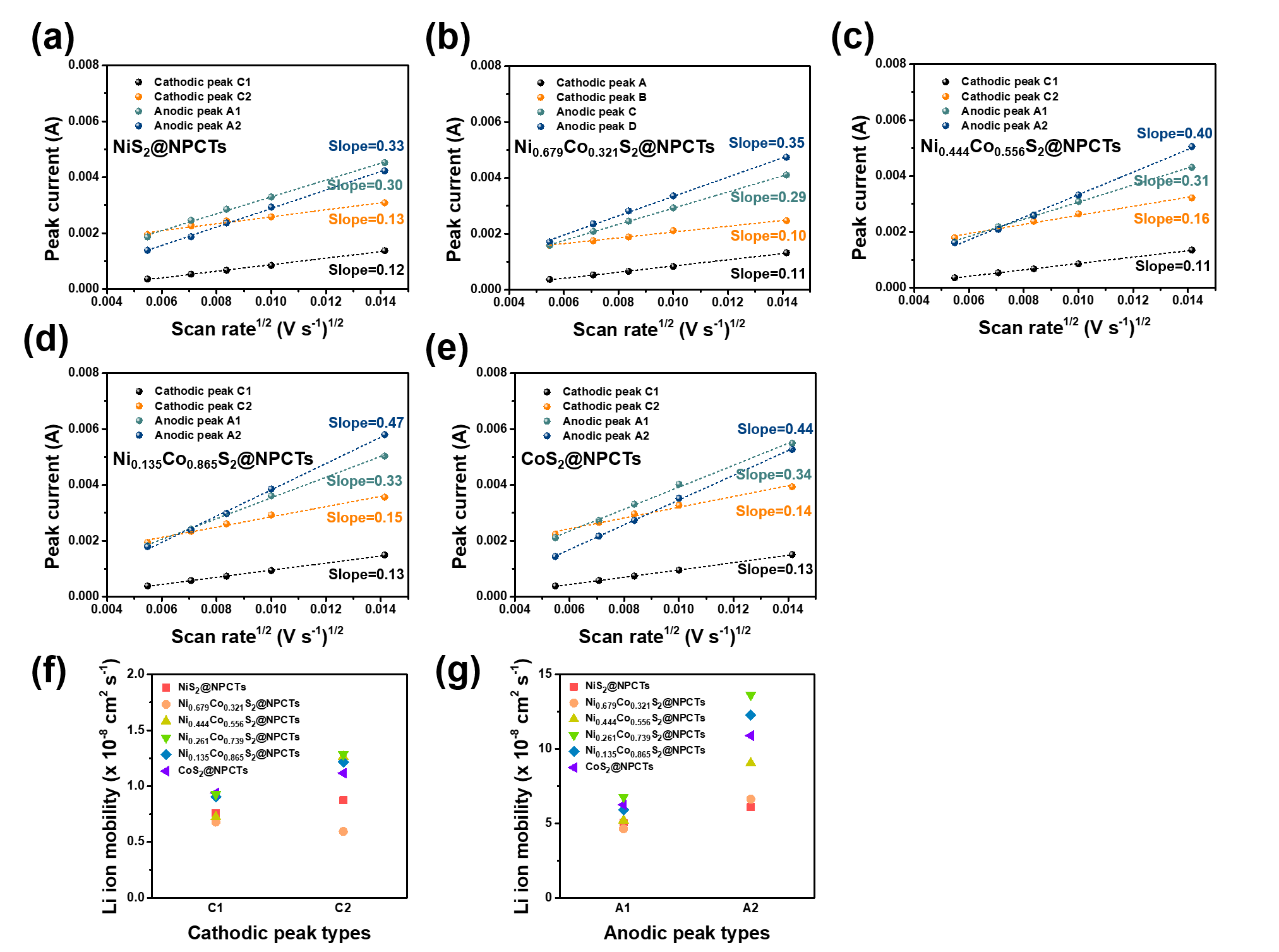


**Figure S24.** (a-e) The fitted lines showing linear relationship between cathodic/anodic redox peak currents versus the square root of the scan rate and (f, g) calculated lithium ion mobility values of the Ni_x_Co_1-x_S_2_@NPCTs cathodes. The lithium ion diffusion coefficient (*D*_Li+_) for ion mobility could be calculated by using the following Randles-Sevick equation:

$i_{p}=2.69\times{10}^{5}n^{1.5}{AD}_{Li+}^{0.5}C_{Li+}v^{0.5}$ (S7)

, where *i*_p_, *n*, *A*, *D*_Li+_, and *C*_Li+_ are the peak current, electron transfer number, area of the electrode (cm^2^), lithium ion diffusion coefficient (cm^2^ s^-1^), Li concentration in the electrolyte (mM), and scan rate (V s^-1^), respectively.


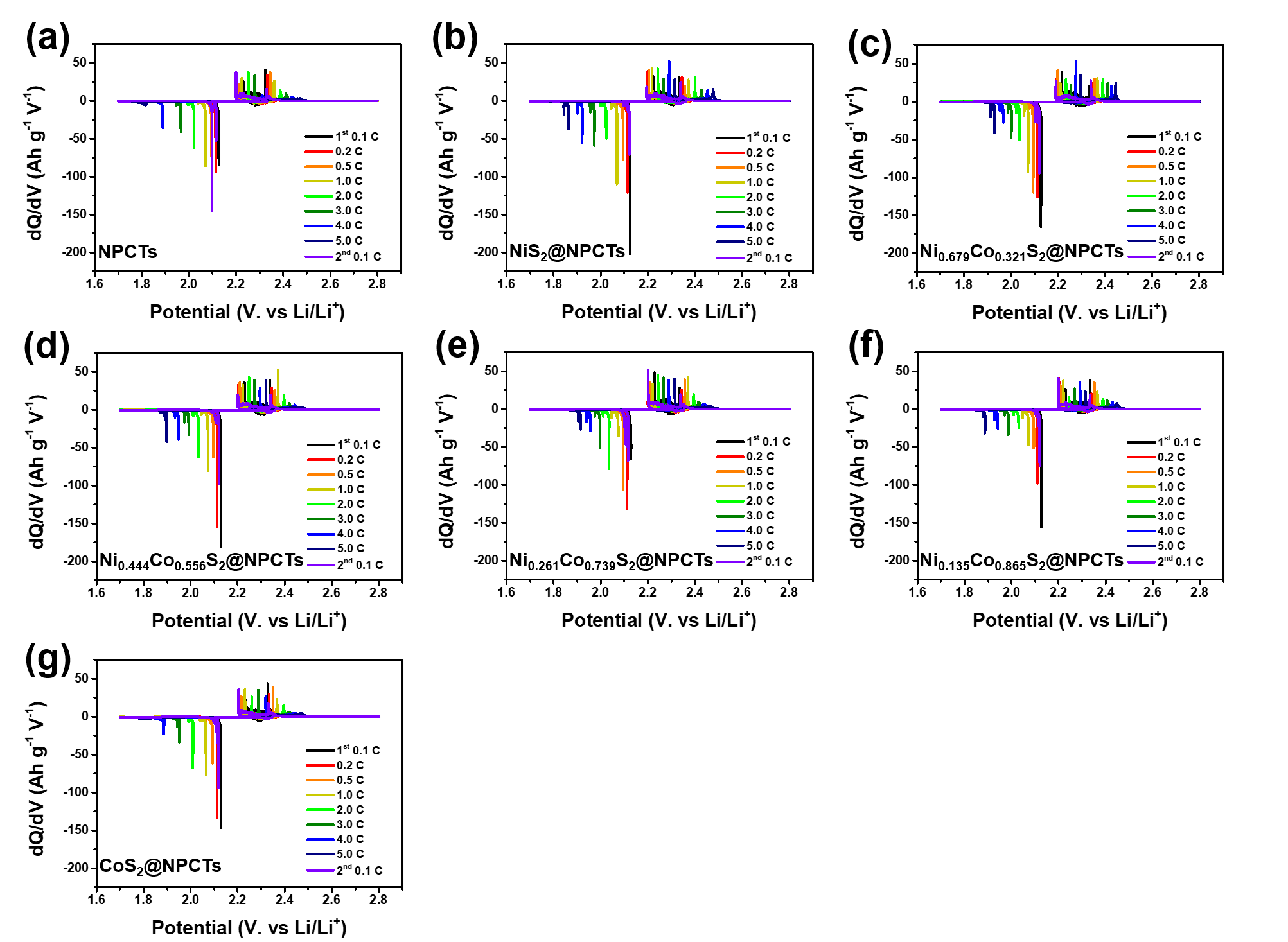


**Figure S25.** dQ/dV curves based on the initial charge-discharge profiles at different C-rates for the (a) NPCTs, (b) NiS_2_@NPCTs, (c) Ni_0.679_Co_0.321_S_2_@NPCTs, (d) Ni_0.444_Co_0.556_S_2_@NPCTs, (e) Ni_0.261_Co_0.739_S_2_@NPCTs, (f) Ni_0.135_Co_0.865_S_2_@NPCTs, and (g) CoS_2_@NPCTs cathodes.


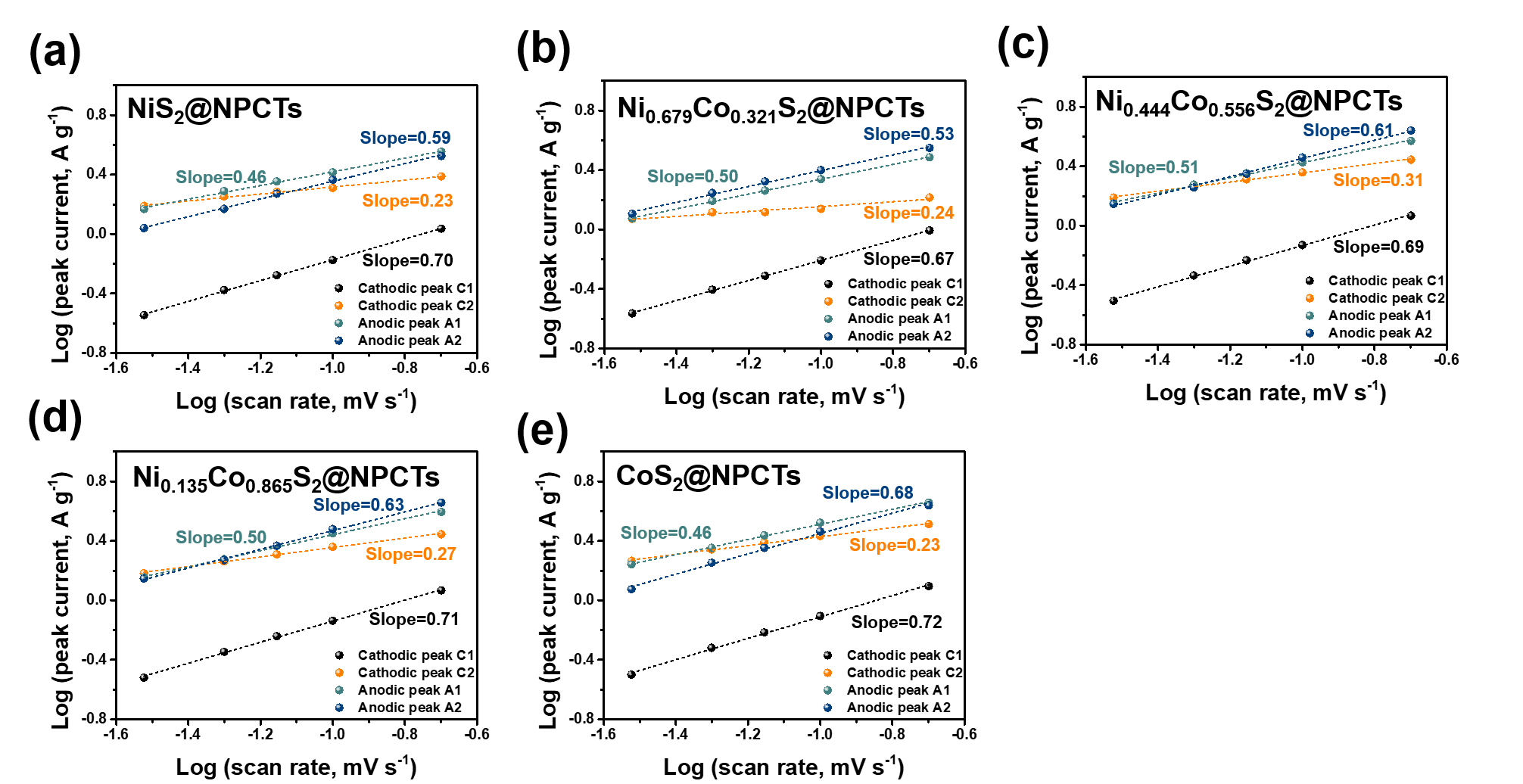


**Figure S26.** The fitted lines showing linear relationship between peak currents versus scan rate in log-log scale at the reduction and oxidation processes for the (a) NiS_2_@NPCTs, (b) Ni_0.679_Co_0.321_S_2_@NPCTs, (c) Ni_0.444_Co_0.556_S_2_@NPCTs, (d) Ni_0.135_Co_0.865_S_2_@NPCTs, and (e) CoS_2_@NPCTs cathodes. Contributions of capacitive- or diffusion-controlled process could be determined by qualitative calculation with the following equations:

$\log i=a+b\log v$ (S8)

$i=k_{1}v+k_{2}v^{1/2}$ (S9)

, where *i* represents the current response at a particular scan rate *v*, a and b are adjustable constants derived from the intercept and the slope of the linear fit plot of log(*v*) vs. log(*i*). In general, when the b value is close to 0.5, it indicates the electrochemical system is diffusion-controlled, while when approaches to 1, it reveals the process is a totally capacitive-controlled. In this regard, the contributions of capacitive-controlled process (*k*_1_*v*) and diffusion-controlled electrochemical reaction (*k*_2_*v^1/2^*) could be quantitatively determined.


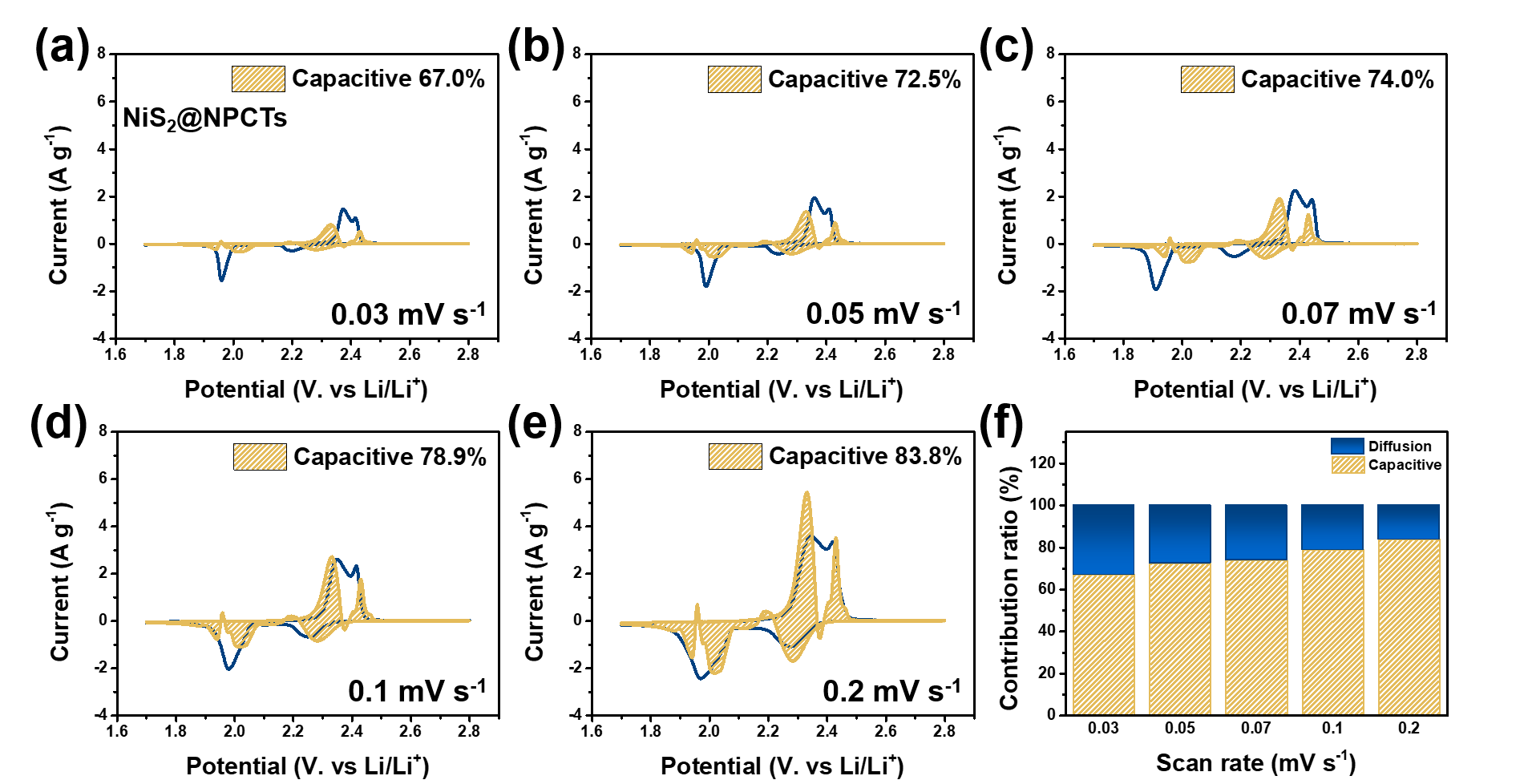


**Figure S27.** (a-e) CV profiles of the NiS_2_@NPCTs with capacitive-controlled contribution to the charge storage at various scan rates of 0.03 - 0.20 mV s^-1^, and (f) bar graphs showing the corresponding contribution ratios between capacitive- and diffusion-controlled processes.


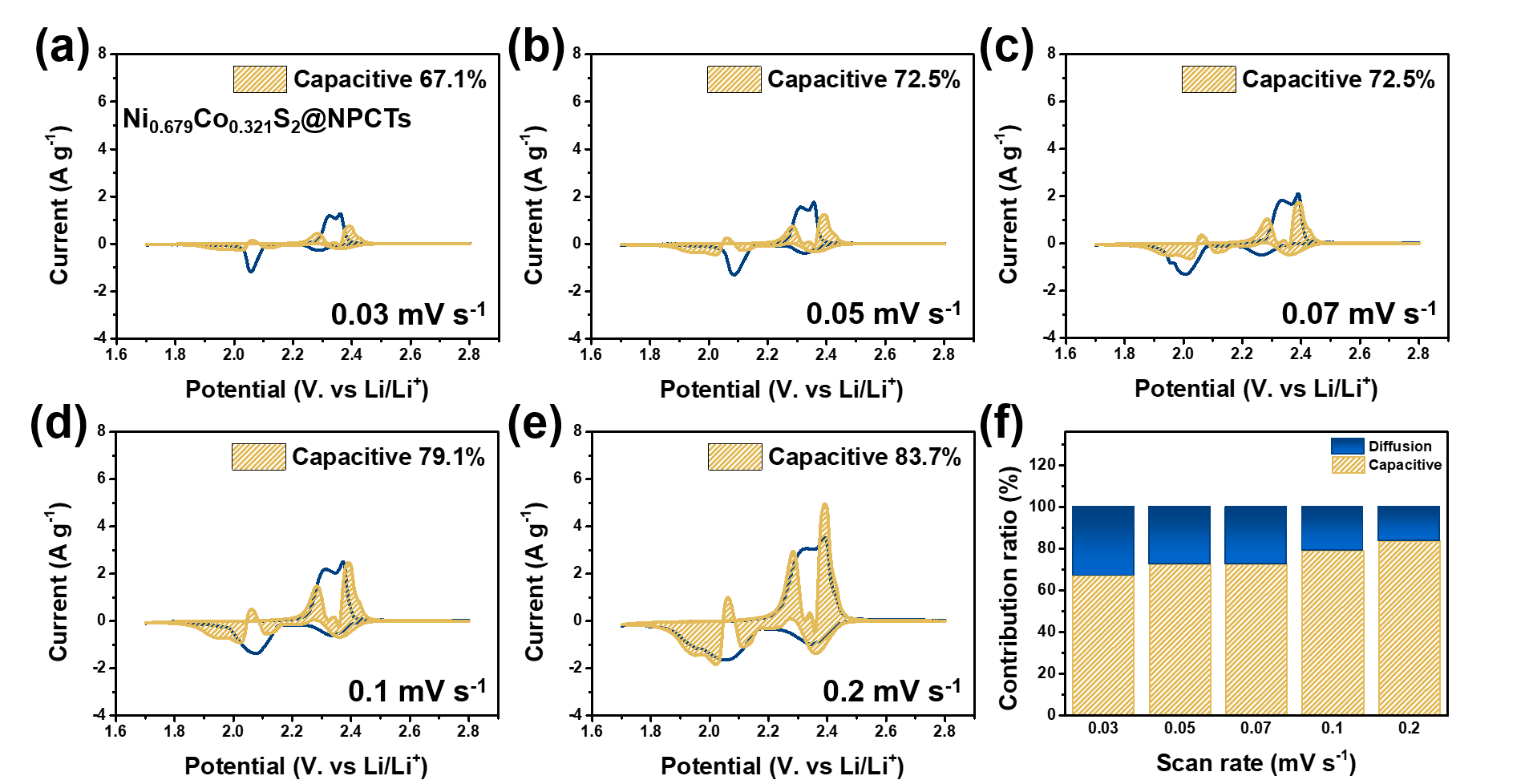


**Figure S28.** (a-e) CV profiles of Ni_0.679_Co_0.321_S_2_@NPCTs with capacitive-controlled contribution to the charge storage at various scan rates of 0.03 - 0.20 mV s^-1^, and (f) bar graphs showing the corresponding contribution ratios between capacitive- and diffusion-controlled processes.


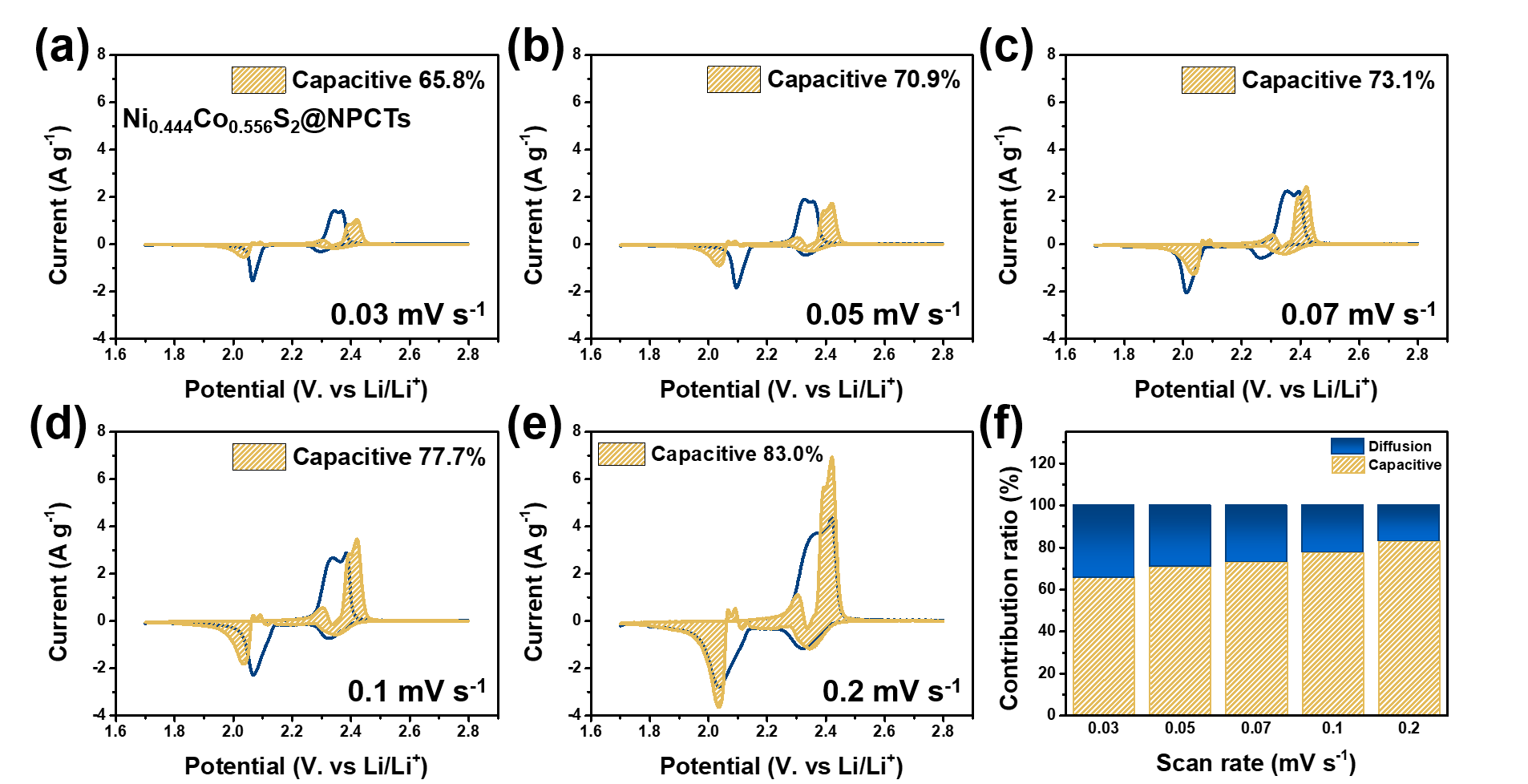


**Figure S29.** (a-e) CV profiles of Ni_0.444_Co_0.556_S_2_@NPCTs with capacitive-controlled contribution to the charge storage at various scan rates of 0.03 - 0.20 mV s^-1^, and (f) bar graphs showing the corresponding contribution ratios between capacitive- and diffusion-controlled processes.


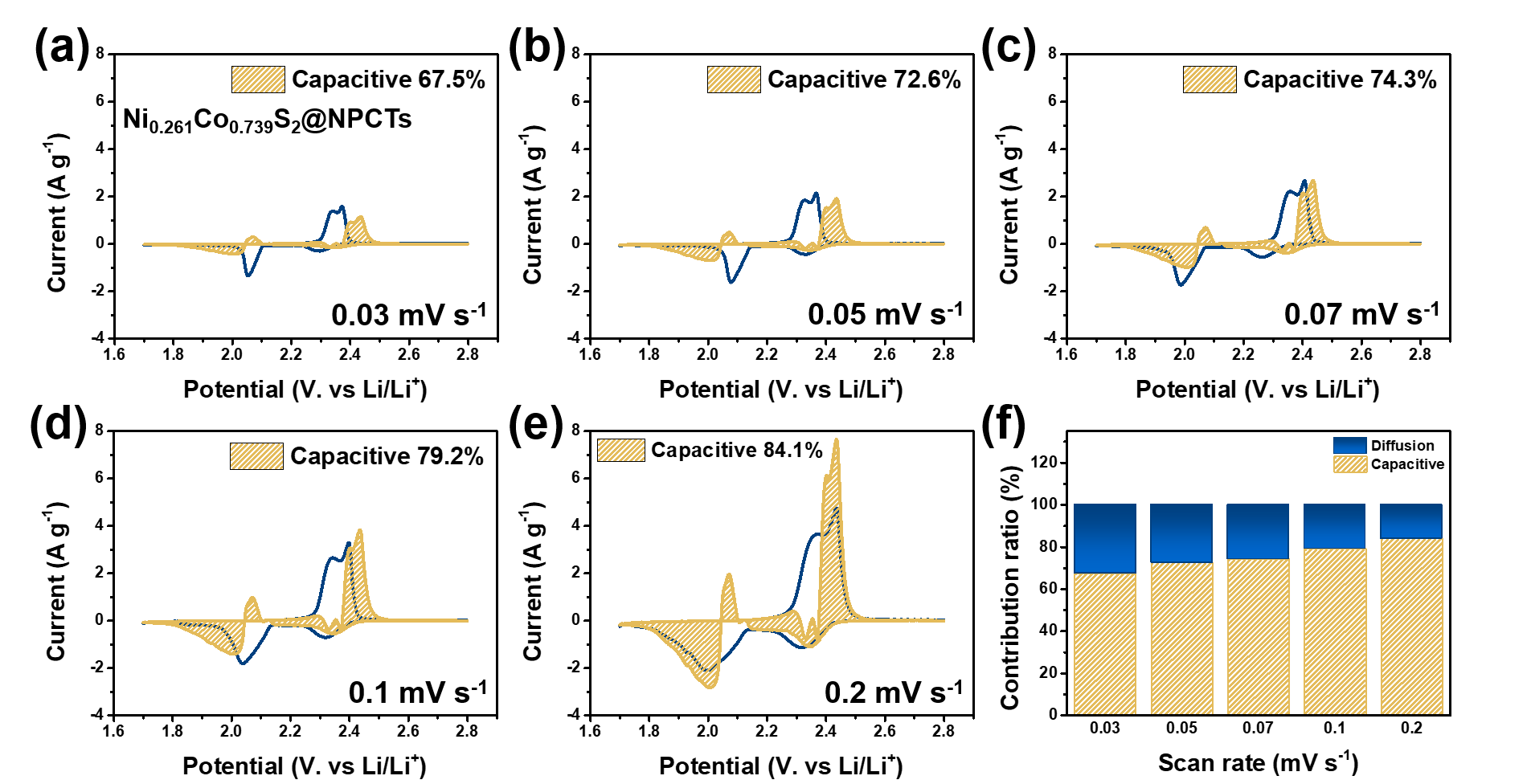


**Figure S30.** (a-e) CV profiles of Ni_0.261_Co_0.739_S_2_@NPCTs with capacitive-controlled contribution to the charge storage at various scan rates of 0.03 - 0.20 mV s^-1^, and (f) bar graphs showing the corresponding contribution ratios between capacitive- and diffusion-controlled processes.


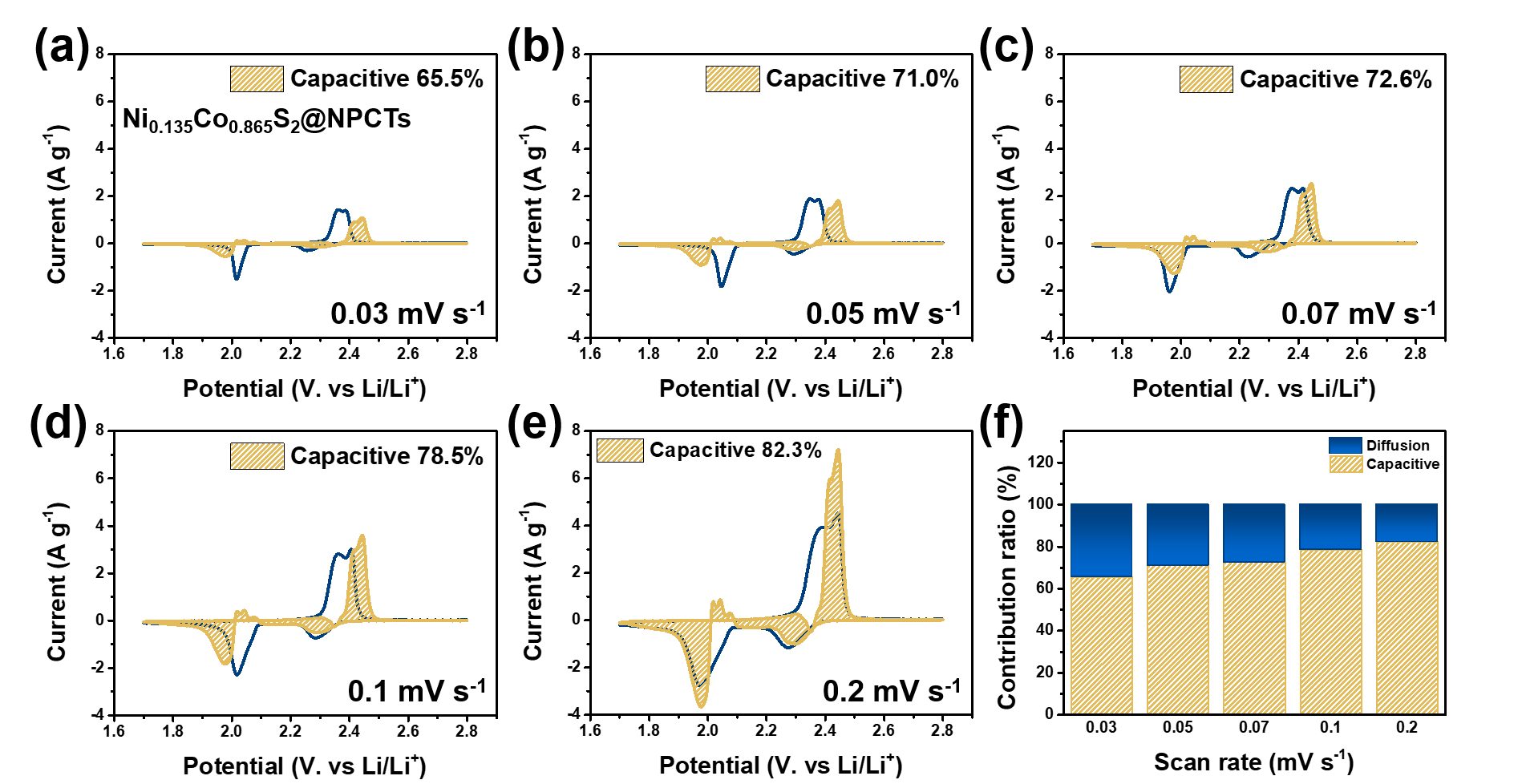


**Figure S31.** (a-e) CV profiles of Ni_0.135_Co_0.865_S_2_@NPCTs with capacitive-controlled contribution to the charge storage at various scan rates of 0.03 - 0.20 mV s^-1^, and (f) bar graphs showing the corresponding contribution ratios between capacitive- and diffusion-controlled processes.


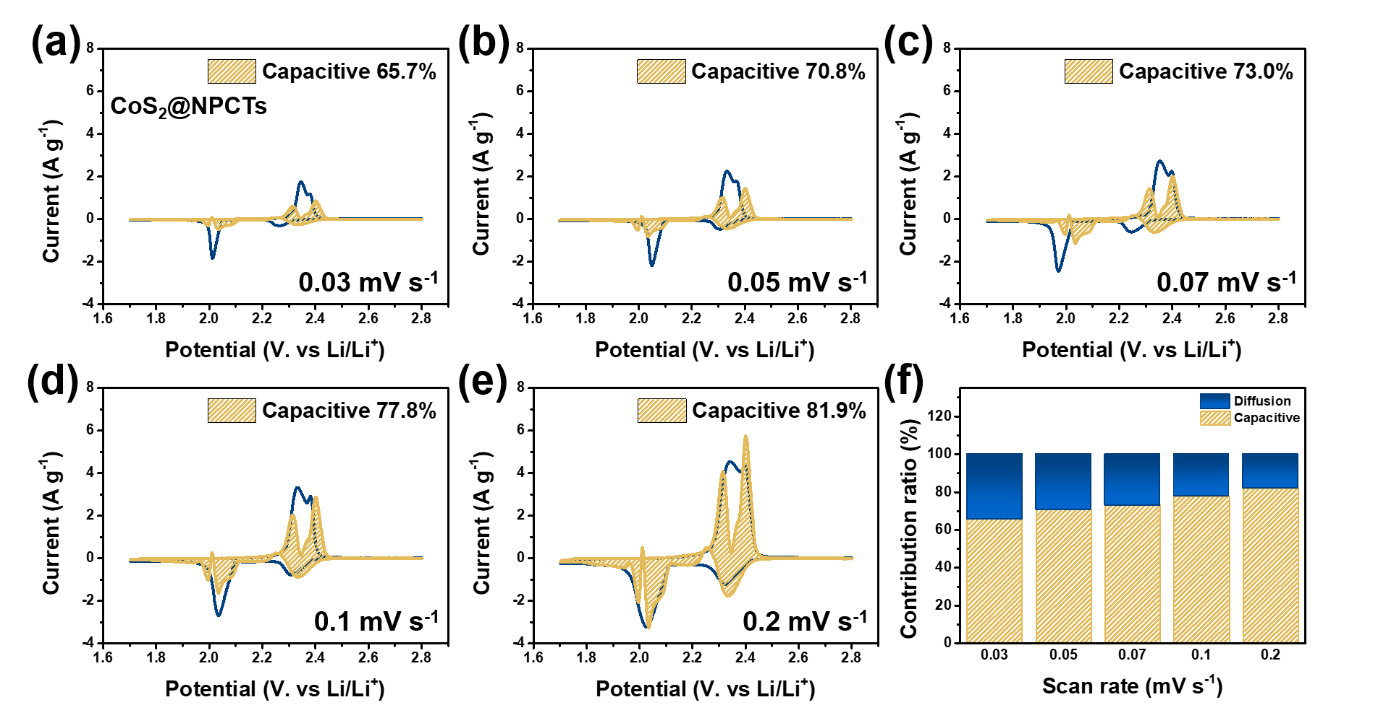


**Figure S32.** (a-e) CV profiles of CoS_2_@NPCTs with capacitive-controlled contribution to the charge storage at various scan rates of 0.03 - 0.20 mV s^-1^, and (f) bar graphs showing the corresponding contribution ratios between capacitive- and diffusion-controlled processes.


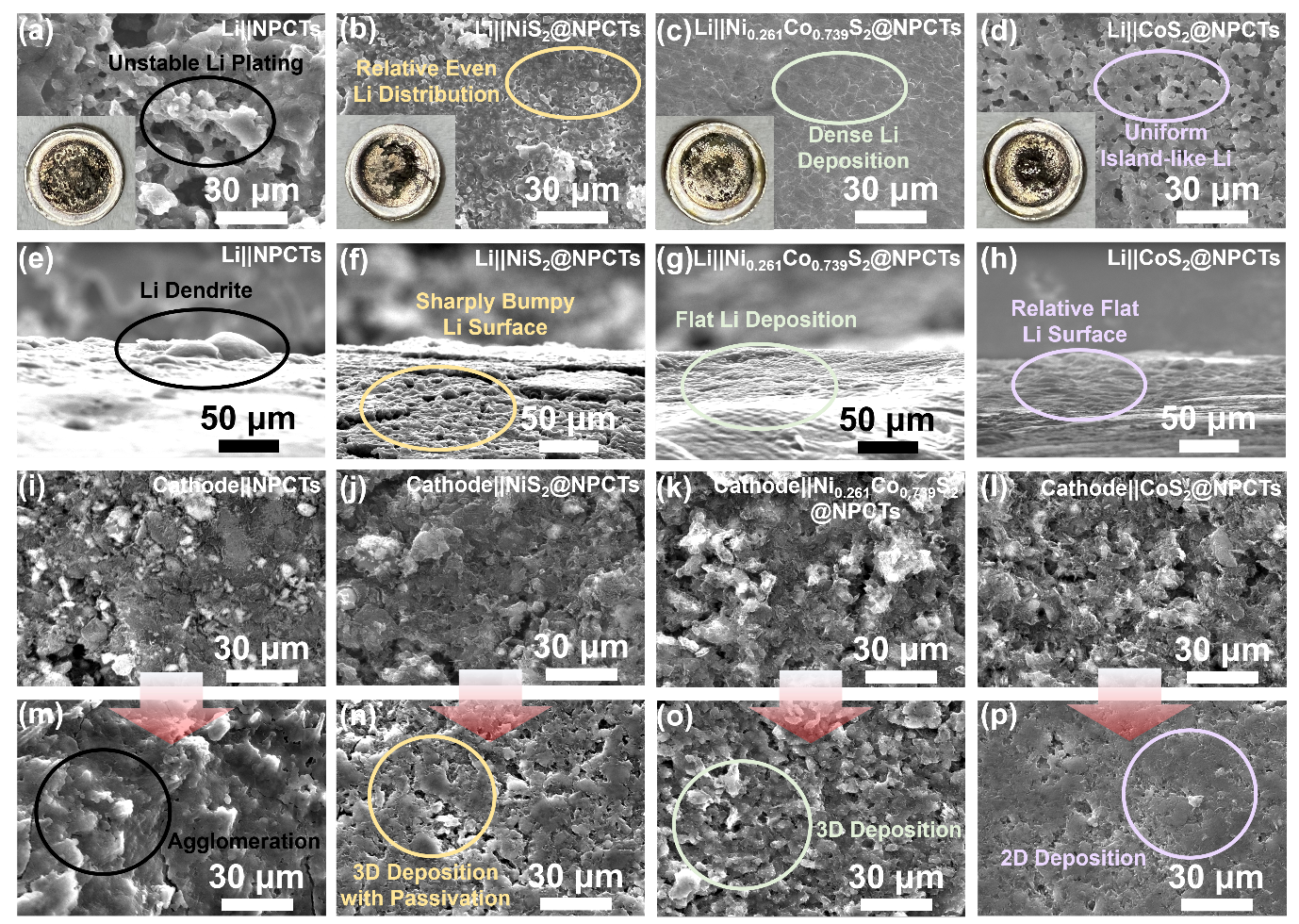


**Figure S33.** Post-mortem analysis of the lithium anodes and the Ni_0.261_Co_0.739_S_2_@NPCTs cathodes after long-term 500 cycling performances at 1.0 C rate. Ex-situ SEM images of the lithium anodes of (a-d) top-view and (e-h) cross-sectional view. Ex-situ SEM images of the cathodes in (j-l) pristine state and (m-p) 500-cylced state.


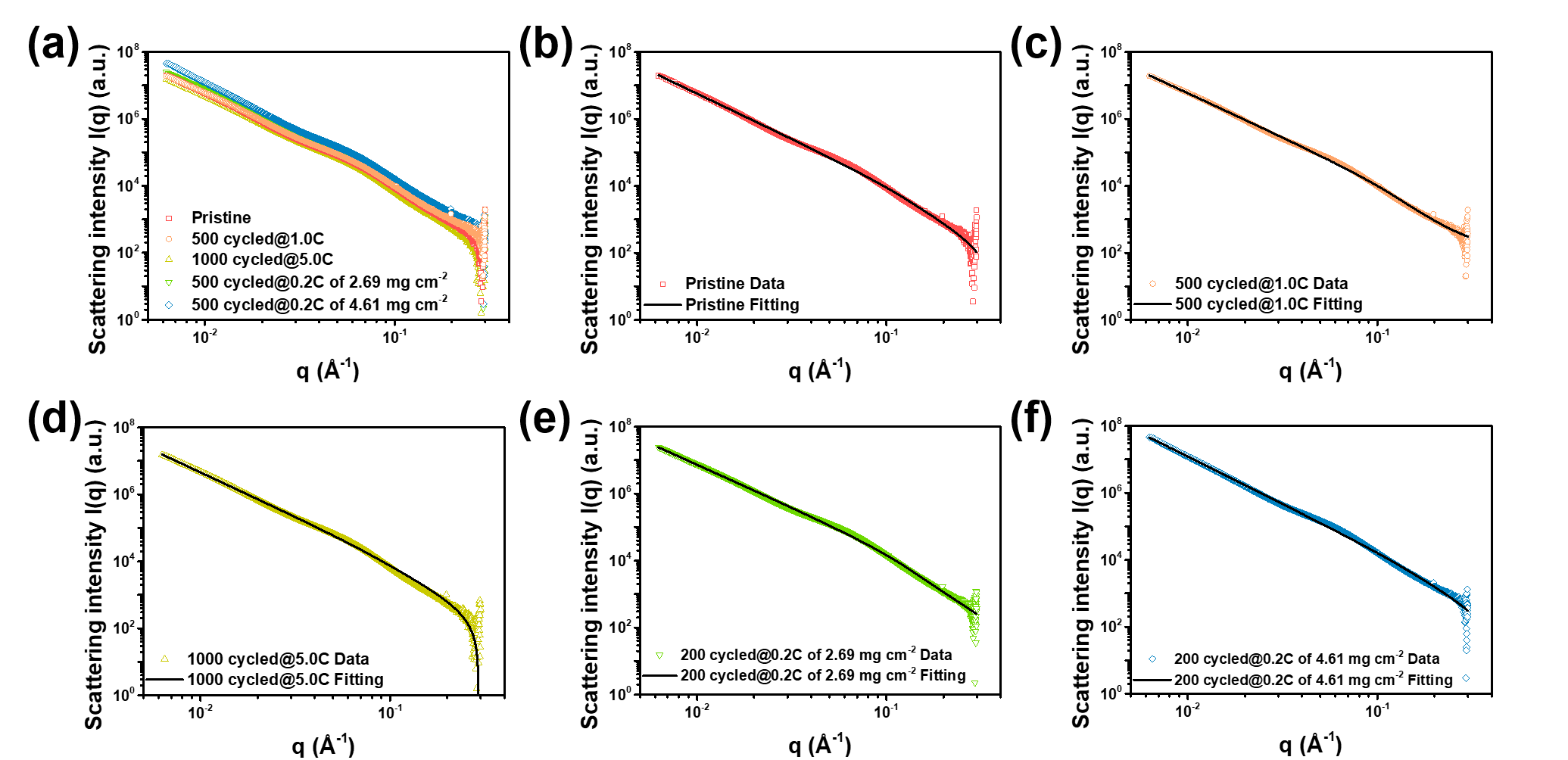


**Figure S34.** (a) Ex-situ powder SAXS analysis of the Ni_0.261_Co_0.739_S_2_@NPCTs cathodes after long-term cycling performances. Guinier-Porod model evaluation was applied to the various Ni_0.261_Co_0.739_S_2_@NPCTs samples in (b) pristine, (c) 500 cycled at 1.0 C rate, (d) 1000 cycled at 5.0 C rate, and (e, f) 200 cycled at 0.2 C rate with sulfur loadings of 2.69 mg cm^-2^ and 4.61 mg cm^-2^ states, respectively.


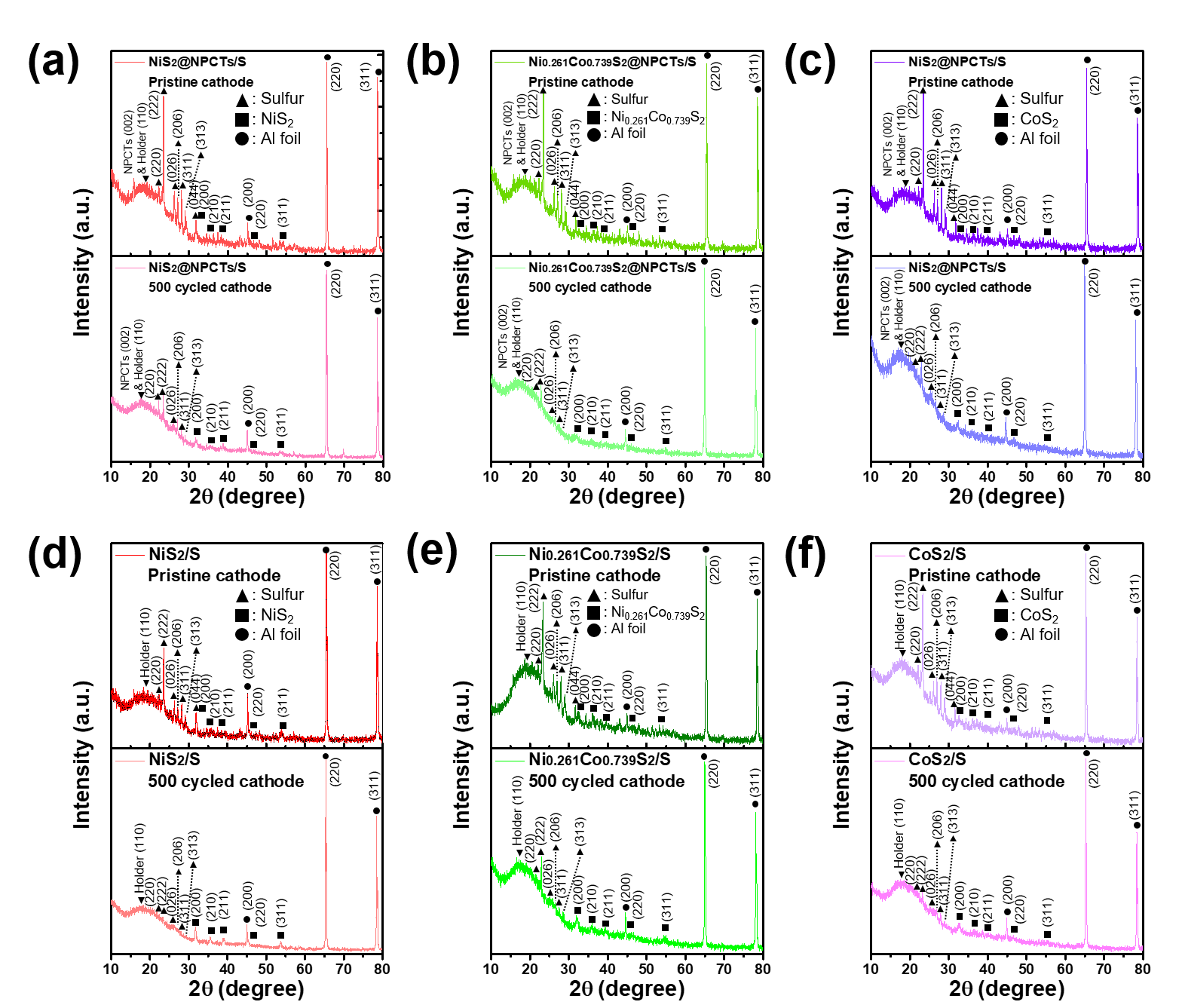


**Figure S35.** Ex-situ XRD analysis of the cathodes after long-term 500 cycles at 1.0 C rate compared with the pristine states for the (a) NiS_2_@NPCTs/S, (b) Ni_0.261_Co_0.739_S_2_@NPCTs/S, (c) CoS_2_@NPCTs/S, (d) NiS_2_/S, (e) Ni_0.261_Co_0.739_S_2_/S, and (f) CoS_2_/S electrodes.


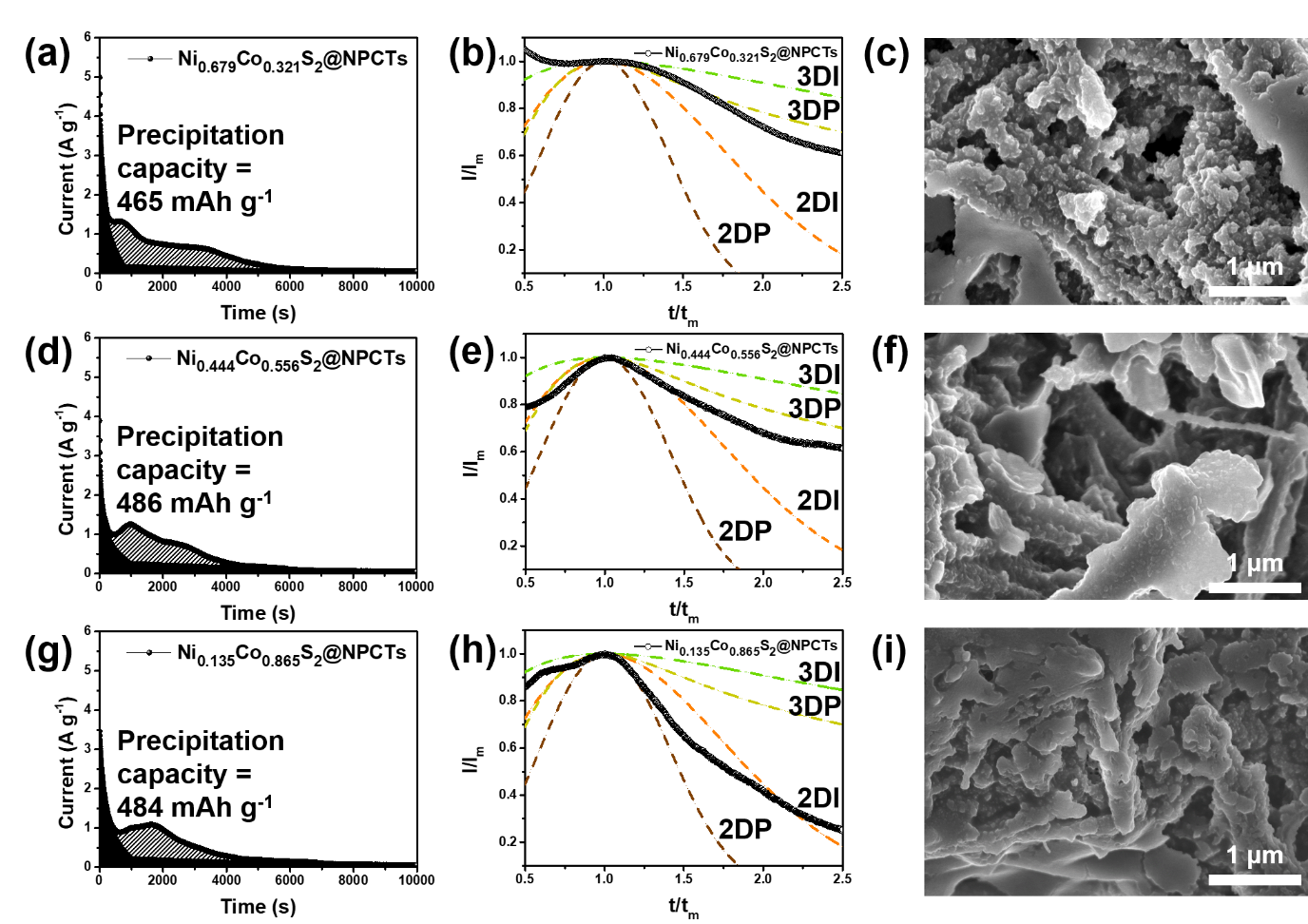


**Figure S36.** Potentiostatic discharge profiles of Li_2_S_6_ solution at 2.03 V (vs. Li/Li^+^) showing Li_2_S precipitation capacity, dimensionless *i*-*t* curves for Li_2_S nucleation mechanism, and corresponding ex-situ SEM images of the Li_2_S-grown cathode catalysts for the (a-c) Ni_0.679_Co_0.321_S_2_@NPCTs, (d-f) Ni_0.444_Co_0.556_S_2_@NPCTs, and (g-i) Ni_0.261_Co_0.739_S_2_@NPCTs. The detail methods for the analyses of Li_2_S nucleation on the cathode catalysts in this work are summarized in the next section.

**Analysis of Li_2_S nucleation and growth on the cathode substrates**

The dimensionless current-time transient profiles were obtained by using maximum points of current (*I*_m_) and specific time (*t*_m_) for quantitative peak fitting. Specifically, Bewick, Fleischman, and Thirsk (BFT) model and Scharifker-Hills (SH) model are applied to fit the 2D and 3D Li_2_S nucleation mechanisms, respectively, via theoretical expressions of the current-time transients of chronoamperometry including the four electrochemical deposition models: (1) 2D instantaneous growth (2DI), (2) 2D progressive growth (2DP), (3) 3D instantaneous growth (3DI), and (4) 3D progressive growth (3DP). The 2D models mean the merging of adjacent atoms into the lattice interface which induces a planar deposition layer (Equations S10-S14), and the 3D models mean the growth by three-dimensional volumetric diffusion controlled process (Equations S15-S20).

1. 2D nucleation: A. Bewick, M. Fleischman, and H. R. Thirsk (BFT) model

| Equations | Instantaneous nucleation (2DI) | Progressive nucleation (2DP) |
| --- | --- | --- |
| (S10) | $I_{2DI}\left( t \right)=\frac{2\pi zFMhN_{0}k_{g}^{2}}{\rho}exp\left( -\frac{\pi M^{2}N_{0}k_{g}^{2}}{\rho^{2}}t^{2} \right)$ | $I_{2DP}\left( t \right)=\frac{\pi zFMhAN_{0}k_{g}^{2}}{\rho}exp\left( -\frac{\pi M^{2}{AN}_{0}k_{g}^{2}}{{3\rho}^{2}}t^{3} \right)$ |
| (S11) | $\frac{I}{I_{m}}=\frac{t}{t_{m}}exp\left( \frac{t^{2}-t_{m}^{2}}{2t_{m}^{2}} \right)$ | $\frac{I}{I_{m}}=\left( \frac{t}{t_{m}} \right)^{2}exp\left( \frac{-2\left( t^{3}-t_{m}^{3} \right)}{3t_{m}^{3}} \right)$ |
| (S12) | $t_{m}=\left( \frac{\rho^{2}}{2\pi M^{2}N_{0}k_{g}^{2}} \right)^{1/2}$ | $t_{m}=\left( \frac{{2\rho}^{2}}{\pi M^{2}AN_{0}k_{g}^{2}} \right)^{1/3}$ |
| (S13) | $I_{m}=\frac{\left( 2\pi\right)^{1/2}zFhN_{0}^{1/2}k_{g}}{\rho}exp\left( \frac{-1}{2} \right)$ | $I_{m}=zFh\left( \frac{4\pi AN_{0}k_{g}^{2}\rho}{M} \right)^{1/3}exp\left( \frac{-2}{3} \right)$ |
| (S14) | $I_{m}t_{m}=\frac{zF\rho h}{M}exp\left( \frac{-1}{2} \right)$ | $I_{m}t_{m}=\frac{2zF\rho h}{M}exp\left( \frac{-2}{3} \right)$ |

1. 3D nucleation: Scharifker-Hills (SH) model

| Equations | Instantaneous nucleation (3DI) | Progressive nucleation (3DP) |
| --- | --- | --- |
| (S15) | $I_{3DI}\left( t \right)=\frac{zFD_{0}^{1/2}c}{\pi^{1/2}t^{1/2}}\left\{ 1-\exp\left( -N_{0}\pi kD_{0}t \right) \right\}$ | $I_{3DP}\left( t \right)=\frac{zFD_{0}^{1/2}c}{\pi^{1/2}t^{1/2}}\left\{ 1-\exp\left( -\frac{AN_{\infty}\pi k^{'}D_{0}t^{2}}{2} \right) \right\}$ |
| (S16) | $k=\left( \frac{8\pi cM}{\rho} \right)^{1/2}$ | $k^{'}=\frac{4}{3}\left( \frac{8\pi cM}{\rho} \right)^{1/2}$ |
| (S17) | $\frac{I}{I_{m}}=\left( \frac{1.9542}{t/t_{m}} \right)^{1/2}\left\{ 1-exp\left( 1.2564\frac{t}{t_{m}} \right) \right\}$ | $\frac{I}{I_{m}}=\left( \frac{1.2254}{t/t_{m}} \right)^{1/2}\left\{ 1-exp\left( 2.3367\left( \frac{t}{t_{m}} \right)^{2} \right) \right\}$ |
| (S18) | $t_{m}=\frac{1.2564}{N_{0}\pi k}$ | $t_{m}=\left( \frac{4.6733}{AN_{\infty}\pi k^{'}D_{0}} \right)^{1/2}$ |
| (S19) | $I_{m}=0.6382zFD_{0}c\left( kN_{0} \right)^{1/2}$ | $I_{m}=0.4615zFD_{0}^{3/4}c\left( k^{'}AN_{\infty} \right)^{1/4}$ |
| (S20) | $I_{m}^{2}t_{m}=0.1629\left( zFc \right)^{2}D_{0}$ | $I_{m}^{2}t_{m}=0.2598\left( zFc \right)^{2}D_{0}$ |

*zF*: the molar charge transferred during the electrodeposition and dissolution process, where F = 96,485 C mol^-1^

*h*: the layer thickness [cm]

*k*_g_: the nucleus lateral growth-rate constant [mol cm^-2^ s^-1^]

*c*: the molar concentration [mol cm^-3^]

*ρ, M*: the density and molecular weight of Li_2_S, where *M* = 46 g mol^-1^ and *ρ* = 1.66 g cm^-3^

*I*_m_, *t*_m_: corresponding to the point of the maximum current density

*A*: the nucleation rate constant [s^-1^]

*N*_∞_: the number density of active sites

*N*_0_, *AN*_∞_: the density number of isolated centers for 3DI and 3DP, respectively [cm^-2^ and cm^-2^ s^-1^]

*D*_0_: the effective diffusion coefficient [cm^-2^ s^-1^]


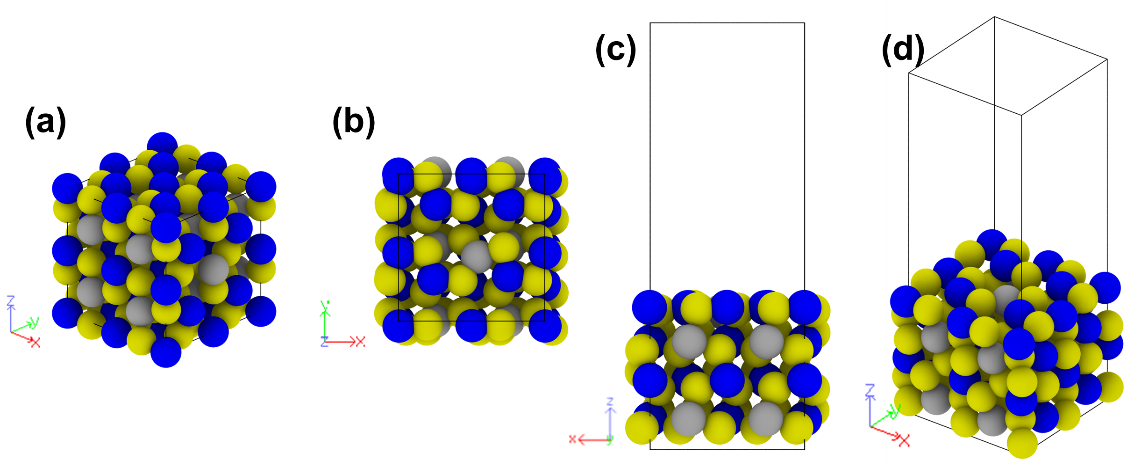


**Figure S37**. (a) Bulk model, and slab models of Ni_0.28125_Co_0.71875_S_2_ (b) top view and (c), (d) side view.


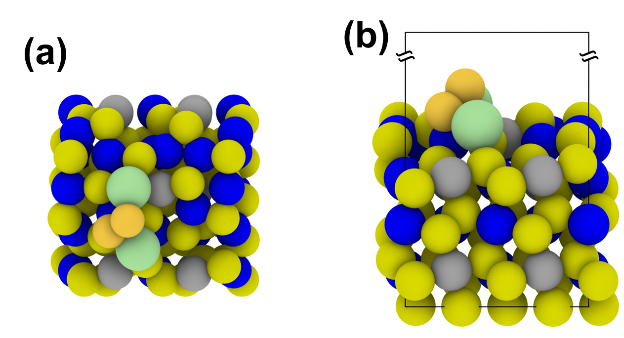


**Figure S38**. Li_2_S_2_ adsorption on Ni_0.28125_Co_0.71875_S_2_ (a) top view and (b) side view.


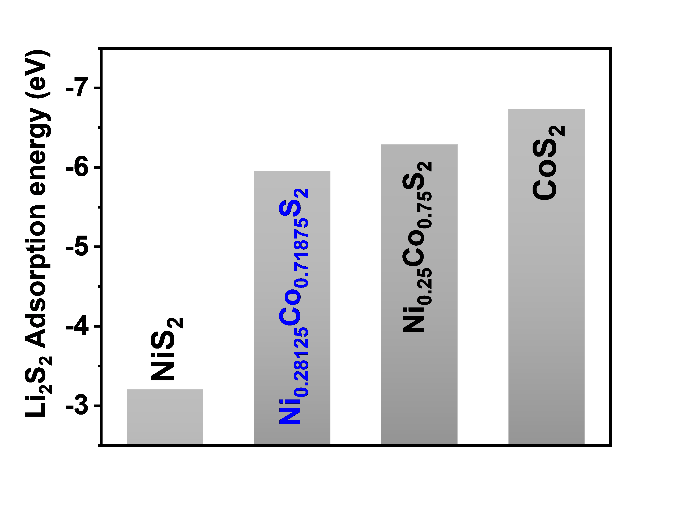


**Figure S39**. Adsorption energy of Li_2_S_2_ on NiS_2_, Ni_0.28125_Co_0.71875_S_2_, Ni_0.25_Co_0.75_S_2_, and CoS_2_.

If the atomic configuration of the Ni_0.261_Co_0.739_S_2_ is strictly identified as Ni:Co=0.261:0.739, approximately 24,000 atoms would be required, which hinder the theoretical process due to limitations of computational costs of QM simulations. If the slab model was designed as Ni:Co=0.28125:0.71875 for Ni_0.28125_Co_0.71875_S_2_ (Figure S37), the adsorption energy of Li_2_S_2_ was calculated as -5.95708 eV which was stronger than that of NiS_2_ and weaker than that of Ni_0.25_Co_0.75_S_2_, consistent with the observed tendency (Figure S38,39). These results indicate that the change of adsorption energy in accordance with the Ni/Co ratio was well-matched with the experimental expectations in this work, and suggest that the more closely the theoretical model approximates reality, the more accurately the desired values can be obtained. In this regard, the calculation conditions were selected as close as possible to the experimental conditions of Ni:Co=0.25:0.75.


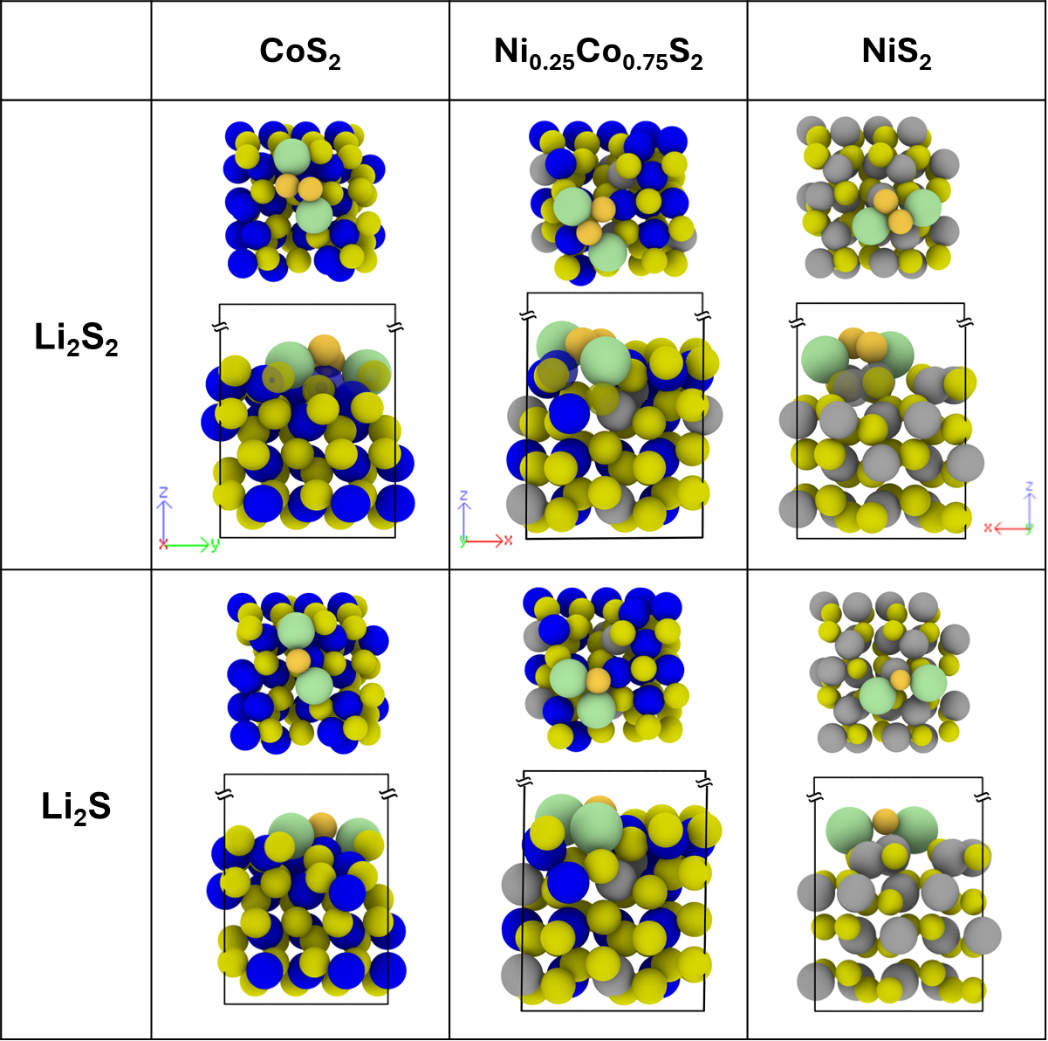


**Figure S40**. Li_2_S_2_ and Li_2_S adsorption on CoS_2_, Ni_0.25_Co_0.75_S_2_, and NiS_2_ fop top and side view


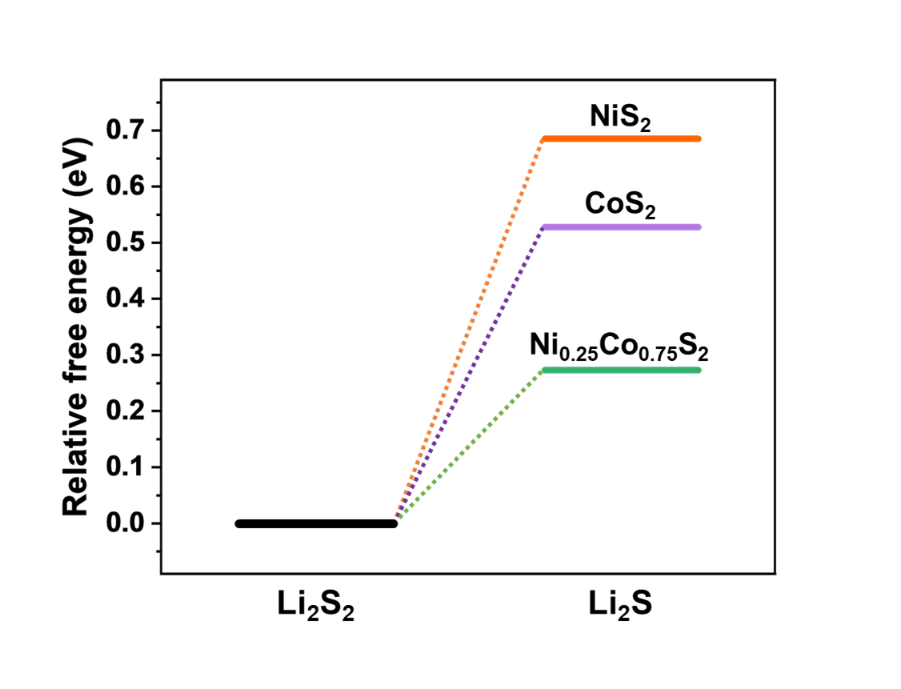


**Figure S41**. Relative free energy diagram from Li_2_S_2_ to Li_2_S for NiS_2_, CoS_2_, and Ni_0.25_Co_0.75_S_2_.


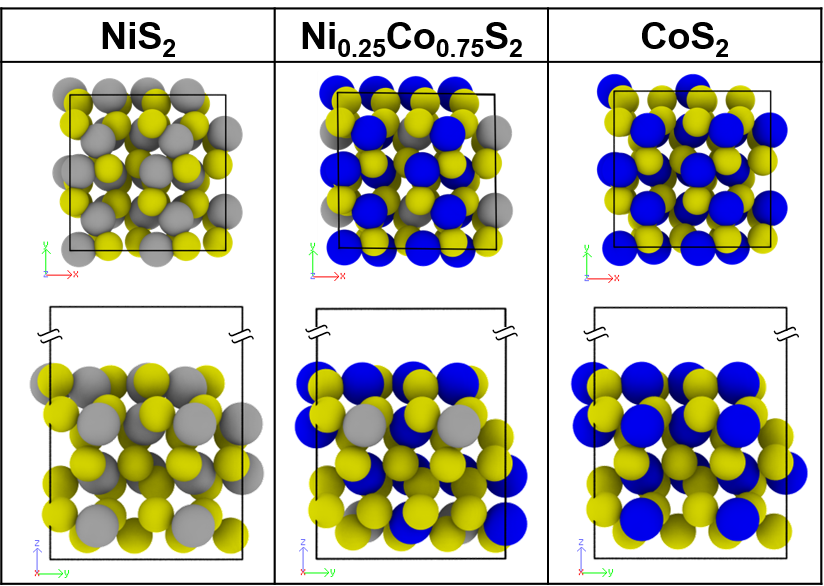


**Figure S42.** Relaxation of all (200) surfaces of NiS_2_, Ni_0.25_Co_0.75_S_2_, and CoS_2_ constructed from relaxed and separated bulk unit cell for computational DFT calculations with a vacuum of 20 Å to avoid interactions between the top and bottom surfaces.

**Table S1.** Estimation of configurations for Ni and Co elements in the Ni_x_Co_1-x_S_2_ composites by ICP-AES analysis.

| Ni(TU)_4_(NO_3_)_2_ (mg) ^a)^ | Co(TU)_4_(NO_3_)_2_ (mg) ^a)^ | NPCTs (mg) | Ni atomic percent (%) | Co atomic percent (%) | Actual Ni-Co ratio applied abbreviation |
| --- | --- | --- | --- | --- | --- |
| 0 | 0 | 100 | 0 | 0 | NPCTs |
| 50 | 0 | 100 | 100 | 0 | NiS_2_@NPCTs |
| 40 | 10 | 100 | 67.9 | 32.1 | Ni_0.679_Co_0.321_S_2_@NPCTs |
| 30 | 20 | 100 | 44.4 | 55.6 | Ni_0.444_Co_0.556_S_2_@NPCTs |
| 20 | 30 | 100 | 26.1 | 73.9 | Ni_0.261_Co_0.739_S_2_@NPCTs |
| 10 | 40 | 100 | 13.5 | 86.5 | Ni_0.135_Co_0.865_S_2_@NPCTs |
| 0 | 50 | 100 | 0 | 100 | CoS_2_@NPCTs |

a) TU = thiourea

**Table S2.** Structural properties (BET surface area, pore volume, and average pore diameter) of the Ni_x_Co_1-x_S_2_@NPCTs and Ni_x_Co_1-x_S_2_@NPCTs/S composites.

| Sample | Surface area  (m^2^ g^-1^) | Pore volume  (cm^3^ g^-1^) | Pore diameter (nm) |
| --- | --- | --- | --- |
| Polypyrrole | 31.9 | 0.074 | 12.3 |
| NPCTs | 36.3 | 0.103 | 17.8 |
| NPCTs/S | 4.6 | 0.010 | 12.2 |
| NiS_2_@NPCTs | 40.2 | 0.108 | 15.1 |
| Ni_0.679_Co_0.321_S_2_@NPCTs | 41.5 | 0.113 | 14.3 |
| Ni_0.444_Co_0.556_S_2_@NPCTs | 44.4 | 0.114 | 13.3 |
| Ni_0.261_Co_0.739_S_2_@NPCTs | 48.5 | 0.113 | 13.0 |
| Ni_0.261_Co_0.739_S_2_@NPCTs/S | 4.6 | 0.013 | 15.3 |
| Ni_0.135_Co_0.865_S_2_@NPCTs | 43.6 | 0.115 | 14.2 |
| CoS_2_@NPCTs | 39.1 | 0.096 | 13.2 |

**Table S3.** Estimated lattice parameter information calculated from the Lab-scale XRD patterns.

| Sample | 2θ (⁰)^a)^ | Lattice parameter (Å) | *d*_200_ (Å) |
| --- | --- | --- | --- |
| NiS_2_ | 31.38 | 5.70 | 2.85 |
| Ni_0.679_Co_0.321_S_2_ | 31.68 | 5.64 | 2.82 |
| Ni_0.444_Co_0.556_S_2_ | 31.98 | 5.59 | 2.80 |
| Ni_0.261_Co_0.739_S_2_ | 32.08 | 5.58 | 2.79 |
| Ni_0.135_Co_0.865_S_2_ | 32.24 | 5.55 | 2.78 |
| CoS_2_ | 32.36 | 5.53 | 2.76 |

a) These 2θ data contain information about (200) directions.

**Table S4.** Calculated lattice information and refinement parameters of the Ni_x_Co_1-x_S_2_ catalysts from the HRPD data.

| Cell parameters | | | | |
| --- | --- | --- | --- | --- |
| Space group: *Pa*$\overline{3}$, *alpha* = *beta* = *gamma* = 90° | | | | |
| Lattice parameters | | | | |
| Sample name | Lattice parameter (Å) | | *d*_200_ (Å) | |
| Ni_0.679_Co_0.321_S_2_ | 5.64164 (± 0.00005) | | ca. 2.82 | |
| Ni_0.444_Co_0.556_S_2_ | 5.59003 (± 0.00026) | | ca. 2.80 | |
| Ni_0.261_Co_0.739_S_2_ | 5.57889 (± 0.00008) | | ca. 2.79 | |
| Ni_0.135_Co_0.865_S_2_ | 5.54713 (± 0.00006) | | ca. 2.77 | |
| Refinement parameters | | | | |
| Sample name | *R*_p_ | *R*_wp_ | *R*_exp_ | *χ*^2^ |
| Ni_0.679_Co_0.321_S_2_ | 14.5 | 14.5 | 6.09 | 5.70 |
| Ni_0.444_Co_0.556_S_2_ | 15.5 | 16.9 | 5.89 | 8.26 |
| Ni_0.261_Co_0.739_S_2_ | 19.8 | 20.6 | 6.54 | 9.91 |
| Ni_0.135_Co_0.865_S_2_ | 22.2 | 21.6 | 7.03 | 9.46 |

**Table S5.** Lattice parameters of relaxed bulk 2x2x2 cell calculated with computational method.

| Sample name | Lattice parameter (Å) |
| --- | --- |
| NiS_2_ | ca. 5.66 |
| Ni_0.25_Co_0.75_S_2_ | ca. 5.59 |
| CoS_2_ | ca. 5.55 |

**Table S6.** SAXS fitting parameters of the NPCTs, NiS_2_@NPCTs, Ni_0.261_Co_0.739_S_2_@NPCTs, and CoS_2_@NPCTs samples calculated from the core-shell cylinder model.

| Samples | Radius (Å) | Thickness  (Å) | Length (Å) | SLD_Core (ⅹ10^-6^ Å^-2^) | SLD_Shell (ⅹ10^-6^ Å^-2^) | *χ*^2^ |
| --- | --- | --- | --- | --- | --- | --- |
| NPCTs | 966.73  (± 0.13) | 378.05  (± 0.11) | 80651  (± 1.91) | 2.7517 | 3.2036 | 510.8 |
| NiS_2_@NPCTs | 1022.4  (± 0.05) | 335.53  (± 0.21) | 76614  (± 8.59) | 53.993 | 52.277 | 1159.4 |
| Ni_0.261_Co_0.739_S_2_@NPCTs | 936.69  (± 0.05) | 361.25  (± 0.14) | 73422  (± 8.26) | 11.233 | 14.806 | 847.57 |
| CoS_2_@NPCTs | 947.24  (± 0.03) | 353.67  (± 0.14) | 75594  (± 6.25) | 26.465 | 12.242 | 3448.3 |

**Table S7.** Curve fitting results for the Ni K-edge EXAFS spectra of the NiS_2_, Ni_0.261_Co_0.739_S_2_, nickel foil, and LiNiO_2_ powder samples.

| Samples | Bond | Coordination number | Debye-Waller factor σ^2^ (ⅹ 10^-3^ Å^2^) | Bond length (Å) | R-factor |
| --- | --- | --- | --- | --- | --- |
| NiS_2_ | Ni-S | 5.968 (± 0.096) | 6.229 (± 0.229) | 2.391 (± 0.004) | 0.0035635 |
|  | Ni-S | 5.985 (± 0.357) | 7.830 (± 1.050) | 3.502 (± 0.011) | 0.0035635 |
|  | Ni-Ni | 11.618 (± 1.109) | 17.840 (± 6.902) | 4.037 (± 0.049) | 0.0035635 |
| Ni_0.261_Co_0.739_S_2_ | Ni-S | 5.977 (± 0.136) | 6.323 (± 0.309) | 2.358 (± 0.005) | 0.0069647 |
|  | Ni-S | 6.200 (± 0.541) | 8.982 (± 2.297) | 3.445 (± 0.014) | 0.0069647 |
|  | Ni-TM | 11.882 (± 1.075) | 15.418 (± 3.100) | 3.918 (± 0.030) | 0.0069647 |
| Nickel foil | Ni-Ni | 11.812 (± 0.150) | 6.594 (± 0.118) | 2.485 (± 0.003) | 0.0022152 |
|  | Ni-Ni | 5.976 (± 0.619) | 10.738 (± 1.111) | 3.504 (± 0.011) | 0.0022152 |
|  | Ni-Ni | 24.005 (± 2.499) | 15.906 (± 23.570) | 4.253 (± 0.104) | 0.0022152 |
| LiNiO_2_ | Ni-O | 5.958 (± 0.141) | 9.932 (± 0.497) | 1.947 (± 0.006) | 0.0010773 |
|  | Ni-Ni | 6.005 (± 0.067) | 5.162 (± 1.166) | 2.887 (± 0.005) | 0.0010773 |
|  | Ni-Li | 6.006 (± 1.156) | 47.289 (± 10.535) | 1.205 (± 0.052) | 0.0010773 |

**Table S8.** Curve fitting results for the Co K-edge EXAFS spectra of the CoS_2_, Ni_0.261_Co_0.739_S_2_, cobalt foil, and LiCoO_2_ powder samples.

| Samples | Bond | Coordination number | Debye-Waller factor σ^2^ (ⅹ 10^-3^ Å^2^) | | Bond length (Å) | R-factor |
| --- | --- | --- | --- | --- | --- | --- |
| CoS_2_ | Co-S | 5.851 (± 0.112) | 6.560 (± 0.285) | | 2.307 (± 0.005) | 0.0048612 |
|  | Co-S | 6.114 (± 0.609) | 12.468 (± 2.746) | | 3.415 (± 0.018) | 0.0048612 |
|  | Co-Co | 11.956 (± 1.104) | 15.078 (± 2.777) | | 3.882 (± 0.017) | 0.0048612 |
| Ni_0.261_Co_0.739_S_2_ | Co-S | 5.874 (± 0.098) | 6.407 (± 0.260) | | 2.317 (± 0.004) | 0.0035193 |
|  | Co-S | 6.158 (± 0.565) | 13.284 (± 2.793) | | 3.429 (± 0.015) | 0.0035193 |
|  | Co-TM | 11.753 (± 1.349) | 19.410 (± 5.117) | | 3.920 (± 0.034) | 0.0035193 |
| Cobalt foil | Co-Co | 11.653 (± 0.533) | 7.303 (± 0.380) | | 2.495 (± 0.008) | 0.0314739 |
|  | Co-Co | 6.067 (± 1.790) | 9.426 (± 2.593) | | 3.529 (± 0.025) | 0.0314739 |
|  | Co-Co-Co | 40.718 (± 22.791) | 0.091 (± 6.076) | | 3.672 (± 0.048) | 0.0314739 |
| LiCoO_2_ | Co-O | 5.900 (± 0.173) | 3.783 (± 0.413) | | 1.919 (± 0.008) | 0.0040045 |
|  | Co-Co | 6.003 (± 0.168) | 5.033 (± 1.379) | | 2.820 (± 0.012) | 0.0040045 |
|  | Co-Li | 6.043 (± 0.491) | 3.087 (± 1.987) | 2.659 (± 0.028) | | 0.0040045 |

**Table S9**. Comparison of the electrochemical performances measured in this work with those of previously reported host cathode materials of Li-S batteries using similar metal sulfide electrocatalysts.

| Cathode materials | Rate and initial capacity (mAh g^-1^) | Sulfur loading (mg cm^-2^) | Capacity decay per cycle (%) | Reference |
| --- | --- | --- | --- | --- |
| Ni_0.261_Co_0.739_S_2_@NPCTs | 5.0 C/511 | 1.5 | 0.055 (1000 cycles) | Our work |
| NiS_2_/rGO | 1.0 C/952.6 | 1.06-1.59 | 0.072 (800 cycles) | ^[8]^ |
| yolk-shell NiS_2_/C | 1.0 C/744 | 1.0 | 0.2 (200 cycles) | ^[9]^ |
| NiCoS_4_-NiS_2_ NH@C | 2.0 C/889 | 0.8-1.2 | 0.065 (500 cycles) | ^[10]^ |
| Ni_3_S_2_ | 1.0 C/781.5 | 1.1 | 0.072 (400 cycles) | ^[11]^ |
| CoS_2_-TiO_2_@C | 1.0 C/898.2 | 0.8-1.1 | 0.06 (300 cycles) | ^[12]^ |
| CoS_2_-NHGC | 5.0 C/750 | 1-2 | 0.066 (800 cycles) | ^[13]^ |
| CoS/G | 0.5 C/607.6 | 1.0-1.5 | 0.338 (150 cycles) | ^[14]^ |
| CoS_2_/rGO-30 | 1.0 C/1217 | 1.27 | 0.04 (400 cycles) | ^[15]^ |
| CNS/N-rGO | 1.0 C/766 | 2.0 | 0.063 (500 cycles) | ^[16]^ |
| CoS_1.097_/MnS/NC@NC-600 | 1.0 C/890.7 | 0.8-1.2 | 0.11 (500 cycles) | ^[17]^ |
| ZnCo_2_S_4_ | 0.5 C/1146 | 1.1 | 0.268 (200 cycles) | ^[18]^ |
| MWCNT/Co_9_S_8_ | 0.1 C/1124 | 1.0 | 0.552 (100 cycles) | ^[19]^ |
| D-CoS2@NC/MWCNT | 1.0 C/637 | 1.0 | 0.066 (300 cycles) | ^[20]^ |
| H-LDH/Co_9_S_8_ | 1.0 C/700 | 1.5-2.0 | 0.047 (1500 cycles) | ^[21]^ |

**Table S10**. Expected gravimetric capacity and energy density values calculated from the galvanostatic charge-discharge profiles with the highy sulfur loaded (4.61 mg cm^-2^) Ni_0.261_Co_0.739_S_2_@NPCTs cathode at 0.2 C.

| Cycle condition | Expected C_cg_  (mAh g^-1^)^a^ | Expected C_tg_  (mAh g^-1^)^b^ | Expected E_g_  (mWh g^-1^)^c^ |
| --- | --- | --- | --- |
| 1st cycle | 353.3 | 320.7 | 673.5 |
| 100th cycle | 239.8 | 224.3 | 471.1 |
| 200th cycle | 227.9 | 213.9 | 449.2 |

a) C_cg_: Cathode gravimetric capacity

b) C_tg_: Total gravimetric capacity

c) E_g_: Gravimetric energy density

A. Assumptions:^[22]^

1. For the purpose of simplicity, only the mass and volume of active material, carbon and binder were considered.

2. Lithium utilization is 90%, and the anode degradation is not considered for simple calculation.

3. Carbon black and binder contribute no capacity.

B. Methods & Calculation

Average voltage for Li-S cell: V_a_ = ca. 2.1 V

1/C_tg_ = 1/C_ag_ + 1/C_cg_ & E_g_ = C_tg_ x V_a_

(C_tg_: total gravimetric capacity; C_ag_: anode gravimetric capacity; C_cg_: cathode gravimetric capacity, E_g_: gravimetric energy density; Va: average voltage of a cell)

**Table S11.** Fitting parameters of the Nyquist plots of the NPCTs, and Ni_x_Co_1-x_S_2_@NPCTs cathodes based on fitted equivalent circuits for different cycling conditions.

| Samples | *R*_0_ (ohm) | *R*_1_ (ohm) | *R*_2_ (ohm) | *R*_3_ (ohm) |
| --- | --- | --- | --- | --- |
| Before cycle | | | | |
| NPCTs | 2.9 | 68.5 | — | — |
| NiS_2_@NPCTs | 2.7 | 57.5 | — | — |
| Ni_0.679_Co_0.321_S_2_@NPCTs | 2.2 | 56.5 | — | — |
| Ni_0.444_Co_0.556_S_2_@NPCTs | 2.0 | 60.0 | — | — |
| Ni_0.261_Co_0.739_S_2_@NPCTs | 2.0 | 71.3 | — | — |
| Ni_0.135_Co_0.865_S_2_@NPCTs | 2.2 | 62.4 | — | — |
| CoS_2_@NPCTs | 2.3 | 71.5 | — | — |
| After 1 cycle at 1.0 C rate | | | | |
| NPCTs | 3.5 | 5.1 | 5.7 | 75.2 |
| NiS_2_@NPCTs | 3.0 | 7.0 | 7.0 | 275.7 |
| Ni_0.679_Co_0.321_S_2_@NPCTs | 3.4 | 6.3 | 5.7 | 384.4 |
| Ni_0.444_Co_0.556_S_2_@NPCTs | 3.3 | 5.3 | 7.0 | 197.6 |
| Ni_0.261_Co_0.739_S_2_@NPCTs | 2.3 | 8.3 | 2.2 | 19.2 |
| Ni_0.135_Co_0.865_S_2_@NPCTs | 3.0 | 7.8 | 3.1 | 26.8 |
| CoS_2_@NPCTs | 3.3 | 9.5 | 2.2 | 26.0 |
| After 500 cycles at 1.0 C rate | | | | |
| NPCTs | 2.9 | 7.9 | 5.3 | 1076.0 |
| NiS_2_@NPCTs | 3.7 | 11.2 | 5.2 | 1115.0 |
| Ni_0.679_Co_0.321_S_2_@NPCTs | 3.8 | 2.5 | 3.4 | 532.7 |
| Ni_0.444_Co_0.556_S_2_@NPCTs | 1.9 | 1.5 | 4.2 | 53.2 |
| Ni_0.261_Co_0.739_S_2_@NPCTs | 4.4 | 0.5 | 2.3 | 42.3 |
| Ni_0.135_Co_0.865_S_2_@NPCTs | 3.7 | 3.6 | 3.8 | 196.7 |
| CoS_2_@NPCTs | 3.3 | 4.8 | 2.9 | 151.2 |

**Table S12.** Fitting parameters of the various Ni_0.261_Co_0.739_S_2_@NPCTs cathodes for different cycling conditions based on the Guinier-Porod evaluation.

| Samples | Porod exponential | *R*_g_ (Å) | *s* | *χ*^2^ |
| --- | --- | --- | --- | --- |
| Pristine | 3.4669 (± 0.0045) | 2.6674 (± 0.0030) | 2.7009 (± 0.0002) | 477.53 |
| 500 cycled@1.0C | 3.2688 (± 0.0020) | 3.5261 (± 0.0029) | 2.6304 (± 0.0002) | 388.65 |
| 1000 cycled@5.0C | 3.0923 (± 0.0011) | 3.3567 (± 0.0059) | 2.6454 (± 0.0002) | 375.70 |
| 200 cycled@0.2C of 2.69 mg cm^-2^ | 3.7889 (± 0.0049) | 3.9976 (± 0.0024) | 2.5480 (± 0.0001) | 612.65 |
| 200 cycled@0.2C of 4.61 mg cm^-2^ | 3.292 (± 0.0051) | 3.9046 (± 0.0024) | 2.8199 (± 0.0001) | 949.36 |

**Table S13.** Determination of the growth type and discharge capacity of Li_2_S precipitation reaction for the NPCTs, and Ni_x_Co_1-x_S_2_@NPCTs cathodes with recorded *I*_m_ and *t*_m_ values from current-time transients.

| Cathode composite | *I*_m_ (A g^-1^) | *t*_m_ (s) | Nuclei growth type | Li_2_S capacity (mAh g^-1^) |
| --- | --- | --- | --- | --- |
| NPCTs | 1.139 | 1557 | 2DI | 375 |
| NiS_2_@NPCTs | 1.303 | 642 | 3DP | 443 |
| Ni_0.679_Co_0.321_S_2_@NPCTs | 1.326 | 606 | 3DP | 465 |
| Ni_0.444_Co_0.556_S_2_@NPCTs | 1.257 | 948 | 3DP | 486 |
| Ni_0.261_Co_0.739_S_2_@NPCTs | 0.797 | 1446 | 3DI-3DP | 528 |
| Ni_0.135_Co_0.865_S_2_@NPCTs | 1.084 | 1632 | 2DP-2DI | 484 |
| CoS_2_@NPCTs | 1.604 | 1029 | 2DP-2DI | 438 |

**Table S14.** Curve fitting results for the Ni K-edge EXAFS spectra of the NiS_2_, Ni_0.261_Co_0.739_S_2_ cathodes in pristine and 100 cycled states.

| Samples | Bond | Coordination number | Debye-Waller factor σ^2^ (ⅹ10^-3^ Å^2^) | Bond length (Å) | R-factor |
| --- | --- | --- | --- | --- | --- |
| Pristine | | | | | |
| NiS_2_ | Ni-S | 5.922 (± 0.120) | 6.933 (± 0.472) | 2.388 (± 0.010) | 0.0056274 |
|  | Ni-S | 6.006 (± 0.208) | 1.754 (± 7.379) | 3.521 (± 0.045) | 0.0056274 |
|  | Ni-Ni | 11.977 (± 1.274) | 17.260 (± 3.879) | 4.000 (± 0.029) | 0.0056274 |
| Ni_0.261_Co_0.739_S_2_ | Ni-S | 5.937 (± 0.100) | 5.668 (± 0.307) | 2.359 (± 0.005) | 0.0044928 |
|  | Ni-S | 5.994 (± 0.206) | 3.012 (± 3.901) | 3.463 (± 0.026) | 0.0044928 |
|  | Ni-TM | 12.083 (± 0.571) | 10.080 (± 3.357) | 3.895 (± 0.024) | 0.0044928 |
| After 100 cycles | | | | | |
| NiS_2_ | Ni-S | 5.897 (± 0.106) | 7.005 (± 0.419) | 2.387 (± 0.006) | 0.0040860 |
|  | Ni-S | 6.011 (± 0.296) | 7.423 (± 2.642) | 3.485 (± 0.018) | 0.0040860 |
|  | Ni-Ni | 12.217 (± 1.265) | 48.234 (± 56.908) | 3.967 (± 0.335) | 0.0040860 |
| Ni_0.261_Co_0.739_S_2_ | Ni-S | 5.856 (± 0.113) | 5.580 (± 0.323) | 2.361 (± 0.006) | 0.0063397 |
|  | Ni-S | 5.979 (± 0.252) | 3.371 (± 2.962) | 3.523 (± 0.028) | 0.0063397 |
|  | Ni-TM | 12.018 (± 1.135) | 16.079 (± 4.153) | 3.922 (± 0.023) | 0.0063397 |

**Table S15.** Curve fitting results for the Co K-edge EXAFS spectra of the CoS_2_, Ni_0.261_Co_0.739_S_2_ cathodes in pristine and 100 cycled states.

| Samples | Bond | Coordination number | Debye-Waller factor σ^2^ (ⅹ10^-3^ Å^2^) | Bond length (Å) | R-factor |
| --- | --- | --- | --- | --- | --- |
| Pristine | | | | | |
| CoS_2_ | Co-S | 5.874 (± 0.156) | 9.186 (± 0.411) | 2.241 (± 0.007) | 0.0136424 |
|  | Co-S | 5.837 (± 0.328) | 3.706 (± 4.922) | 3.372 (± 0.043) | 0.0136424 |
|  | Co-Co | 11.695 (± 0.275) | 3.719 (± 5.558) | 3.791 (± 0.046) | 0.0136424 |
| Ni_0.261_Co_0.739_S_2_ | Co-S | 5.817 (± 0.153) | 7.063 (± 0.552) | 2.300 (± 0.010) | 0.0089260 |
|  | Co-S | 6.062 (± 0.362) | 5.920 (± 5.125) | 3.311 (± 0.029) | 0.0089260 |
|  | Co-TM | 11.995 (± 0.203) | 1.134 (± 3.988) | 3.798 (± 0.023) | 0.0089260 |
| After 100 cycles | | | | | |
| CoS_2_ | Co-S | 5.972 (± 0.114) | 8.323 (± 0.274) | 2.243 (± 0.005) | 0.0079393 |
|  | Co-S | 5.979 (± 0.538) | 10.750 (± 5.998) | 3.340 (± 0.034) | 0.0079393 |
|  | Co-Co | 12.140 (± 0.156) | 2.589 (± 2.195) | 3.710 (± 0.022) | 0.0079393 |
| Ni_0.261_Co_0.739_S_2_ | Co-S | 5.897 (± 0.160) | 6.614 (± 0.573) | 2.303 (± 0.010) | 0.0092682 |
|  | Co-S | 6.050 (± 0.476) | 7.818 (± 7.662) | 3.340 (± 0.041) | 0.0092682 |
|  | Co-TM | 11.907 (± 0.237) | 1.577 (± 4.935) | 3.818 (± 0.026) | 0.0092682 |

**References**

[1] G. Kresse, J. Furthmüller, *Phys. Rev. B - Condens. Matter Mater. Phys.* **1996**, *54*, 11169.

[2] G. Kresse, J. Hafner, *Phys. Rev. B* **1993**, *47*, 558.

[3] P. E. Blöchl, *Phys. Rev. B* **1994**, *50*, 17953.

[4] B. Hammer, L. B. Hansen, J. K. Nørskov, *Phys. Rev. B - Condens. Matter Mater. Phys.* **1999**, *59*, 7413.

[5] J. Liu, S. H. Xiao, Z. Zhang, Y. Chen, Y. Xiang, X. Liu, J. S. Chen, P. Chen, *Nanoscale* **2020**, *12*, 5114.

[6] S. Grimme, J. Antony, S. Ehrlich, H. Krieg, *J. Chem. Phys.* **2010**, *132*.

[7] S. Steinberg, R. Dronskowski, *Crystals* **2018**, *8*.

[8] Y. Li, J. Chen, Y. Zhang, Z. Yu, T. Zhang, W. Ge, L. Zhang, *J. Alloys Compd.* **2018**, *766*, 804.

[9] Y. Tian, H. Huang, G. Liu, R. Bi, L. Zhang, *Chem. Commun.* **2019**, *55*, 3243.

[10] Q. Chen, J. Zhou, Y. Zhu, C. Jin, J. Zhang, Y. Wu, W. Tang, *Energy and Fuels* **2023**, *37*, 4711.

[11] Y. Yan, Y. Chen, Z. Wang, C. Qin, Z. Bakenov, Y. Zhao, *Microporous Mesoporous Mater.* **2021**, *326*, 111355.

[12] D. Li, H. Li, S. Zheng, N. Gao, S. Li, J. Liu, L. Hou, J. Liu, B. Miao, J. Bai, Z. Cui, N. Wang, B. Wang, Y. Zhao, *J. Colloid Interface Sci.* **2022**, *607*, 655.

[13] F. Lei, Y. Cao, R. Wang, Z. Zhang, S. Qiu, *Electrochim. Acta* **2023**, *468*, 143054.

[14] Z. Yu, N. Zhang, X. Zhang, Y. Li, G. Xie, W. Ge, L. Zhang, T. Zhang, *J. Electroanal. Chem.* **2019**, *854*, 113524.

[15] Y. Li, J. Li, J. Yuan, Y. Zhao, J. Zhang, H. Liu, F. Wang, J. Tang, J. Song, *J. Alloys Compd.* **2021**, *873*, 159780.

[16] P. Wu, H. Y. Hu, N. Xie, C. Wang, F. Wu, M. Pan, H. F. Li, X. Di Wang, Z. Zeng, S. Deng, G. P. Dai, *RSC Adv.* **2019**, *9*, 32247.

[17] X. Kang, Y. Dong, H. Guan, M. A. Al-Tahan, J. Zhang, *J. Colloid Interface Sci.* **2022**, *622*, 515.

[18] H. Zhang, J. Liu, X. Lin, Y. Zhong, J. Ren, Z. Wang, T. Han, J. Li, *Ceram. Int.* **2020**, *46*, 14056.

[19] W. Tong, Y. Huang, W. Jia, X. Wang, Y. Guo, Z. Sun, D. Jia, J. Zong, *J. Alloys Compd.* **2018**, *731*, 964.

[20] C. H. Chen, S. H. Lin, Y. J. Wu, J. T. Su, C. C. Cheng, P. Y. Cheng, Y. C. Ting, S. Y. Lu, *Chem. Eng. J.* **2022**, *431*, 133924.

[21] S. Chen, J. Luo, N. Li, X. Han, J. Wang, Q. Deng, Z. Zeng, S. Deng, *Energy Storage Mater.* **2020**, *30*, 187.

[22] J. Gao, H. D. Abruña, *J. Phys. Chem. Lett.* **2014**, *5*, 882.
